# Supplementary material for: Do socioeconomic inequalities contribute to the high prevalence of child developmental risk in an ethnically diverse, socioeconomically disadvantaged population? A Born in Bradford’s Better Start (BiBBS) study
Source: BMJ Paediatr Open. 2026 Jan 23;10(1):e003770. doi: 10.1136/bmjpo-2025-003770 (PMC12853529; doi:10.1136/bmjpo-2025-003770)
Supplement: Supplementary data [file bmjpo-10-1-s002.pdf]

Variable	Not at risk (n=1,558, 56%)	At risk (FGLD) (n=884, 44%)	Total sample (n=2,003, 100%)
**Categorical variables N (%)**			
**Maternal education**			
No qualifications	70 (6)	65 (7)	135 (7)
5 or less GCSEs	234 (21)	154 (17)	388 (19)
5 or more GCSEs	94 (8)	68 (8)	162 (8)
A levels	87 (8)	61 (7)	148 (7)
Degree	273 (24)	141 (16)	414 (21)
Missing	*361 (32)*	*395 (45)*	*756 (38)*
**Financial security**			
Finding it quite/very difficult	46 (4)	48 (5)	94 (4)
Just about getting by	132 (12)	115 (5)	247 (12)
Doing alright	368 (33)	290 (33)	658 (33)
Living comfortably	324 (29)	189 (21)	513 (26)
Missing	*249 (22)*	*242 (27)*	*491 (25)*
**Maternal ethnicity**			
White British	95 (8)	76 (9)	171 (9)
Pakistani	581 (52)	398 (45)	979 (49)
South Asian	79 (7)	64 (7)	143 (7)
White Other	42 (4)	23 (3)	65 (3)
Other	91 (8)	60 (7)	151 (8)
Missing	*231 (21)*	*263 (30)*	*494 (25)*
**Maternal county of birth**			
UK	415 (37)	322 (36)	737 (37)
Not UK	476 (43)	304 (34)	780 (39)
Missing	*228 (20)*	*258 (19)*	*486 (24)*
**Maternal language spoken**			
English	514 (46)	354 (40)	868 (43)
Not English	354 (32)	254 (29)	608 (30)
Missing	*251 (22)*	*276 (31)*	*527 (26)*
**Child sex**			
Male	621 (55)	375 (42)	996 (50)

Female	498 (45)	509 (58)	1007 (50)
Missing	*0 (0)*	*0 (0)*	*0 (0)*
**Continuous variables M (SD)**			
**Child age in months (n=2003)**	28 (3.10)	29 (3.78)	28.62 (3.45)
**Social ladder (n=837)**	6.27 (1.79)	6.03 (1.72)	6.17 (1.76)
**People to count on (n=1546)**	3.29 (2.49)	3.27 (2.44)	3.28 (2.47)

Supplementary table. Descriptives for all participants by whether they were categorised as at risk or not, using the FGLD method.

Variable	Other	South Asian	White British	Total
**Categorical variables N (%)**				
**Maternal education**				
No qualifications	23 (11)	93 (8)	19 (11)	135 (9)
5 or less GCSEs	49 (23)	287 (26)	51 (30)	387 (26)
5 or more GCSEs	16 (7)	102 (9)	44 (26)	162 (11)
A levels	18 (8)	116 (10)	14 (8)	148 (10)
Degree	42 (19)	338 (30)	33 (19)	413 (27)
Missing	68 (31)	186 (17)	10 (6)	264 (17)
**Financial security**				
Finding it quite/very difficult	22 (10)	59 (5)	6 (4)	87 (6)
Just about getting by	36 (17)	159 (14)	37 (22)	232 (15)
Doing alright	98 (45)	447 (40)	77 (45)	622 (41)
Living comfortably	44 (20)	406 (36)	46 (26)	496 (33)
Missing	16 (7)	51 (5)	5 (3)	72 (5)
**Maternal county of birth**				
UK	45 (21)	517 (46)	170 (99)	732 (49)
Not UK	169 (78)	605 (54)	1 (<1)	775 (51)
Missing	2 (<1)	0 (0)	0 (0)	2 (<1)
**Maternal language spoken**				
English	77 (36)	615 (55)	171(100)	863
Not English	111 (51)	493 (44)	0 (0)	604
Missing	28 (13)	14 (1)	0 (0)	42
**Child sex**				
Male	114 (53)	571 (49)	85 (50)	770 (49)
Female	102 (47)	551 (51)	86 (5)	739 (51)
Missing	0 (0)	0 (0)	0 (0)	0 (0)
**Continuous variables M (SD)**				
**Child age in months (n=1508)**	28.49 (3.73)	28.01 (3.21)	28.49 (4.93)	28.13 (3.53)
**Social ladder (n=835)**	5.57 (2.07)	6.36 (1.73)	5.71 (1.44)	6.17 (1.76)
**People to count on (n=1469)**	2.69 (2.09)	3.33 (2.53)	3.70 (2.36)	3.28 (2.47)

Supplementary table. Descriptives for all participants by ethnic groups used in Objective C.
