# Supplementary material for: Do socioeconomic inequalities contribute to the high prevalence of child developmental risk in an ethnically diverse, socioeconomically disadvantaged population? A Born in Bradford’s Better Start (BiBBS) study
Source: BMJ Paediatr Open. 2026 Jan 23;10(1):e003770. doi: 10.1136/bmjpo-2025-003770 (PMC12853529; doi:10.1136/bmjpo-2025-003770)
Supplement: Supplementary data [file bmjpo-10-1-s003.pdf]

Predictor (n in model, R^2)	Odds ratio (95% CI)	*p*
**Maternal education (n=1,214, R^2=0.046)**		
No qualifications (ref)	.	.
5 or less GCSEs	1.54 (1.02 to 2.34)	0.040*
5 or more GCSEs	1.62 (0.98 to 2.68)	0.059
A levels	1.38 (0.82 to 2.33)	0.224
Degree	1.95 (1.28 to 2.99)	0.002*
**Financial security (n=1,159, R^2=0.049)**		
Finding it quite/very difficult (ref)		
Just about getting by	1.35 (0.76 to 2.42)	0.305
Doing alright	1.44 (0.84 to 2.46)	0.181
Living comfortably	1.78 (1.03 to 3.07)	0.037*
**Social status (ladder) (n=791, R^2=0.056)**	1.11 (1.02 to 1.22)	0.019*
**Social support (people to count on) (n=1,191, R^2=0.045)**	0.99 (0.94 to 1.04)	0.655

Supplementary Table. Odds ratios for adjusted logistic regression models, with no interaction for ethnicity included.

Note: *each model contained the relevant adjustment sets as presented in Table 1, though these coefficients are not presented as these coefficients can be misinterpreted (Table 2 Fallacy, see [39]).*

Adjustment sets for each model were as follows: (1) maternal education exposure included Migrant to UK, English is first language, Ethnic group, (2) financial security exposure included Maternal education, Migrant to UK, English is first language, Ethnic group, (3) social status exposure included Maternal education, Migrant to UK, English is first language, Ethnic group, (3) social support exposure included Maternal education, Migrant to UK, English is first language, Ethnic group. All models adjusted for child age and child sex.
