# Supplementary material for: Do socioeconomic inequalities contribute to the high prevalence of child developmental risk in an ethnically diverse, socioeconomically disadvantaged population? A Born in Bradford’s Better Start (BiBBS) study
Source: BMJ Paediatr Open. 2026 Jan 23;10(1):e003770. doi: 10.1136/bmjpo-2025-003770 (PMC12853529; doi:10.1136/bmjpo-2025-003770)
Supplement: Supplementary data [file bmjpo-10-1-s004.pdf]

Predictor	Odds ratio (95% CI)	p
**Maternal education (n=1214, R^2=0.052)**		
No qualifications (ref)	.	.
5 or less GCSEs	2.07 (0.70 to 6.48)	0.193
5 or more GCSEs	1.49 (0.49to 4.74)	0.485
A levels	3.47 (0.81 to 17.17)	0.105
Degree	4.90 (1.48 – 17.57)	0.011*
**Financial security (n=1159, R^2=0.052)**		
Finding it quite/very difficult (ref)		
Just about getting by	1.38 (0.22 to 8.54)	0.720
Doing alright	1.06 (0.18 to 6.14)	0.943
Living comfortably	2.01 (0.33 to 12.33)	0.432
**Social status (ladder) (n=791, R^2=0.058)**	1.21 (0.93 to 1.58)	0.168
**Social support (people to count on) (n=1191, R^2=0.050)**	1.07 (0.93 to 1.24)	0.336

Supplementary table. Model results when interaction by ethnicity is included.

Note: Adjustment sets for each model were as follows: (1) maternal education exposure included Migrant to UK, English is first language, Ethnic group, (2) financial security exposure included Maternal education, Migrant to UK, English is first language, Ethnic group, (3) social status exposure included Maternal education, Migrant to UK, English is first language, Ethnic group, (3) social support exposure included Maternal education, Migrant to UK, English is first language, Ethnic group. All models adjusted for child age and child sex.
