# Supplementary material for: Do socioeconomic inequalities contribute to the high prevalence of child developmental risk in an ethnically diverse, socioeconomically disadvantaged population? A Born in Bradford’s Better Start (BiBBS) study
Source: BMJ Paediatr Open. 2026 Jan 23;10(1):e003770. doi: 10.1136/bmjpo-2025-003770 (PMC12853529; doi:10.1136/bmjpo-2025-003770)
Supplement: Author's manuscript [file bmjpo-2025-003770.draft_revisions.pdf]

BMJ Paediatrics Open

BMJ Paediatrics Open is committed to open peer review. As part of this commitment we make the peer review history of every article we publish publicly available.

When an article is published we post the peer reviewers' comments and the authors' responses online. We also post the versions of the paper that were used during peer review. These are the versions that the peer review comments apply to.

The versions of the paper that follow are the versions that were submitted during the peer review process. They are not the versions of record or the final published versions. They should not be cited or distributed as the published version of this manuscript.

BMJ Paediatrics Open is an open access journal and the full, final, typeset and author-corrected version of record of the manuscript is available on our site with no access controls, subscription charges or pay-per-view fees (http://bmjpaedsopen.bmj.com).

If you have any questions on BMJ Paediatrics Open's open peer review process please email info.bmjpo@bmj.com

BMJ Paediatrics Open

Do socioeconomic inequalities contribute to the high prevalence of child developmental risk in an ethnically diverse, socioeconomically disadvantaged population? A Born in Bradford's Better Start (BiBBS) study

Journal:	*BMJ Paediatrics Open*
Manuscript ID	bmjpo-2025-003770
Article Type:	Original research
Date Submitted by the Author:	23-Jun-2025
Complete List of Authors:	Mooney, Kate; University of York, Health Sciences; Bradford Institute for Health Research,<br>Dickerson, Josie ; Bradford Institute for Health Research<br>Blower, Sarah Louise; University of York, Health Sciences<br>Walker, Matthew; Bradford Institute for Health Research<br>Lister, Jennie; University of York, Health Sciences<br>Pickett, Kate; University of York, Health Sciences
Keywords:	Child Health, Epidemiology

SCHOLARONE™
Manuscripts

I, the Submitting Author has the right to grant and does grant on behalf of all authors of the Work (as defined in the below author licence), an exclusive licence and/or a non-exclusive licence for contributions from authors who are: i) UK Crown employees; ii) where BMJ has agreed a CC-BY licence shall apply, and/or iii) in accordance with the terms applicable for US Federal Government officers or employees acting as part of their official duties; on a worldwide, perpetual, irrevocable, royalty-free basis to BMJ Publishing Group Ltd ("BMJ") its licensees and where the relevant Journal is co-owned by BMJ to the co-owners of the Journal, to publish the Work in this journal and any other BMJ products and to exploit all rights, as set out in our licence.

The Submitting Author accepts and understands that any supply made under these terms is made by BMJ to the Submitting Author unless you are acting as an employee on behalf of your employer or a postgraduate student of an affiliated institution which is paying any applicable article publishing charge ("APC") for Open Access articles. Where the Submitting Author wishes to make the Work available on an Open Access basis (and intends to pay the relevant APC), the terms of reuse of such Open Access shall be governed by a Creative Commons licence – details of these licences and which Creative Commons licence will apply to this Work are set out in our licence referred to above.

Other than as permitted in any relevant BMJ Author's Self Archiving Policies, I confirm this Work has not been accepted for publication elsewhere, is not being considered for publication elsewhere and does not duplicate material already published. I confirm all authors consent to publication of this Work and authorise the granting of this licence.

Confidential: For Review Only

TITLE PAGE

Do socioeconomic inequalities contribute to the high prevalence of child developmental risk in an ethnically diverse, socioeconomically disadvantaged population? A Born in Bradford's Better Start (BiBBS) study

Kate E. Mooney (1,2)*, Josie Dickerson (2), Sarah L. Blower (1), Matthew Walker (2), Jennie Lister (1), and Kate E. Pickett (1).

1. Department of Health Sciences, University of York
2. Bradford Institute for Health Research, Bradford Teaching Hospital Foundation Trust

**=corresponding author*

Confidential: For Review Only

ABSTRACT

Background

Socioeconomic inequalities in child development are pervasive, however, less is known regarding the impacts of socioeconomic factors within and across ethnically diverse, socioeconomically disadvantaged populations. This study (1) describes the prevalence of children at risk of poor overall early child development; (2) investigates the relationship between individual indicators of socioeconomic position and early child development; and (3) investigates if the relationship between indicators of socioeconomic position and early child development varies by ethnic group.

Methods

This study uses data from a prospective birth cohort study, Born in Bradford's Better Start (BiBBS). Child development was measured with the Ages and Stages Questionnaire (ASQ) during routine health visiting at age 2-years-old. Binary logistic regression investigated child development by key maternal socioeconomic indicators; maternal education, financial security, social status (measured via the MacArthur Scale of Subjective Social Status), and social support (measured via number of people to count on).

Results

A high proportion of the 2,003 children with a valid developmental were at risk of poor child development (22%). Mothers who had a degree (OR=2.09, 95% CI 1.33 to 3.30), reported 'living comfortably' in financial security (OR=1.90, 95% CI 1.08 to 3.36), and had higher social status (OR=1.13, 1.03 to 1.24) all had higher odds of their child having a good development. Though socioeconomic gradients in maternal education and financial security were consistent across White British, South Asian, and Other ethnic groups, both social support and social status had weaker relationships with child development for South Asian parents.

Conclusion

A high proportion of children are at risk of poor development in this diverse, socioeconomically disadvantaged population. Higher socioeconomic position may protect against poor early development, and the mechanisms underlying this may differ by ethnicity. The findings underline the need for proportionate universal strategies to improve child development in such communities.

Confidential: For Review Only

What is already known on this topic

- Socioeconomic inequalities in early child development are pervasive, however, there is less research on the impact of these factors within and across ethnically diverse, socioeconomically disadvantaged populations.
- Whilst traditional socioeconomic indicators such as higher parental education are established predictors of child development, social support and status could also improve children's development through alleviation of challenges during the transition to parenthood, however, few studies have examined these indicators.
- This study of children living in inner-city Bradford allows an examination of socioeconomic gradients within a socioeconomically deprived, ethnically diverse sample.

What this study adds

- A high proportion (22%) of children in the Born in Bradford's Better Start (BiBBS) study population are at risk of poor child development.
- Higher maternal education, financial security, and social status are associated with better child development, with the steepest socioeconomic gradient by social status
- The relationships between social support and social status with child development varied by ethnic group, as these indicators did not relate to child development for South Asian parents.

How this study might affect research, practice or policy

- The BiBBS cohort can be used to further understand patterns of childhood inequalities in socioeconomically deprived, ethnically diverse populations
- Future research should focus on the nuances of how and why socioeconomic factors impact child development within specific ethnic groups, particularly within socioeconomically disadvantaged populations
- A proportionate universal approach, with both universal prevention strategies available to all parents and targeted intervention programs for those in need of additional support, is recommended to support communities and improve children's outcomes.

Confidential: For Review Only

INTRODUCTION

The first three years of life are a critical period for childhood development, which encompasses the development of physical, mental, social, and emotional capabilities [1,2]. Early childhood development promotes future educational achievement and health [3,4], and persistent difficulties with early cognitive and socioemotional development can lead to worse physical and mental health in late adolescence [5]. Prevention and early intervention to promote strong early development for all children may reduce socioeconomic disparities across the lifespan [6].

Socioeconomic position and child development

Health inequalities are unfair and avoidable differences related to personal, demographic and socioeconomic characteristics. The majority of the relevant literature demonstrating pervasive socioeconomic inequalities in early child development in the UK is based on cohort studies [7]. The Millennium Cohort Study (MCS) (N=19,517), is a nationally representative sample of children born 2000–2002. In MCS, outcomes examined at age 3 include socioemotional difficulties measured via the Strengths and Difficulties Questionnaire (SDQ) and cognitive development measured via the Bracken Basic Concept Scale. At age 5, socioemotional difficulties were measured again via the SDQ, and verbal ability via the British Ability Scales. In MCS, higher parental education predicted higher verbal ability at age 5 [8], and higher family socioeconomic position predicted lower socioemotional difficulties at ages 3 and 5 [9]. Families with the lowest incomes had children with a higher prevalence of both socioemotional and cognitive difficulties at both 3-years-old and 5-years-old [10]. A study using the Avon Longitudinal Study of Parents and Children (ALSPAC, N=13,855) similarly reported that lower parental occupation, education, and financial security were associated with lower expressive and receptive language at 2 years old measured as a modification of the MacArthur Communicative Development Inventory (CDI) [11].

Socioeconomic position may impact children’s development through multiple mechanisms, for instance, socioeconomic advantage may afford parents better resources and higher education for enhancing child development through cognitive stimulation and more complex language use, whereas socioeconomic disadvantage may have a negative impact upon children’s development through poor parental mental health and stress impacting upon parental engagement [12,13]. It is therefore of interest to compare the magnitude of associations for multiple measures, including not only traditional measures such as income and education, but also subjective aspects of societal position [14]. This can give insights into the mechanisms by which family socioeconomic position influences children’s development [15].

Subjective measures

For socioeconomically deprived families, subjective measures may be more sensitive predictors of children’s outcomes than measures of income, as they can more accurately assess whether lower-income families feel they can provide for basic needs [15]. For example, in the Born in Bradford cohort, parents' self-reported financial security more strongly predicted birth outcomes, in comparison to parental education, receipt of means-tested benefits, and paternal occupation [16]. Further, subjective social status indicators such as the Macarthur Scale of Subjective Social Status may encourage consideration of perceptions of relative inequality, and this has been associated with cognitive function and self-rated health in adulthood [17]. Within the sample used in this study, most pregnant women rate themselves as having higher social status than others in their neighbourhood, despite living in socioeconomically deprived areas [18]. Whilst self-reported social status appears to be related to adult wellbeing [19], it is yet to be investigated with regards to children’s outcomes.

Confidential: For Review Only

Another subjective measure of socioeconomic position is parental social support, which refers to the existence of social networks and could alleviate challenges during the transition to parenthood, hence supporting childhood outcomes [20]. Emerging evidence suggests that higher levels of social support during pregnancy are associated with improved cognitive development at age 2 [21], and lower prevalence of developmental delay at age 3 [22].

Ethnicity and child development

As ethnic minority groups tend to experience higher levels of socioeconomic disadvantage in England [23], a consideration of differences in child development by ethnicity might reveal differential impacts of socioeconomic position. Indeed, an MCS study revealed that most ethnic minority groups have lower scores in cognitive and socioemotional outcomes compared to White British children [24]. A study of routinely collected health visiting data between 2018-2021, with a sample of 226,505 children at age 2-2 ½ years, found that 86% of children met expected levels of development overall, but that children from the most deprived neighbourhoods (82.6%), and children recorded as Black ethnicity (78.9%) were less likely to meet expected level of development [25].

Socioeconomic gradients within ethnic groups

Previous research has demonstrated that socioeconomic differences appear to be less pronounced in ethnic minority groups than ethnic majority groups for maternal and child health outcomes [16,26], and child socioemotional difficulties [27] and working memory [28]. This lack of socioeconomic gradient may be due to difficulties with measurement, for instance inaccurate measurements of socioeconomic position in ethnic minority groups (e.g., educational attainment if received in a different country), or smaller samples in minority groups being underpowered to detect associations. Or, these findings may be due to a true lack of relationship between socioeconomic position and children's outcomes for ethnic minority groups. Though, the relationship between ethnicity and children's outcomes is likely to be influenced by a variety of other factors beyond socioeconomic position, such as differences in culture, and experiences of prejudice, discrimination and racism [29]. Hence, larger samples of ethnic minorities, and a variety of socioeconomic measures, are needed to investigate the true impacts of socioeconomic position across diverse ethnic groups.

Present study and rationale

The present study uses data from the Born in Bradford's Better Start (BiBBS) interventional birth cohort, which has recruited a representative sample of 5,758 pregnant mothers and their children living in inner-city areas of Bradford. The mothers in the BiBBS cohort are predominantly from ethnic minority backgrounds (88%), and living in the most deprived decile of the Index of Multiple Deprivation (84%) [30].

This under-researched population provides the opportunity to understand potential differences in children's development by both subjective and objective socioeconomic measures, enabling an investigation of whether socioeconomic differences exist within disadvantaged contexts, and among different ethnic groups. This may also provide insights into which factors may protect against the development of poor childhood development in a predominantly socioeconomically disadvantaged population, and which groups children may need more support. In this study we:

1. Describe the prevalence of children at risk of poor child development overall

2. Investigate which individual indicators of socioeconomic position predict early child development

Confidential: For Review Only

3. Investigate if the association between individual indicators of socioeconomic position and early child development varies by ethnic group

Confidential: For Review Only

METHODS

Study design

This is a secondary analysis of observational cohort study data. We have used the Strengthening the Reporting of Observational Studies in Epidemiology (STROBE) (2021) guidelines for reporting methods (see Supplementary File A) [31].

Study setting

This study is set in Bradford, a city in Northern England with high levels of socioeconomic deprivation and a large ethnic minority population [32]. Part of the Born in Bradford (BiB) research programme, Born in Bradford's Better Start (BiBBS) captures in-depth baseline data during pregnancy, and consent for routine linkage to mothers' and their children's health, education, and intervention participation records. Pregnant women were invited to join BiBBS by trained researchers. Recruitment took place predominantly at Bradford Royal Infirmary's Glucose Tolerance Test (GTT) clinic at 24-28 weeks gestation which is offered to all women booked for delivery at BRI, and secondary recruitment sources in the community [30,33].

Data

All predictive measures were measured during the BiBBS baseline questionnaire, administered when mothers were approximately 26 weeks pregnant with their child. We linked Health Visiting records to provide the Ages and Stages Questionnaire (ASQ) outcome for this study. Inclusion for this study was all recruited BiBBS pregnancies between January 2016 and December 2022, who had an ASQ recorded for that pregnancy at the 2-2 ½ year visit by October 2024 (the time of the data drop from the data source).

Inclusion of covariates was decided using a Directed Acyclic Graph (DAG) (see below), and the analytical form of variables is described below.

Measures

We used four indicators of socioeconomic position. First, maternal education was categorised into 'No qualifications', '5 or less GCSEs', '5 or more GCSEs', 'A levels', and 'Degree'. Second, financial security was ascertained by mother's response to "how well are you managing financially?" Responses were coded as: 'Just about getting by', 'Finding it quite/very difficult', 'Doing alright', 'Living comfortably'. Third, subjective social status was measured via the MacArthur Scale of Subjective Social Status, an image of a ladder with 10 rungs, numbered from 1 at the bottom to 10 at the top. We used mother's responses to "Where would you place yourself on the ladder in relation to other people in your neighbourhood?", with answers analysed as continuous, ranging between 0 and 10. Fourth, social support was measured via responses to "how many people in your life can you count on in times of need?", again analysed as a continuous predictor, and answers ranged from 0 to 10. To test the sensitivity of the results to the linearity assumption, we estimated additional models with social status and social support estimated as categorical variables, categorised into low (0-2 people, 1-2 ladder), medium (3-5 people, 3-8 ladder) and high (6 to 10 people, 9-10 ladder). Any 'do not wish to answer' and 'don't know' responses were recoded as missing. The lowest socioeconomic category was used as the reference category.

For objective 2, maternal ethnicity was grouped into the largest ethnic groups within the cohort: White British or Irish, Pakistani heritage, White Other (containing Polish, Slovakian, Romanian, Czech, Other White, Gypsy/Roma/Irish Traveller), Other South Asian (Indian & Bangladeshi), and Other (Chinese, African, Caribbean, Mixed, or Other). For objective 3, ethnic categories were further collapsed as the overlap between

Confidential: For Review Only

socioeconomic categories and ethnicity were too small for model estimation. Hence the groups were White British, South Asian (Pakistani, Indian & Bangladeshi) and Other. White British were the reference group.

There were three binary covariates: (1) Maternal country of birth was analysed as Not born in the UK/Born in the UK, (2) Maternal first spoken language was analysed as Not English/English, and (3) Child sex was analysed as Female/Male. There was one continuous covariate, (4) child age in months at the date of the most recent ASQ.

Outcome measure (Child development via Ages and Stages Questionnaire (ASQ))

All parents living in England receive a visit from their health visitor when their child turns 2-years-old, during which children's development is assessed using the 24, 27, or 30 month Ages and Stages Questionnaire (ASQ). ASQ-3 was developed to screen for developmental delay and comprises 21 age specific questionnaires for children aged between 1 month and 66 months [34]. ASQ-3 covers five key domains of developmental status of a child's following areas [35]: Communication - babbling, vocalizing, listening, and understanding; Gross Motor: arm, body, and leg movements; Fine Motor: hand and finger movements; Problem Solving: learning and playing with toys; and Personal-Social: solitary social play and play with toys and other children.

There are three response options to each question (yes/sometimes/not yet, with scores being yes=10, sometimes=5 and not yet=0). The score of each domain ranges between 0 and 60, however each age version and domain of the ASQ has different cut-offs to categorise children into; (1) 'below cut-off', where a child requires further assessment and intervention with a professional, (2) 'monitor', where a child's development should be monitored, and (3) 'above cut off', where a child's development is on schedule. For objective 2, in line with the Public Health England method for calculating whether children have a Good Level of Development (GLD), an overall ASQ score was created by categorising as 'Not at risk'=above cut off and/or monitor on all 5 domains, and 'At risk'='below cut off' on 1 or more domains (see https://fingertips.phe.org.uk/static-reports/health-trends-in-england/England/best_start_in_life.html; [25]).

For objective 3, to create an outcome that was more sensitive to identifying children *potentially* at risk, an additional ASQ 'Full' GLD (FGLD) score was created by categorising as 'Not at risk'='above cut off' on all 5 domains and 'At risk'='below cut off' AND/OR 'monitor' on 1 or more domains. Scores were only calculated if all 5 domains were available.

Analysis

For objective 1 we described the number of children at risk of poor development overall. For reference we compare proportions of children at risk to Jung et al. (2025), who described child developmental outcomes in the ASQ-3 across England.

For objectives 2 and 3, a Directed Acyclic Graph (DAG) ascertained the adjustment set for the effect of each socioeconomic indicator (maternal education, financial management, social ladder, number of people to count on). Socioeconomic variables could be either mediators or confounders of specific associations depending on the relationship of interest, hence each relationship of interest required a separate adjustment set and analysis [36]. Justification for inclusion of each confounder/covariate was based on prior literature [10,24]. We did not include area deprivation and/or area as a covariate, since the areas under study are relatively homogeneous in terms of deprivation [30].

- *Figure 1 placed here*

Confidential: For Review Only

-

Figure 1. Directed Acyclic Graph with estimates of interest labelled 1-4.

Model (#) and key predictor	Confounders included in model	Covariates included in model
1. Maternal education	Migrant to UK, English is first language, Ethnic group	Child age, child sex
2. Financial management	Maternal education, Migrant to UK, English is first language, Ethnic group	Child age, child sex
3. Social status (social ladder)	Maternal education, Migrant to UK, English is first language, Ethnic group	Child age, child sex
4. Social support (people to count on)	Maternal education, Migrant to UK, English is first language, Ethnic group	Child age, child sex

Table 1. Model number with key predictor, and covariates included in each model

For objective 1, the prevalence of children at risk of poor development was described using the GLD method. For objectives 2-3, binary logistic regression models were conducted to produce odds ratios and 95% confidence intervals using the FGLD method, and model-based estimates (ie. adjusted predictions) for the outcomes were presented using plots.

For objective 3, to investigate if socioeconomic differences in child development varied by ethnic group, we estimated four additional binary logistic models with ethnicity (categorised as White British, South Asian, and Other) and each socioeconomic predictor (maternal education, financial management, social ladder, and people to count on), with an interaction fitted between these variables. We applied the specified adjustment sets for each predictor using Table 1. A likelihood ratio test was performed to compare the fit of the models with and without the interaction by ethnic group, where the fit of the two competing models was compared and the statistic represents a direct comparison of the relative likelihood of the data, given the best fit of the two models [37].

Results were considered to be statistically significant if $p<.05$. R was used for all analysis, key packages included *tidyverse, ggplot2, ggdist*, and *ggeffects*.

Missing data

We applied complete case analysis, as this gives unbiased results when the chance of being a complete case does not depend on the outcome after taking the covariates into consideration [38]. The analysis therefore assumes that having complete data does not depend on a child's development, after taking into account the included predictor and control variables.

Community involvement

Members of the community are involved in the design and conduct of BiBBS cohort research, including recruitment, questionnaires, measures and interpretation and dissemination of findings, through regular Community Research Advisory Group meetings, composed of Better Start Bradford residents.

Confidential: For Review Only

RESULTS

Sample

- *Figure placed here*

Figure 2. Eligible participants and numbers included in models for each objective

Note: *The sample contained 2887 pregnancies with a 2-year visit recorded and 72% of these had an ASQ record. It is beyond the scope of this study to explore reasons behind non-completion of the ASQ, however, a mixed methods study by our team will explore this further (Henderson et al., in preparation).*

Figure 2 shows the number of pregnancies with ASQ records, and the sample sizes for each objective and model.

Objective 1

Objective 1 was to estimate the number of children at risk of poor child development overall, and the number of children at risk of poor development in each domain. Within the 2,003 individual children with linked ASQs, 445 (22%) of all children were at risk of overall poor development using the GLD method, and 884 (44%) were at risk using the FGLD method.

Objective 2

Objective 2 was to investigate the association between individual indicators of socioeconomic position and early child development. The FGLD method was used as the outcome, which is more sensitive to identifying children potentially at risk of poor development (44%). Descriptives for all participants are presented in Supplementary Material B, and logistic regression models are presented in Supplementary Material C.

Odds ratios from adjusted logistic regressions between the predictor variables and achieving the expected level of development show that having a degree (OR=2.09, 95% CI 1.33 to 3.30), living comfortably (OR=1.90, 95% CI 1.08 to 3.36), and social status (OR=1.13, 95%CI 1.03 to 1.24) all resulted in increased odds of having a FGLD. Social support was not associated with having a FGLD (OR=1.01, 95% CI 0.95 to 1.07).

- *Figures 3a-3d placed here as one –*

Figure 3a-3d. Adjusted predictions for models 1-4, with no interaction for ethnicity included.

Note: the overlap of 95% CIs in Figures a and b does not mean that the difference between any given pair of estimates is not significant at p<.05.

All socioeconomic predictors, except for social support, were significantly associated with the probability of expected development, and the figures visualise the socioeconomic gradients. The adjusted predictions reveal that the socioeconomic gradient is steepest by social status, with the largest difference between the highest and lowest socioeconomic category for this predictor.

Objective 3

Objective 3 was to investigate if the association between individual indicators of socioeconomic position and early child development varies by ethnic group. Results from the likelihood ratio tests indicated that the model with the interaction fitted the data equally well for maternal education (X^2=7.52, p=0.275) and financial security

Confidential: For Review Only

(X^2=2.56, p=0.632), whereas the model with the interaction was superior for social status (X^2=2.67, p<0.001) and social support (X^2=3.92, p<0.001). The adjusted predictions (see below) indicate that social status and support were less strongly related to child development for South Asian groups, in comparison to White British and Other ethnic groups. Full model results are in Supplementary File D, and adjusted predictions from each model are presented below. The sensitivity analysis including social status and support as categorical predictors showed a similar pattern of results.

- *Figures 4a-4d placed here as one -*

Figure 4a-4d. Adjusted predictions for models 1-4, with interaction by ethnicity included.

Confidential: For Review Only

DISCUSSION

Our analysis of longitudinal, routinely collected data from this place-based birth cohort study reveals a concerningly high proportion of children in inner-city Bradford identified as being at risk of poor development by age two (22%). The prevalence of children at risk is considerably higher than a previously estimated average for England (14%) [25], underscoring the significant vulnerability of this specific population. This high rate of developmental risk will likely have long-lasting implications for these children's future educational attainment, health, and overall wellbeing [6].

Whilst maternal education and financial security had previously been established as important predictors of children's outcomes [7,16], our study shows the additional relevance of parent-reported social status. Social status presented the steepest social gradient in children's outcomes in comparison to maternal education (18%), financial security (15%), and social support (2%), with a 26% percentage point gap between the highest and lowest points on the social status scale. Whilst research linking social status to early developmental outcomes is limited, this aligns with broader research indicating that subjective socioeconomic measures may more strongly predict birth outcomes in deprived populations, when compared to parental education [16]. This suggests that a self-reported, subjective assessment of social standing offers a more nuanced understanding of socioeconomic position within disadvantaged populations, potentially revealing how parents in deprived areas might mitigate the challenges of socioeconomic deprivation through their own perceived social status. The lack of a significant association between social support and child outcomes contrasts with previous research that has indicated a positive relationship [21,22], however, further analysis revealed that this may be due to differences by ethnic group.

Whilst the socioeconomic gradients in maternal education and financial management appeared broadly consistent across White British, South Asian, and Other ethnic groups, a more nuanced picture emerged regarding the role of social status and support. Social support became a significant predictor of child development when we allowed the effect to vary by ethnic group. The adjusted predictions (Figures 4a-4d) revealed a weaker relationship between social status and support and child outcomes specifically within the South Asian group, (in comparison to the White British and Other ethnic groups). This suggests that the null result in the original social support model was perhaps driven by the weak association for South Asian groups, suggesting social support may be a protective factor for many families. This aligns with existing research that has reported a lack of expected socioeconomic gradients for maternal and child outcomes among ethnic minority groups [16,26–28], though these previous studies used different socioeconomic indicators. Our study extends this by demonstrating that a subjective measure of social support may have different relationships with children's outcomes depending on ethnic group.

This finding may reflect unmeasured confounding or inaccurate measurement. For instance, our measure of social support might not capture the *quality* or *type* of support received, which could be more influential than the quantity of social connections. Given that South Asian families in this area are often characterised by close proximity to family members living in the same households [40], the nature and dynamics of this support might differ significantly from other groups. Ultimately, the relationship between ethnicity and children's outcomes is of course influenced by a multitude of factors beyond socioeconomic position, such as cultural practices, prejudice and racism [29]. Early years practitioners working with families should be mindful of potential cultural nuances in how social factors influence

Confidential: For Review Only

child development within different communities. To gain a more comprehensive understanding of the true impact of socioeconomic position across diverse ethnic groups, future research should continue to employ larger samples of ethnic minorities and apply a wider array of socioeconomic measures.

Strengths and limitations

Findings from BiBBS may generalise to many similar urban populations, especially those with high ethnic diversity and socioeconomic deprivation. However, the disadvantaged population under study may be relatively homogeneous in terms of socioeconomic characteristics, reducing the potential for observing significant social gradients. It was not possible to include income as a predictor in this study, as this was not included in the baseline questionnaire due to low acceptability in this population. Whilst we tentatively suggest that the associations observed here might be causal and the longitudinal design of the study facilitates this, the study is observational and hence we cannot rule out the presence of unmeasured confounders which could be driving the associations that we present.

Conclusion and implications

A high proportion of children do not meet expected levels of development in this ethnically diverse, socioeconomically disadvantaged population. Higher socioeconomic position may protect against poor early development in such communities, and the mechanism of this may differ by ethnic group. The high prevalence of children at risk can be partly explained by socioeconomic position, and this suggests a continued need for broader public health initiatives, e.g. proportionate universal approaches that encompass both universal prevention and early targeted interventions [6]. This necessitates investment in early years practitioners, such as health visitors, who can work directly in these communities to identify children at risk, and connect them with relevant support services.

Confidential: For Review Only

0.7

REQUIRED STATEMENTS

Acknowledgements

The integration of research and practice in Bradford has only been possible because of the enthusiasm and commitment of staff and volunteers across children's services in Bradford. We are grateful to all Born in Bradford staff, the Better Start Bradford partnership and staff, all Better Start Bradford project teams, health professionals, local authority and voluntary and community sector organisations who have supported the integration of research into practice. We are grateful to all the families taking part in BiBBS and all members of the Community Research Advisory Group. A poster regarding this study was presented at the International Network for Research Inequalities in Child Health (INRICH) workshop in 2025.

Funding statement

This study has received funding from the National Lottery Community Fund (previously the Big Lottery Fund) as part of the A Better Start programme (Ref 10094849). The funder was not involved in the design of the study and collection, analysis, and interpretation of data nor in writing the manuscript.

SLB, JD, and KP are also supported by the NIHR Yorkshire and Humber Applied Research Collaboration (ARC-YH; Ref: NIHR200166, see https://www.arc-yh.nihr.ac.uk). The views in this publication are those of the authors and not necessarily those of the NIHR or the Department of Health and Social Care.

Competing interests

The authors have no competing interests to declare.

Authors contributions

KEM conducted the analysis and drafted the manuscript. MW linked the BiBBS baseline with the health visiting datasets. KEP, SLB, JL and JD all reviewed the manuscript and provided substantial contributions.

Ethical approval

The protocol for BiBBS recruitment and collection of routine outcome data was approved by Bradford Leeds NHS Research Ethics Committee (15/YH/0455). Research governance approval was gained from Bradford Teaching Hospitals NHS Foundation Trust.

Data availability

Data cannot be shared publicly as they are available through a system of managed open access. Researchers interested in accessing the data can find details and procedures on the Born in Bradford website (https://borninbradford.nhs.uk/research/how-to-access-data/). Data access is subject to review by the Born in Bradford Executive, who review proposals on a monthly basis. Requests can be submitted to borninbradford@bthft.nhs.uk. Data sharing agreements are established between the researcher and the Bradford Institute for Health Research. *Analysis code and results will be made publicly available in OSF upon acceptance of the paper (file has been uploaded for Editorial review).*

Confidential: For Review Only

REFERENCES

1 Tierney AL, Nelson CA. Brain Development and the Role of Experience in the Early Years. *Zero Three*. 2009;30:9.

2 Newman L, Judd F, Olsson CA, *et al.* Early origins of mental disorder - risk factors in the perinatal and infant period. *BMC Psychiatry*. 2016;16:1–8. doi: 10.1186/s12888-016-0982-7

3 Murrah WM, III. Comparing Self-Regulatory and Early Academic Skills as Predictors of Later Math, Reading, and Science Elementary School Achievement. *ProQuest LLC*. 2010;102.

4 Amin V, Behrman JR, Spector TD. Does more schooling improve health outcomes and health related behaviors? Evidence from U.K. twins. *Econ Educ Rev*. 2013;35:134–48. doi: 10.1016/j.econedurev.2013.04.004

5 Black M, Akanni L, Adjei NK, *et al.* Impact of child socioemotional and cognitive development on exam results in adolescence: findings from the UK Millennium Cohort Study. *Arch Dis Child*. 2025;0:1–6. doi: 10.1136/ARCHDISCHILD-2024-327963

6 Marmot M, Allen J, Goldblatt P, *et al.* The Marmot review: Fair society, healthy lives. *The Strategic Review of Health Inequalities in England Post-2010*. 2010.

7 Pillas D, Marmot M, Naicker K, *et al.* Social inequalities in early childhood health and development: a European-wide systematic review. *Pediatric Research 2014 76:5*. 2014;76:418–24. doi: 10.1038/pr.2014.122

8 Becker B. Social disparities in children's vocabulary in early childhood. Does pre-school education help to close the gap?1. *Br J Sociol*. 2011;62:69–88. doi: 10.1111/J.1468-4446.2010.01345.X

9 Flouri E, Tzavidis N, Kallis C. Area and family effects on the psychopathology of the Millennium Cohort Study children and their older siblings. *Journal of Child Psychology and Psychiatry*. 2010;51:152–61. doi: 10.1111/J.1469-7610.2009.02156.X

10 Kelly Y, Sacker A, Del Bono E, *et al.* What role for the home learning environment and parenting in reducing the socioeconomic gradient in child development? Findings from the Millennium Cohort Study. *Arch Dis Child*. 2011;96:832–7. doi: 10.1136/adc.2010.195917

11 Lai ETC, Wickham S, Law C, *et al.* Poverty dynamics and health in late childhood in the UK: evidence from the Millennium Cohort Study. *Arch Dis Child*. 2019;archdischild-2018-316702. doi: 10.1136/archdischild-2018-316702

12 Duncan GJ, Magnuson K. Socioeconomic status and cognitive functioning: moving from correlation to causation. *Wiley Interdiscip Rev Cogn Sci*. 2012;3:377–86. doi: 10.1002/wcs.1176

13 Carneiro P, Cattan S, Neves H. Theoretical and empirical perspectives on the link between poverty, parenting and children's outcomes. *Fisc Stud*. 2025;46:9–35. doi: 10.1111/1475-5890.12404

Confidential: For Review Only

14 Galobardes B, Shaw M, Lawlor DA, *et al.* Indicators of socioeconomic position (part 1). *J Epidemiol Community Health (1978)*. 2006;60:7–12. doi: 10.1136/jech.2004.023531

15 Ursache A, Noble KG. Neurocognitive development in socioeconomic context: Multiple mechanisms and implications for measuring socioeconomic status. *Psychophysiology*. 2016;53:71–82. doi: 10.1111/psyp.12547

16 Uphoff EP, Pickett KE, Wright J. Social gradients in health for Pakistani and White British women and infants in two UK birth cohorts. *Ethn Health*. 2016;21:452–67. doi: 10.1080/13557858.2015.1091442

17 Präg P. Subjective socio-economic status predicts self-rated health irrespective of objective family socio-economic background. *Scand J Public Health*. 2020;48:707–14. doi: 10.1177/1403494820926053/SUPPL_FILE/SJP926053_SUPPLEMENTAL_MATERIAL.PDF

18 Moss RH, Kelly B, Bird PK, *et al.* Turning their backs on the 'ladder of success'? Unexpected responses to the MacArthur Scale of Subjective Social Status. *Wellcome Open Res*. 2023;8:11. doi: 10.12688/WELLCOMEOPENRES.18655.2

19 Heitz HK, Mitchell AM. Subjective Social Status and Mental Health Outcomes: The Role of Mindfulness. *J Prev Health Promot*. 2022;3:445–67. doi: 10.1177/26320770221087248

20 Wickramaratne PJ, Yangchen T, Lepow L, *et al.* Social connectedness as a determinant of mental health: A scoping review. *PLoS One*. 2022;17:e0275004. doi: 10.1371/JOURNAL.PONE.0275004

21 Shin EK, Lewinn K, Bush N, *et al.* Association of Maternal Social Relationships With Cognitive Development in Early Childhood. *JAMA Netw Open*. 2019;2:e186963–e186963. doi: 10.1001/JAMANETWORKOPEN.2018.6963

22 Imanishi Y, Ikehara S, Aochi Y, *et al.* The association between maternal social support levels during pregnancy and child development at three years of age: the Japan Environment and Children's Study. *Environ Health Prev Med*. 2024;29:18–18. doi: 10.1265/EHPM.23-00211

23 Chattoo S, Atkin K. *Understanding 'race' and ethnicity 2e: Theory, history, policy, practice.* 2nd ed. Policy Press 2019.

24 Cattan S, Fitzsimons E, Goodman A, *et al.* Early childhood inequalities. *Oxford Open Economics*. 2024;3:i711–40. doi: 10.1093/OOEC/ODAD072

25 Jung J, Cattan S, Powell C, *et al.* Early child development in England: cross-sectional analysis of ASQ®-3 records from the 2-2½-year universal health visiting review using national administrative data (Community Service Dataset, CSDS). *Int J Popul Data Sci*. 2025;9:2459. doi: 10.23889/IJPDS.V9I2.2459

26 Mallicoat B, P Uphoff E, E Pickett K. Estimating Social Gradients in Health for UK Mothers and Infants of Pakistani Origin: Do Latent Class Measures of Socioeconomic Position Help? *J Immigr Minor Health*. Published Online First: 2020. doi: 10.1007/s10903-020-00977-9

Confidential: For Review Only

27 Kuo YL, Casillas A, Walton KE, *et al.* The intersectionality of race/ethnicity and socioeconomic status on social and emotional skills. *J Res Pers*. 2020;84:103905. doi: 10.1016/j.jrp.2019.103905

28 Mooney KE, Pickett KE, Shire K, *et al.* Socioeconomic disadvantage and ethnicity are associated with large differences in children's working memory ability: analysis of a prospective birth cohort study following 13,500 children. *BMC Psychol*. 2022;10:67. doi: 10.1186/s40359-022-00773-0

29 Marks AK, Garcia Coll C. Education and developmental competencies of ethnic minority children: Recent theoretical and methodological advances. *Developmental Review*. 2018;50:90–8. doi: 10.1016/j.dr.2018.05.004

30 Dickerson J, Bridges S, Willan K, *et al.* Born in Bradford's Better Start (BiBBS) interventional birth cohort study: Interim cohort profile. *Wellcome Open Research 2022 7:244*. 2022;7:244. doi: 10.12688/wellcomeopenres.18394.1

31 Ghaferi AA, Schwartz TA, Pawlik TM. STROBE Reporting Guidelines for Observational Studies. *JAMA Surg*. 2021;156:577–8. doi: 10.1001/JAMASURG.2021.0528

32 Bradford Council. Poverty in Bradford District | Understanding Bradford District. 2019. https://ubd.bradford.gov.uk/about-us/poverty-in-bradford-district/ (accessed 1 February 2021)

33 Dickerson J, Bird PK, McEachan RRC, *et al.* Born in Bradford's Better Start: An experimental birth cohort study to evaluate the impact of early life interventions. *BMC Public Health*. 2016;16:711. doi: 10.1186/s12889-016-3318-0

34 Velikonja T, Edbrooke-Childs J, Calderon A, *et al.* The psychometric properties of the Ages & Stages Questionnaires for ages 2-2.5: a systematic review. *Child Care Health Dev*. 2017;43:1–17. doi: 10.1111/CCH.12397

35 Squires J, Bricker D. Ages & Stages Questionnaires [R], (ASQ-3 [TM]): A Parent-Completed Child-Monitoring System. *Brookes Publishing Company*. 2009.

36 Shrier I, Platt RW. Reducing bias through directed acyclic graphs. BMC Med Res Methodol. 2008;8:1–15.

37 Glover S, Dixon P. Likelihood ratios: A simple and flexible statistic for empirical psychologists. *Psychon Bull Rev*. 2004;11:791–806. doi: 10.3758/BF03196706/METRICS

38 Hughes RA, Heron J, Sterne JAC, *et al.* Accounting for missing data in statistical analyses: multiple imputation is not always the answer. *Int J Epidemiol*. 2019;48:1294–304. doi: 10.1093/IJE/DYZ032

39 Westreich D, Greenland S. The Table 2 Fallacy: Presenting and Interpreting Confounder and Modifier Coefficients. *Am J Epidemiol*. 2013;177:292–8. doi: 10.1093/AJE/KWS412

40 Thapar-Bjorkert S, Sanghera G. Social capital, educational aspirations and young Pakistani Muslim men and women in Bradford, West Yorkshire. 2010.

Confidential: For Review Only

FIGURE LEGENDS

Figure 1. Directed Acyclic Graph with estimates of interest labelled 1-4.

Figure 2. Eligible participants and numbers included in models for each objective

Figure 3a-3d. Adjusted predictions for models 1-4, with no interaction for ethnicity included.

Figure 4a-4d. Adjusted predictions for models 1-4, with interaction by ethnicity included.

Confidential: For Review Only

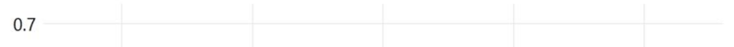

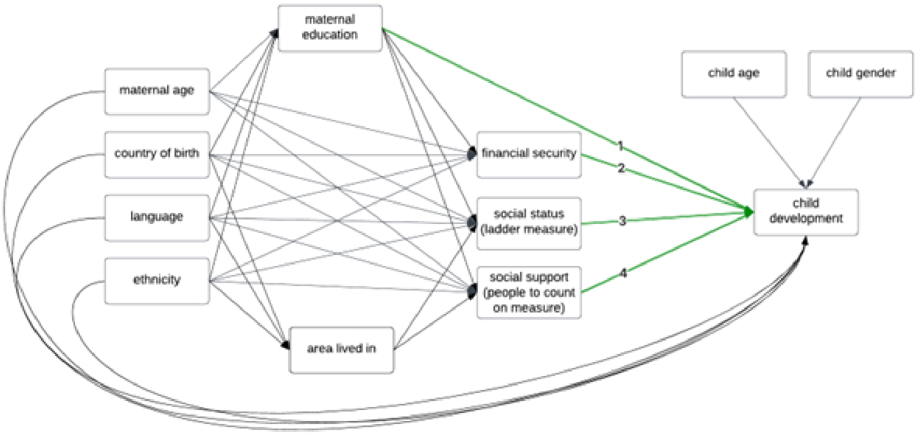

Figure 1. Directed Acyclic Graph with estimates of interest labelled 1-4.

429x195mm (38 x 38 DPI)

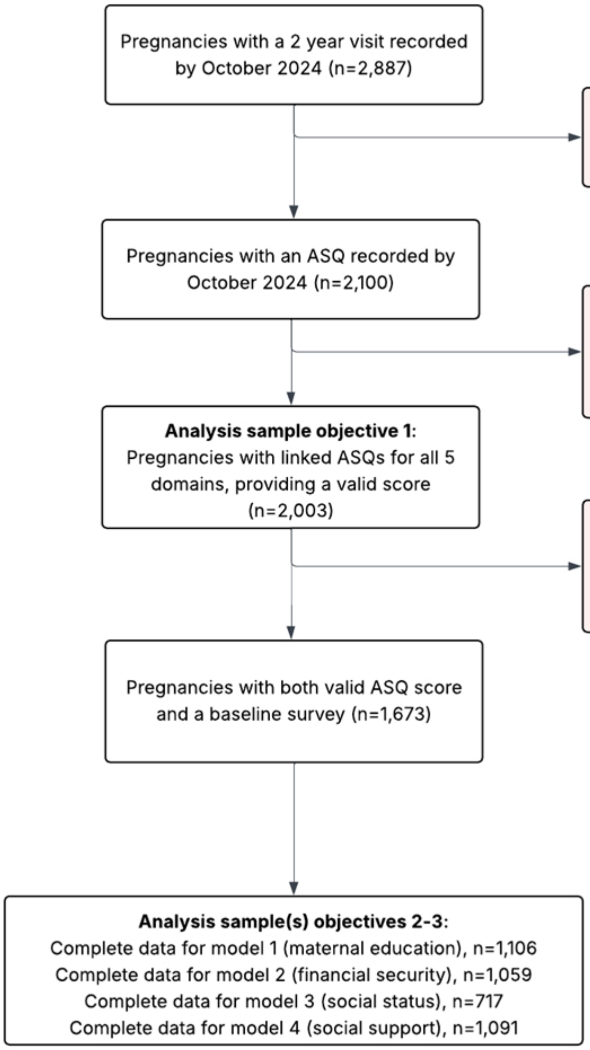

Figure 2. Eligible participants and numbers included in models for each objective

357x409mm (63 x 63 DPI)

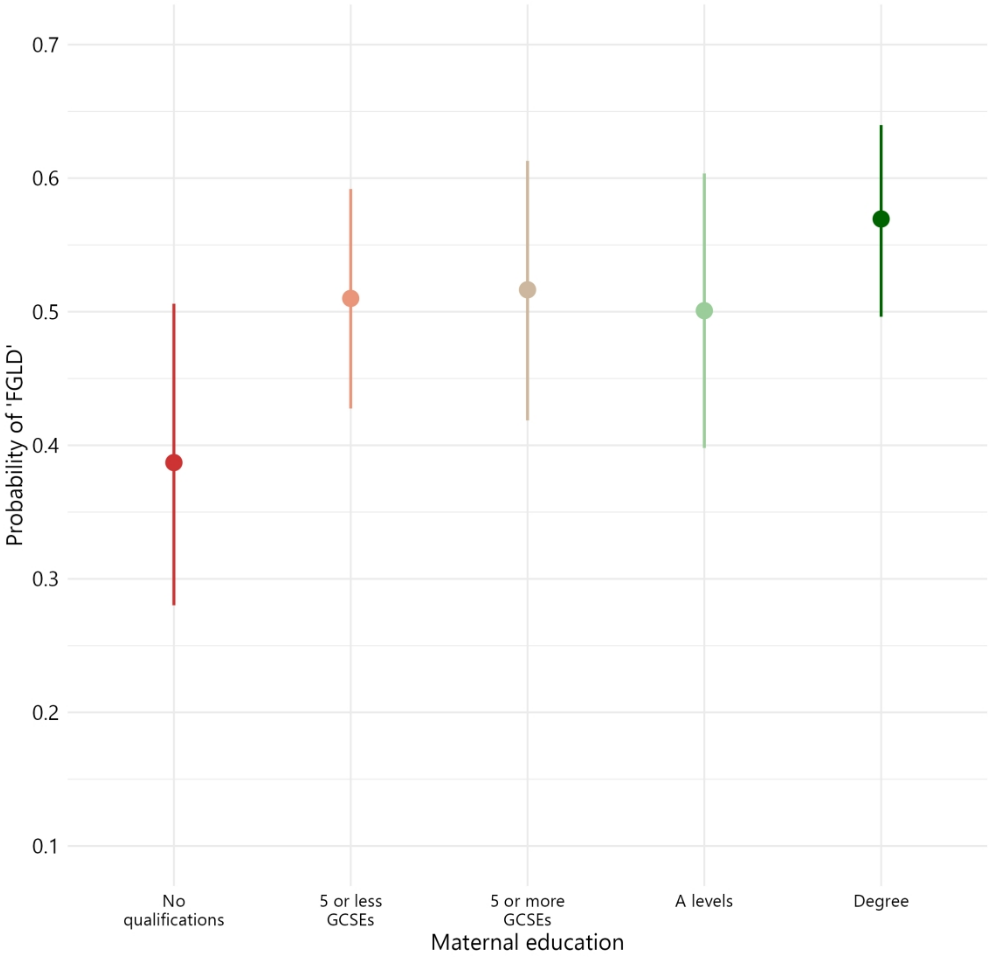

Figure 3a-3d. Adjusted predictions for models 1-4, with no interaction for ethnicity included.

516x516mm (118 x 118 DPI)

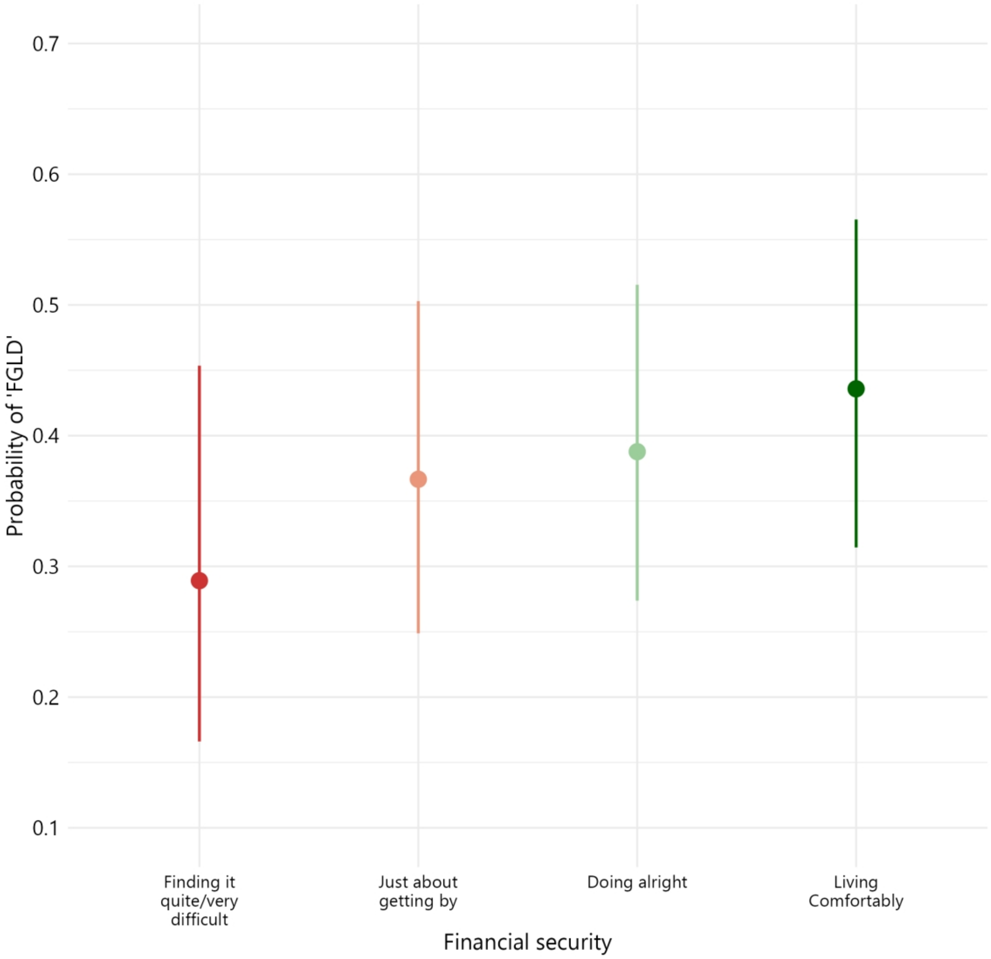

Figure 3a-3d. Adjusted predictions for models 1-4, with no interaction for ethnicity included.

516x516mm (118 x 118 DPI)

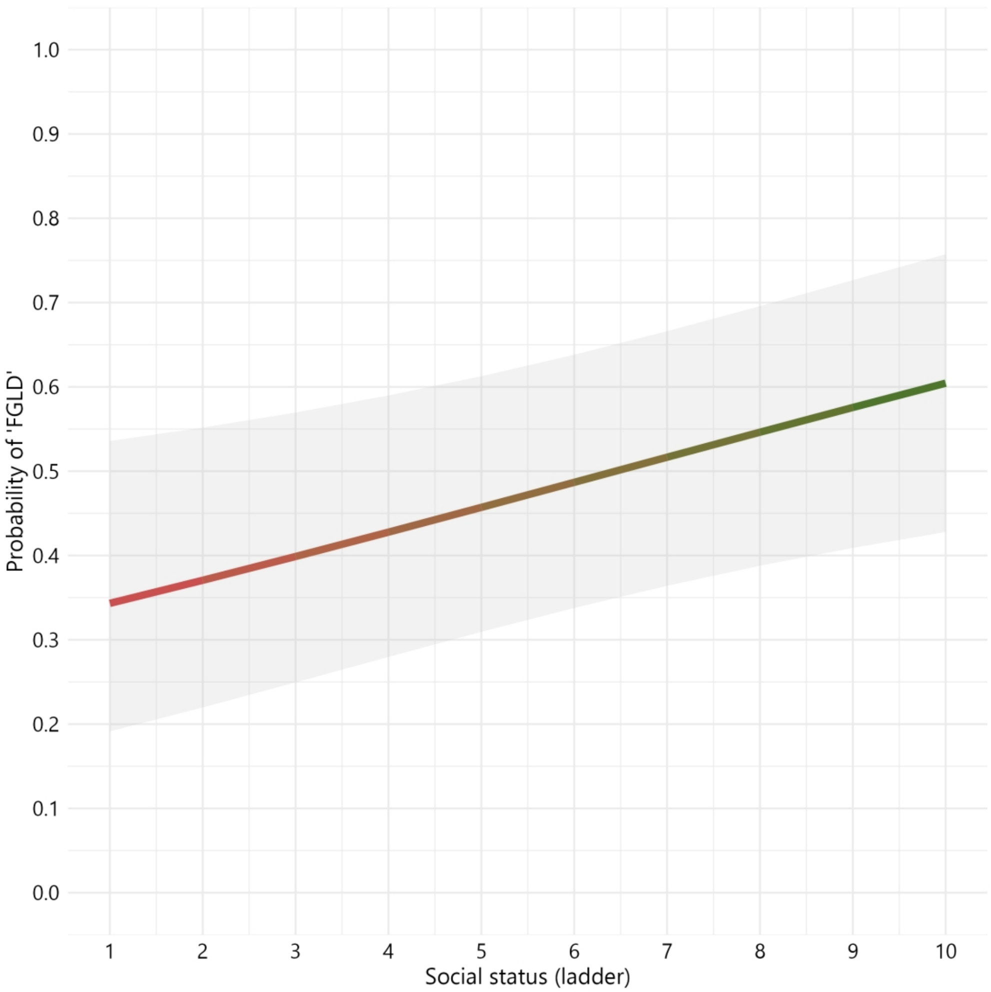

Figure 3a-3d. Adjusted predictions for models 1-4, with no interaction for ethnicity included.

516x516mm (118 x 118 DPI)

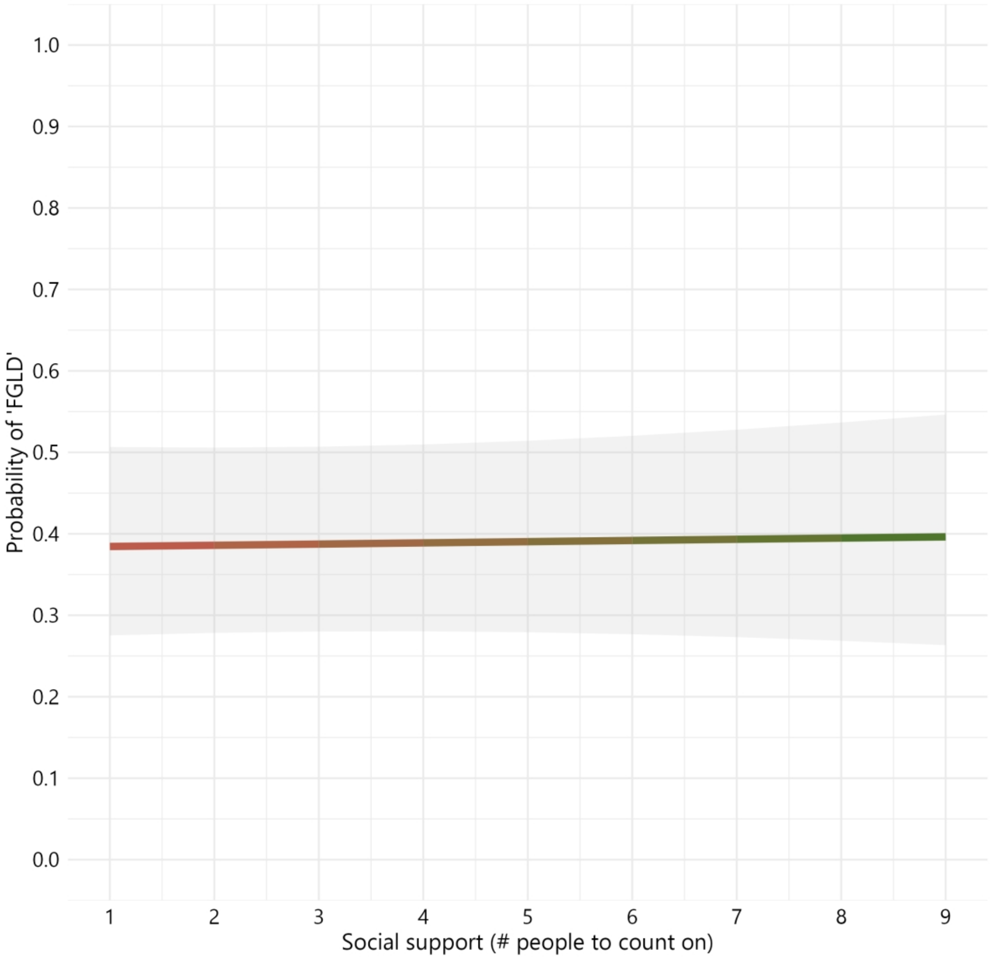

Figure 3a-3d. Adjusted predictions for models 1-4, with no interaction for ethnicity included.

516x516mm (118 x 118 DPI)

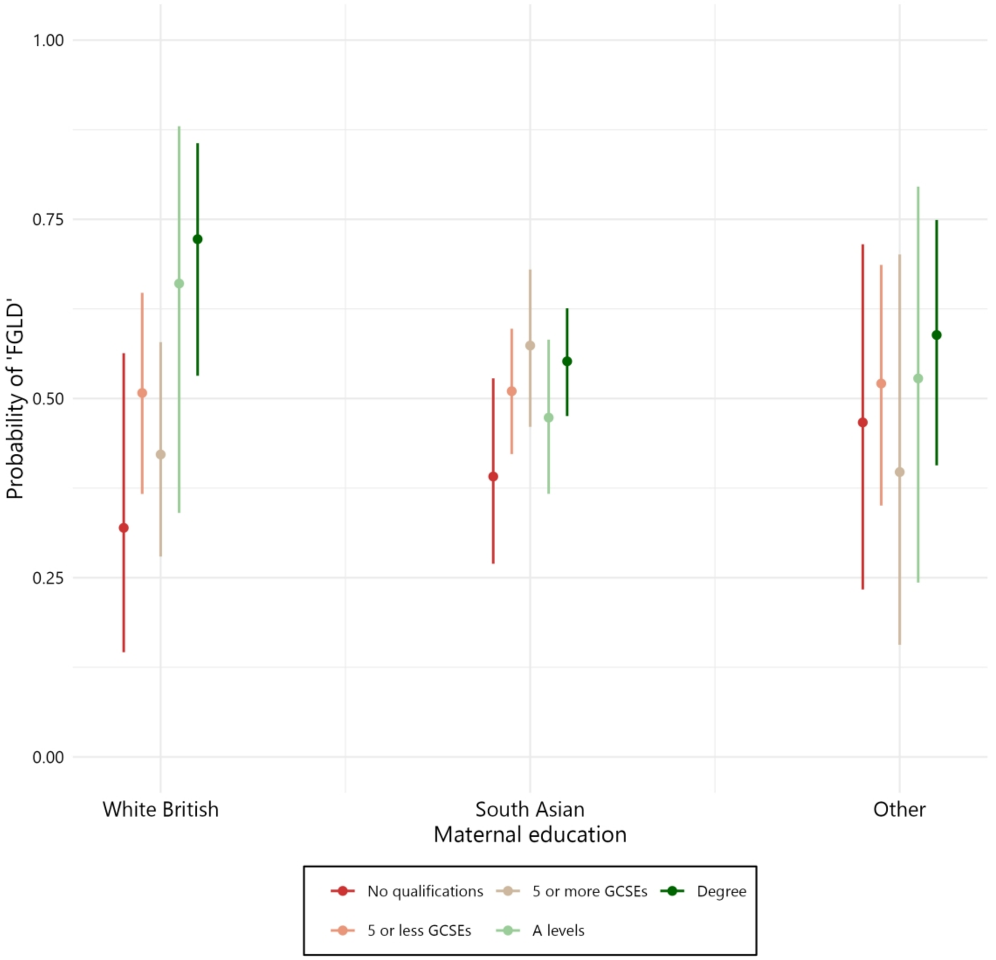

Figure 4a-4d. Adjusted predictions for models 1-4, with interaction by ethnicity included.

516x516mm (118 x 118 DPI)

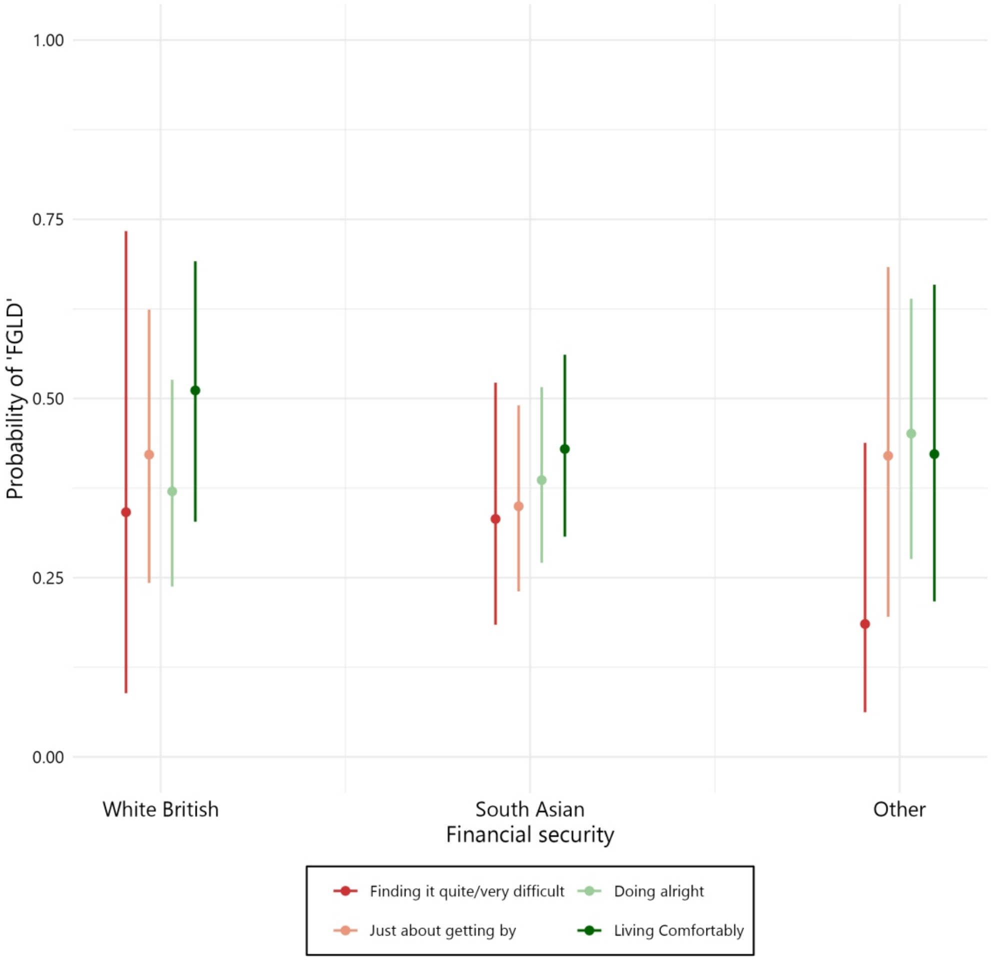

Figure 4a-4d. Adjusted predictions for models 1-4, with interaction by ethnicity included.

516x516mm (118 x 118 DPI)

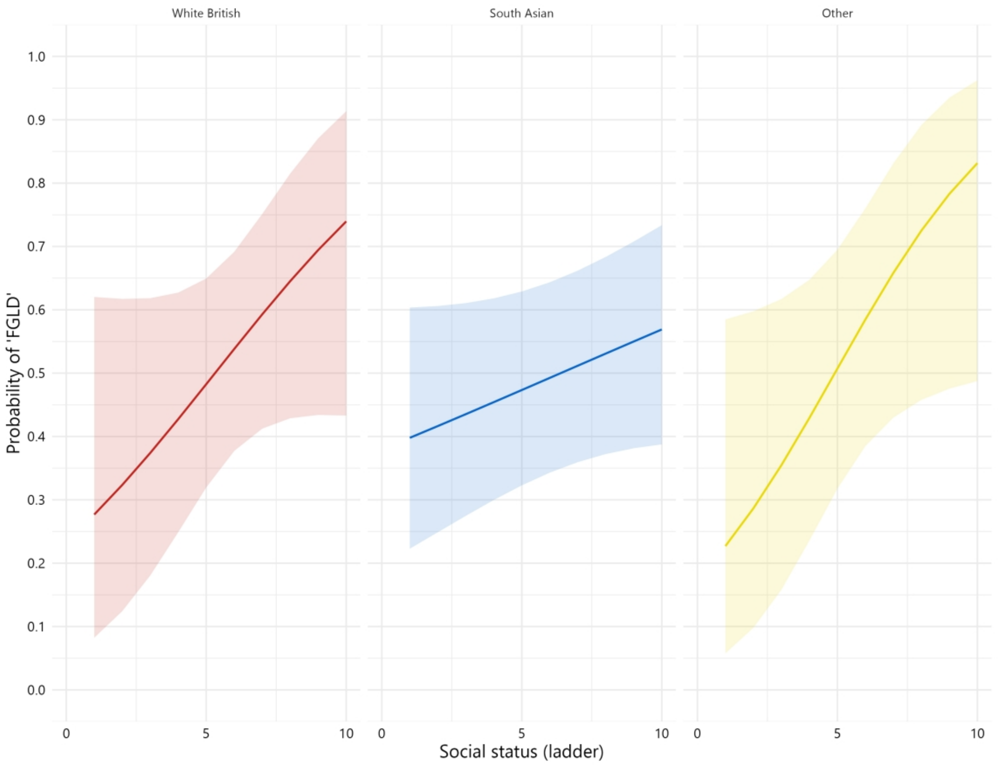

Figure 4a-4d. Adjusted predictions for models 1-4, with interaction by ethnicity included.

645x516mm (118 x 118 DPI)

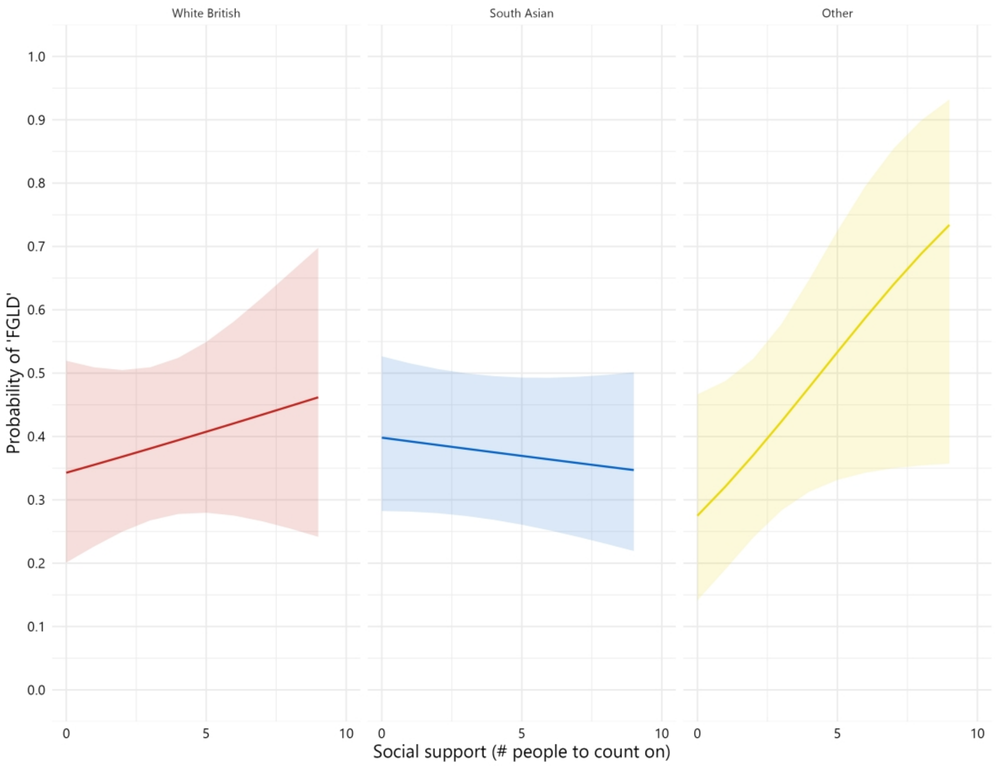

Figure 4a-4d. Adjusted predictions for models 1-4, with interaction by ethnicity included.

645x516mm (118 x 118 DPI)

STROBE Statement—Checklist of items that should be included in reports of ***cohort studies***

	Item No	Recommendation	Page No
**Title and abstract**	1	(*a*) Indicate the study's design with a commonly used term in the title or the abstract	1
		(*b*) Provide in the abstract an informative and balanced summary of what was done and what was found	1
**Introduction**			
Background/rationale	2	Explain the scientific background and rationale for the investigation being reported	3-4
Objectives	3	State specific objectives, including any prespecified hypotheses	5
**Methods**			
Study design	4	Present key elements of study design early in the paper	6
Setting	5	Describe the setting, locations, and relevant dates, including periods of recruitment, exposure, follow-up, and data collection	6-7
Participants	6	(*a*) Give the eligibility criteria, and the sources and methods of selection of participants. Describe methods of follow-up	6-7
		(*b*) For matched studies, give matching criteria and number of exposed and unexposed	NA
Variables	7	Clearly define all outcomes, exposures, predictors, potential confounders, and effect modifiers. Give diagnostic criteria, if applicable	6-8
Data sources/ measurement	8*	For each variable of interest, give sources of data and details of methods of assessment (measurement). Describe comparability of assessment methods if there is more than one group	6
Bias	9	Describe any efforts to address potential sources of bias	7-8
Study size	10	Explain how the study size was arrived at	10
Quantitative variables	11	Explain how quantitative variables were handled in the analyses. If applicable, describe which groupings were chosen and why	8
Statistical methods	12	(*a*) Describe all statistical methods, including those used to control for confounding	7-8
		(*b*) Describe any methods used to examine subgroups and interactions	7-8
		(*c*) Explain how missing data were addressed	9
		(*d*) If applicable, explain how loss to follow-up was addressed	NA
		(*e*) Describe any sensitivity analyses	NA
**Results**			
Participants	13*	(a) Report numbers of individuals at each stage of study—eg numbers potentially eligible, examined for eligibility, confirmed eligible, included in the study, completing follow-up, and analysed	10
		(b) Give reasons for non-participation at each stage	10
		(c) Consider use of a flow diagram	10
Descriptive data	14*	(a) Give characteristics of study participants (eg demographic, clinical, social) and information on exposures and potential confounders	11
		(b) Indicate number of participants with missing data for each variable of interest	11
		(c) Summarise follow-up time (eg, average and total amount)	11

Confidential: For Review Only

Outcome data	15*	Report numbers of outcome events or summary measures over time	10
Main results	16	(a) Give unadjusted estimates and, if applicable, confounder-adjusted estimates and their precision (eg, 95% confidence interval). Make clear which confounders were adjusted for and why they were included	No unadjusted estimates, 12
		(b) Report category boundaries when continuous variables were categorized	6
		(c) If relevant, consider translating estimates of relative risk into absolute risk for a meaningful time period	NA
Other analyses	17	Report other analyses done—eg analyses of subgroups and interactions, and sensitivity analyses	NA
**Discussion**			
Key results	18	Summarise key results with reference to study objectives	17
Limitations	19	Discuss limitations of the study, taking into account sources of potential bias or imprecision. Discuss both direction and magnitude of any potential bias	18
Interpretation	20	Give a cautious overall interpretation of results considering objectives, limitations, multiplicity of analyses, results from similar studies, and other relevant evidence	18
Generalisability	21	Discuss the generalisability (external validity) of the study results	18
**Other information**			
Funding	22	Give the source of funding and the role of the funders for the present study and, if applicable, for the original study on which the present article is based	19

*Give information separately for exposed and unexposed groups.

Note: An Explanation and Elaboration article discusses each checklist item and gives methodological background and published examples of transparent reporting. The STROBE checklist is best used in conjunction with this article (freely available on the Web sites of PLoS Medicine at http://www.plosmedicine.org/, Annals of Internal Medicine at http://www.annals.org/, and Epidemiology at http://www.epidem.com/). Information on the STROBE Initiative is available at http://www.strobe-statement.org.

Confidential: For Review Only

Variable	Not at risk (n=1,558, 56%)	At risk (FGLD) (n=884, 44%)	Total sample (n=2,003, 100%)
**Categorical variables N (%)**			
**Maternal education**			
No qualifications	60 (5)	57 (6)	117 (6)
5 or less GCSEs	223 (20)	144 (16)	367 (18)
5 or more GCSEs	86 (8)	64 (7)	150 (7)
A levels	75 (7)	49 (6)	124 (6)
Degree	253 (23)	128 (14)	381 (19)
Missing	*422 (38)*	*442 (5)*	*864 (43)*
**Financial security**			
Finding it quite/very difficult	46 (4)	48 (5)	94 (4)
Just about getting by	132 (12)	115 (5)	247 (12)
Doing alright	368 (33)	290 (33)	658 (33)
Living comfortably	324 (29)	189 (21)	513 (26)
Missing	*249 (22)*	*242 (27)*	*491 (25)*
**Maternal ethnicity**			
White British	96 (6)	77 (8)	173 (9)
Pakistani	581 (52)	398 (45)	979 (49)
South Asian	79 (7)	64 (7)	143 (7)
White Other	41 (4)	22 (2)	63 (3)
Other	91 (8)	60 (7)	151 (8)
Missing	*231 (21)*	*263 (30)*	*494 (25)*
**Maternal county of birth**			
UK	415 (37)	322 (36)	737 (37)
Not UK	476 (43)	304 (34)	780 (39)
Missing	*228 (20)*	*258 (19)*	*486 (24)*
**Maternal language spoken**			
English	514 (46)	354 (40)	868 (43)
Not English	354 (32)	254 (29)	608 (30)
Missing	*251 (22)*	*276 (31)*	*527 (26)*
**Child sex**			
Male	621 (55)	375 (42)	996 (50)

Confidential: For Review Only

Female	498 (45)	509 (58)	1007 (50)
Missing	*0 (0)*	*0 (0)*	*0 (0)*
**Continuous variables M (SD)**			
**Child age (n=2003)**	28 (3.10)	29 (3.78)	28.62 (3.45)
**Social ladder (n=700)**	6.27 (1.79)	6.03 (1.72)	6.17 (1.76)
**People to count on (n=1070)**	3.29 (2.49)	3.27 (2.44)	3.28 (2.47)

Supplementary table. Descriptives for all participants by whether they were categorised as at risk or not, using the FGLD method.

Confidential: For Review Only

Predictor (n in model, R^2)	Odds ratio (95% CI)	*p*
**Maternal education (n=1,106, R^2=0.044)**		
No qualifications (ref)	.	.
5 or less GCSEs	1.65 (1.06 to 2.57)	0.027*
5 or more GCSEs	1.69 (0.99 to 2.88)	0.053
A levels	1.59 (0.90 to 2.80)	0.109
Degree	2.09 (1.33 to 3.30)	0.001*
**Financial security (n=1,059, R^2=0.048)**		
Finding it quite/very difficult (ref)		
Just about getting by	1.42 (0.78 to 2.61)	0.251
Doing alright	1.56 (0.89 to 2.73)	0.120
Living comfortably	1.90 (1.08 to 3.36)	0.026*
**Social status (ladder) (n=717, R^2=0.054)**	1.13 (1.03 to 1.24)	0.013*
**Social support (people to count on) (n=1,091, R^2=0.044)**	1.01 (0.95 to 1.07)	0.836

Supplementary Table. Odds ratios for adjusted logistic regression models, with no interaction for ethnicity included.

Note: *each model contained the relevant adjustment sets as presented in Table 1, though these coefficients are not presented as these coefficients can be misinterpreted (Table 2 Fallacy, see [39]).*

Confidential: For Review Only

Predictor	Odds ratio (95% CI)	p
**Maternal education (n=1106, R^2=0.051)**		
No qualifications (ref)	.	.
5 or less GCSEs	2.20 (0.71 to 7.33)	0.181
5 or more GCSEs	1.55 (0.49 to 5.32)	0.464
A levels	4.14 (0.85 to 25.06)	0.093
Degree	5.54 (1.57 – 21.59)	0.010*
**Financial security (n=1059, R^2=0.050)**		
Finding it quite/very difficult (ref)		
Just about getting by	1.41 (0.23 to 8.77)	0.704
Doing alright	1.13 (0.20 to 6.57)	0.883
Living comfortably	2.02 (0.33 to 12.36)	0.429
**Social status (ladder) (n=717, R^2=0.057)**	1.25 (0.96 to 1.65)	0.107
**Social support (people to count on) (n=1091, R^2=0.044)**	1.06 (0.91 to 1.23)	0.472

Supplementary table. Model results when interaction by ethnicity is included.

Confidential: For Review Only

BMJ Paediatrics Open

Do socioeconomic inequalities contribute to the high prevalence of child developmental risk in an ethnically diverse, socioeconomically disadvantaged population? A Born in Bradford's Better Start (BiBBS) study

Journal:	*BMJ Paediatrics Open*
Manuscript ID	bmjpo-2025-003770.R1
Article Type:	Original research
Date Submitted by the Author:	17-Oct-2025

Complete List of Authors:	Mooney, Kate; University of York, Health Sciences; Bradford Institute for Health Research,<br>Dickerson, Josie; Bradford Institute for Health Research<br>Blower, Sarah Louise; University of York, Health Sciences<br>Walker, Matthew; Bradford Institute for Health Research<br>Lister, Jennie; University of York, Health Sciences<br>Pickett, Kate; University of York, Health Sciences

Keywords:	Child Health, Epidemiology

SCHOLARONE™
Manuscripts

BMJ

I, the Submitting Author has the right to grant and does grant on behalf of all authors of the Work (as defined in the below author licence), an exclusive licence and/or a non-exclusive licence for contributions from authors who are: i) UK Crown employees; ii) where BMJ has agreed a CC-BY licence shall apply, and/or iii) in accordance with the terms applicable for US Federal Government officers or employees acting as part of their official duties; on a worldwide, perpetual, irrevocable, royalty-free basis to BMJ Publishing Group Ltd ("BMJ") its licensees and where the relevant Journal is co-owned by BMJ to the co-owners of the Journal, to publish the Work in this journal and any other BMJ products and to exploit all rights, as set out in our licence.

The Submitting Author accepts and understands that any supply made under these terms is made by BMJ to the Submitting Author unless you are acting as an employee on behalf of your employer or a postgraduate student of an affiliated institution which is paying any applicable article publishing charge ("APC") for Open Access articles. Where the Submitting Author wishes to make the Work available on an Open Access basis (and intends to pay the relevant APC), the terms of reuse of such Open Access shall be governed by a Creative Commons licence – details of these licences and which Creative Commons licence will apply to this Work are set out in our licence referred to above.

Other than as permitted in any relevant BMJ Author's Self Archiving Policies, I confirm this Work has not been accepted for publication elsewhere, is not being considered for publication elsewhere and does not duplicate material already published. I confirm all authors consent to publication of this Work and authorise the granting of this licence.

Confidential: For Review Only

TITLE PAGE

Do socioeconomic inequalities contribute to the high prevalence of child developmental risk in an ethnically diverse, socioeconomically disadvantaged population? A Born in Bradford's Better Start (BiBBS) study

Kate E. Mooney (1,2)*, Josie Dickerson (2), Sarah L. Blower (1), Matthew Walker (2), Jennie Lister (1), and Kate E. Pickett (1).

1. Department of Health Sciences, University of York
2. Bradford Institute for Health Research, Bradford Teaching Hospital Foundation Trust

**=corresponding author*

Confidential: For Review Only

ABSTRACT

Background

Socioeconomic inequalities in child development are pervasive, however, less is known regarding the impacts of socioeconomic factors within and across ethnically diverse, socioeconomically disadvantaged populations. This study (1) describes the prevalence of children at risk of poor overall early child development; (2) investigates the relationship between individual indicators of socioeconomic position and early child development; and (3) investigates if the relationship between indicators of socioeconomic position and early child development varies by ethnic group.

Methods

This study uses data from a prospective birth cohort study, Born in Bradford's Better Start (BiBBS). Child development was measured with the Ages and Stages Questionnaire (ASQ) during routine health visiting at age 2-years-old. Binary logistic regression investigated child development by key maternal socioeconomic indicators; maternal education, financial security, social status (measured via the MacArthur Scale of Subjective Social Status), and social support (measured via number of people to count on).

Results

22% of the 2,003 children with a valid developmental assessment were at risk of poor child development. Mothers who had a degree (OR=2.09, 95% CI 1.33 to 3.30), reported 'living comfortably' in financial security (OR=1.90, 95% CI 1.08 to 3.36), and had higher social status (OR=1.13, 1.03 to 1.24) all had higher odds of their child having a good development. Though socioeconomic gradients in maternal education and financial security were consistent across White British, South Asian, and Other ethnic groups, both social support and social status had weaker relationships with child development for South Asian parents.

Conclusion

A high proportion of children are at risk of poor development in this diverse, socioeconomically disadvantaged population. Higher socioeconomic position may protect against poor early development, and the mechanisms underlying this may differ by ethnicity. The findings underline the need for proportionate universal strategies to improve child development in such communities.

Confidential: For Review Only

What is already known on this topic

- Socioeconomic inequalities in early child development are pervasive
- Whilst traditional socioeconomic indicators such as higher parental education are established predictors of child development, less is known regarding subjective indicators such as social support and subjective social status, particularly within ethnically diverse populations.

What this study adds

- A high proportion (22%) of children in the socioeconomically disadvantaged and ethnically diverse Born in Bradford's Better Start (BiBBS) study population are at risk of poor child development.
- Higher maternal education, financial security, and subjective social status are associated with better child development, with the steepest socioeconomic gradient by subjective social status
- The relationships between social support and subjective social status with child development varied by ethnic group, as these did not relate to child development for South Asian parents.

How this study might affect research, practice or policy

- The BiBBS cohort can be used to further understand patterns of childhood inequalities in socioeconomically deprived, ethnically diverse populations
- Future research should focus on how and why various socioeconomic factors impact child development within socioeconomically deprived, ethnically diverse populations
- The psychological impacts associated with low subjective social status could be addressed through participation in group-based parenting programmes
- A proportionate universal approach, with both universal support available to all parents and targeted intervention programs for those in need of additional support, is recommended to improve children's outcomes.

Confidential: For Review Only

INTRODUCTION

The first three years of life are a critical period for childhood development, which encompasses the development of physical, mental, social, and emotional capabilities [1,2]. Early childhood development promotes future educational achievement and health [3,4], and persistent difficulties with early cognitive and socioemotional development can lead to worse physical and mental health in late adolescence [5]. Prevention and early intervention to promote strong early development for all children may reduce socioeconomic disparities across the lifespan [6].

Socioeconomic position and child development

Health inequalities are unfair and avoidable differences related to personal, demographic and socioeconomic characteristics. The majority of the relevant literature demonstrating pervasive socioeconomic inequalities in early child development in the UK is based on cohort studies, across various socioeconomic exposures and multiple domains of child development [7]. The Millennium Cohort Study (MCS) (N=19,517), is a nationally representative sample of children born 2000–2002. In MCS, outcomes examined at age 3 include socioemotional difficulties measured via the Strengths and Difficulties Questionnaire (SDQ) and cognitive development measured via the Bracken Basic Concept Scale. At age 5, socioemotional difficulties were measured again via the SDQ, and verbal ability via the British Ability Scales. In MCS, higher parental education predicted higher verbal ability at age 5 [8], and higher family socioeconomic position predicted lower socioemotional difficulties at ages 3 and 5 [9]. Families with the lowest incomes had children with a higher prevalence of both socioemotional and cognitive difficulties at both 3-years-old and 5-years-old [10]. A study using the Avon Longitudinal Study of Parents and Children (ALSPAC, N=13,855) similarly reported that lower parental occupation, education, and financial security were associated with lower expressive and receptive language at 2 years old measured as a modification of the MacArthur Communicative Development Inventory (CDI) [11].

Socioeconomic position may impact children's development through multiple mechanisms, for instance, socioeconomic advantage may afford parents better resources for enhancing the home environment of children, including via educational inputs such as play and stimulation, and emotional inputs such as parent-child relationships and interaction [12]. Socioeconomic advantage may also provide higher education for enhancing child development through cognitive stimulation and more complex language use. On the other hand, socioeconomic disadvantage may have a negative impact upon children's development through poor parental mental health and stress impacting upon parental engagement [12–14]. Subjective social status captures a person's perception of their social class relative to other people, and may impact on children's outcomes through a heightened experience of chronic stress responses [15]. It is therefore of interest to compare the magnitude of associations for multiple measures, including not only traditional measures such as income and education, but also subjective aspects of societal position [16]. This can give insights into the mechanisms by which family socioeconomic position influences children's development [17].

Subjective measures

For socioeconomically deprived families, subjective measures may be more sensitive predictors of children's outcomes than measures of income, as they can more accurately assess whether lower-income families feel they can provide for basic needs [17]. For example, in the Born in Bradford cohort, parents' self-reported financial security more strongly predicted birth outcomes, in comparison to parental education, receipt of means-tested benefits, and paternal occupation [18]. Further, subjective social status indicators such as the Macarthur Scale of Subjective Social Status may encourage consideration of perceptions of relative inequality, and this has been associated with cognitive function and self-rated health in adulthood [19]. Within the sample used in this study, most pregnant women rate themselves as having higher subjective social

Confidential: For Review Only

status than others in their neighbourhood, despite living in socioeconomically deprived areas [15]. Whilst self-reported social status appears to be related to adult wellbeing [20], it is yet to be investigated with regards to children's outcomes.

Parental social support refers to the existence of social networks [21]. Although social support is not directly a measure of a family's socioeconomic position, it can be considered a specific manifestation of social capital, which refers to the resources that are obtained via a person's social networks [22,23]. Parental social support could alleviate challenges during the transition to parenthood, hence supporting childhood outcomes [21], and could potentially mitigate against the negative effects of socioeconomic disadvantage for socioeconomically disadvantaged families. Emerging evidence suggests that higher levels of social support during pregnancy are associated with improved cognitive development at age 2 [24], and lower prevalence of developmental delay at age 3 [25].

Ethnicity and child development

As ethnic minority groups tend to experience higher levels of socioeconomic disadvantage in England [26], a consideration of differences in child development by ethnicity might reveal differential impacts of socioeconomic position. Indeed, an MCS study revealed that most ethnic minority groups have lower scores in cognitive and socioemotional outcomes compared to White British children [12]. A study of routinely collected health visiting data between 2018-2021, with a sample of 226,505 children at age 2-2 ½ years, found that 86% of children met expected levels of development overall, but that children from the most deprived neighbourhoods (82.6%), and children recorded as Black ethnicity (78.9%) were less likely to meet expected level of development [27].

Socioeconomic gradients within ethnic groups

Previous research has demonstrated that socioeconomic differences appear to be less pronounced in ethnic minority groups than ethnic majority groups for maternal and child health outcomes when comparing Pakistani born to UK born mothers [18,28], child socioemotional difficulties when comparing White to ethnic minority groups [29] and working memory when comparing Pakistani to White British children [30]. This lack of socioeconomic gradient may be due to difficulties with measurement, for instance inaccurate measurements of socioeconomic position in ethnic minority groups (e.g., educational attainment if received in a different country), or smaller samples in minority groups being underpowered to detect associations. Or, these findings may be due to a true lack of relationship between socioeconomic position and children's outcomes for ethnic minority groups. Subjective measures of socioeconomic position may aid our understanding of relationships between socioeconomic position and child outcomes for ethnic minority groups, as they may be more relevant than traditional measures (e.g. education), and may also provide an insight into the mechanisms by which minority families mitigate against the negative effects of disadvantage. Though, the relationship between ethnicity and children's outcomes is likely to be influenced by a variety of other factors beyond socioeconomic position, such as differences in culture, and experiences of prejudice, discrimination and racism [31]. Hence, larger samples of ethnic minorities, and a variety of socioeconomic measures, are needed to investigate the true impacts of socioeconomic position across diverse ethnic groups.

Present study and rationale

The present study uses data from the Born in Bradford's Better Start (BiBBS) interventional birth cohort, which has recruited a representative sample of 5,758 pregnant mothers and their children living in three inner-city areas of Bradford. BiBBS recruited families throughout the time period that 'Better Start' interventions were being delivered (1st January 2016 to 31st July 2024). Whilst BiBBS was specifically designed to

Confidential: For Review Only

undertake efficient and pragmatic evaluations of these multiple early life interventions, the cohort data can also be used for understanding how inequalities develop in disadvantaged populations. The mothers in the BiBBS cohort are predominantly from ethnic minority backgrounds (88%), and live in the most deprived decile of the Index of Multiple Deprivation (84%) [32].

This under-researched population provides the opportunity to understand potential differences in children's development by both subjective and objective socioeconomic measures, enabling an investigation of whether socioeconomic differences exist within disadvantaged contexts, and among different ethnic groups. This may also provide insights into which factors may protect against the development of poor childhood development in a predominantly socioeconomically disadvantaged population, and which groups of children may need more support. Building on previous research which has tended to focus on one specific aspect of child development at a time, we use the Ages and Stages Questionnaire, which is a broad measure of child development that encompasses communication, fine and gross motor skills, problem solving, and personal-social development. In this study we:

1. Describe the prevalence of children at risk of poor child development overall
2. Investigate which individual indicators of socioeconomic position predict early child development
3. Investigate if the association between individual indicators of socioeconomic position and early child development varies by ethnic group

Confidential: For Review Only

METHODS

Study design

This is a secondary analysis of observational longitudinal cohort study data. We have used the Strengthening the Reporting of Observational Studies in Epidemiology (STROBE) (2021) guidelines for reporting methods (see Supplementary File A) [33].

Study setting

This study is set in Bradford, a city in Northern England with high levels of socioeconomic deprivation and a large ethnic minority population [34]. Part of the Born in Bradford (BiB) research programme, Born in Bradford's Better Start (BiBBS) captures in-depth baseline data during pregnancy, and participants provide written consent for routine linkage to their own and their children's health, education, and intervention participation records. Pregnant women were invited to join BiBBS by trained researchers in person. Recruitment took place predominantly at Bradford Royal Infirmary's Glucose Tolerance Test (GTT) clinic at 24-28 weeks gestation which is offered to all women booked for delivery at BRI, and secondary recruitment sources in the community [32,35]. An interim BiBBS cohort profile for mothers recruited between 2016 and 2020 was shown to be broadly representative of the pregnant population in terms of ethnicity, parent age, parity, language ability, and area deprivation [32].

Data

All predictive measures were measured during the BiBBS baseline questionnaire, administered when mothers were approximately 26 weeks pregnant with their child. We linked Health Visiting records to provide the Ages and Stages Questionnaire (ASQ) outcome for this study which was collected approximately 2-2.5-years post birth. Inclusion for this study was all recruited BiBBS pregnancies between January 2016 and December 2022, who had an ASQ recorded for that pregnancy at the 2-2 ½ year visit by October 2024 (the time of the data drop from the data source).

Inclusion of covariates was decided using a Directed Acyclic Graph (DAG) (see below), and the analytical form of variables is described below.

Measures

We used four indicators of socioeconomic position. First, maternal education was categorised into 'No qualifications', '5 or less GCSEs', '5 or more GCSEs', 'A levels', and 'Degree'. Second, financial security was ascertained by mother's response to "how well are you managing financially?" Responses were coded as: 'Just about getting by', 'Finding it quite/very difficult', 'Doing alright', 'Living comfortably'. Third, subjective social status was measured via the MacArthur Scale of Subjective Social Status, an image of a ladder with 10 rungs, numbered from 1 at the bottom to 10 at the top. We used mother's responses to "Where would you place yourself on the ladder in relation to other people in your neighbourhood?", with answers analysed as continuous, ranging between 0 and 10. Fourth, social support was measured via responses to "how many people in your life can you count on in times of need?", again analysed as a continuous predictor, and answers ranged from 0 to 10. To test the sensitivity of the results to the linearity assumption, we estimated additional models with subjective social status and social support estimated as categorical variables, categorised into low (0-2 people, 1-2 ladder), medium (3-5 people, 3-8 ladder) and high (6 to 10 people, 9-10 ladder). Any 'do not wish to answer' and 'don't know' responses were recoded as missing. The lowest socioeconomic category was used as the reference category.

Confidential: For Review Only

For objective 2, maternal ethnicity was grouped into the largest ethnic groups within the cohort: White British, Pakistani heritage, White Other (containing White Irish, Polish, Slovakian, Romanian, Czech, Other White, Gypsy/Roma/Irish Traveller), Other South Asian (Indian & Bangladeshi), and Other (Chinese, African, Caribbean, Mixed, or Other). For objective 3, ethnic categories were further collapsed as the overlap between socioeconomic categories and ethnicity were too small for model estimation. Hence the groups were White British, South Asian (Pakistani, Indian & Bangladeshi) and Other. White British were the reference group.

There were three binary covariates: (1) Maternal country of birth was analysed as Not born in the UK/Born in the UK, (2) Maternal first spoken language was analysed as Not English/English, and (3) Child sex was analysed as Female/Male. There was one continuous covariate, (4) child age in months at the date of the most recent ASQ.

Outcome measure (Child development via Ages and Stages Questionnaire (ASQ))

All parents living in England receive a visit from their health visitor when their child turns 2-years-old, during which children's development is assessed using the 24, 27, or 30 month Ages and Stages Questionnaire (ASQ). ASQ-3 was developed to screen for developmental delay and comprises 21 age specific questionnaires for children aged between 1 month and 66 months [36]. ASQ-3 covers five key domains of developmental status of a child's following areas [37]: Communication - babbling, vocalizing, listening, and understanding; Gross Motor: arm, body, and leg movements; Fine Motor: hand and finger movements; Problem Solving: learning and playing with toys; and Personal-Social: solitary social play and play with toys and other children. Several studies have shown the ASQ-3 to be a valid screening tool to identify developmental concerns for individual children, though there is a lack of its validation in England specifically, and for ethnic minority groups [38,39].

There are three response options to each question (yes/sometimes/not yet, with scores being yes=10, sometimes=5 and not yet=0). The score of each domain ranges between 0 and 60, however each age version and domain of the ASQ has different cut-offs to categorise children into; (1) 'below cut-off', where a child requires further assessment and intervention with a professional, (2) 'monitor', where a child's development should be monitored, and (3) 'above cut off', where a child's development is on schedule. For objective 2, in line with the Public Health England method for calculating whether children have a Good Level of Development (GLD), an overall ASQ score was created by categorising as 'Not at risk'=above cut off and/or monitor on all 5 domains, and 'At risk'='below cut off' on 1 or more domains (see https://fingertips.phe.org.uk/static-reports/health-trends-in-england/England/best_start_in_life.html; [27]).

For objective 3, to create an outcome that was more sensitive to identifying children *potentially* at risk, an additional ASQ 'Full' GLD (FGLD) score was created by categorising as 'Not at risk'='above cut off' on all 5 domains and 'At risk'='below cut off' AND/OR 'monitor' on 1 or more domains. Scores were only calculated if all 5 domains were available. The FGLD method provides more variation in the outcome than the GLD method, which means it is more sensitive to identifying any existing socioeconomic gradients in outcomes.

Analysis

For objective 1 we described the number of children at risk of poor development overall using both the GLD and FGLD method. For reference we compare proportions of children at risk using the GLD method to Jung et al. (2025), who described child developmental outcomes in the ASQ-3 across England.

For objectives 2 and 3, a Directed Acyclic Graph (DAG) ascertained the adjustment set for the effect of each socioeconomic indicator (maternal education, financial management, social ladder, number of people to count on). Socioeconomic variables could be either mediators or confounders of specific associations depending on the relationship of interest, hence each relationship of interest required a separate adjustment

set and analysis [40]. Justification for inclusion of each confounder/covariate was based on prior literature [10,12]. We did not include area deprivation and/or area as a covariate, since the areas under study are relatively homogeneous in terms of deprivation [32].

- *Figure 1 placed here*

Figure 1. Directed Acyclic Graph with estimates of interest labelled 1-4.

**Model (#) and key predictor**	**Confounders included in model**	**Covariates included in model**
1. Maternal education	Migrant to UK, English is first language, Ethnic group	Child age, child sex
2. Financial management	Maternal education, Migrant to UK, English is first language, Ethnic group	Child age, child sex
3. Subjective social status (social ladder)	Maternal education, Migrant to UK, English is first language, Ethnic group	Child age, child sex
4. Social support (people to count on)	Maternal education, Migrant to UK, English is first language, Ethnic group	Child age, child sex

Table 1. Model number with key predictor, and covariates included in each model

For objective 1, the prevalence of children at risk of poor development was described using the GLD method. For objectives 2-3, binary logistic regression models were conducted to produce odds ratios and 95% confidence intervals using the FGLD method, and model-based estimates (ie. adjusted predictions) for the outcomes were presented using plots.

For objective 3, to investigate if socioeconomic differences in child development varied by ethnic group, we estimated four additional binary logistic models with ethnicity (categorised as White British, South Asian, and Other) and each socioeconomic predictor (maternal education, financial management, social ladder, and people to count on), with an interaction fitted between these variables. We applied the specified adjustment sets for each predictor using Table 1. A likelihood ratio test was performed to compare the fit of the models with and without the interaction by ethnic group, where the fit of the two competing models was compared and the statistic represents a direct comparison of the relative likelihood of the data, given the best fit of the two models [41].

Results were considered to be statistically significant if $p<.05$. R was used for all analysis, key packages included *tidyverse, ggplot2, ggdist*, and *ggeffects*. Full analysis code and results are available at https://osf.io/c9rup/overview?view_only=0bc318a438d54db98b01787ba13462c4 (link to be replaced with public link upon acceptance of paper).

Missing data

We applied complete case analysis, as this gives unbiased results when the chance of being a complete case does not depend on the outcome after taking the covariates into consideration [42]. The analysis therefore assumes that having complete data does not depend on a child's development, after taking into account the included predictor and control variables.

https://mc.manuscriptcentral.com/bmjpo

Confidential: For Review Only

Patient and public involvement

Members of the community are involved in the design and conduct of BiBBS cohort research, including recruitment, questionnaires, measures and interpretation and dissemination of findings, through regular Community Research Advisory Group meetings, composed of Better Start Bradford residents.

Confidential: For Review Only

RESULTS

Full analysis code and results are available at https://osf.io/c9rup/overview?view_only=0bc318a438d54db98b01787ba13462c4 (link to be replaced with public link upon acceptance of paper).

Sample

- *Figure placed here*

Figure 2. Eligible participants and numbers included in models for each objective

Figure 2 shows the number of pregnancies with ASQ records, and the sample sizes for each objective and model. The sample contained 2887 pregnancies with a 2-year visit recorded, and 2100 (72%) of them has an ASQ recorded. It is beyond the scope of this current study to explore reasons behind non completion of the ASQ, however, a mixed methods study by our team will explore this further [43]. The analysis sample for objective 1 contains all pregnancies with valid ASQs (n=2003), and the analysis sample for objectives 2-3 depends on the availability of the data within the sample (social status n=791, financial security n=1159, social support n=1191, and maternal education n=1214).

Objective 1 (prevalence of children at risk)Objective 1 was to estimate the number of children at risk of poor child development overall, and the number of children at risk of poor development in each domain. Within the 2,003 individual children with linked ASQs, 445 (22%) of all children were at risk of overall poor development using the GLD method, and 884 (44%) were at risk using the FGLD method.

Objective 2 (which socioeconomic indicators predict child development)Objective 2 was to investigate the association between individual indicators of socioeconomic position and early child development. The FGLD method was used as the outcome, which is more sensitive to identifying children potentially at risk of poor development (44%). Descriptives for all participants are presented in Supplementary Material B, and logistic regression models are presented in Supplementary Material C.

Odds ratios from adjusted logistic regressions between the predictor variables and achieving the expected level of development show that having a degree (OR=1.95, 95% CI 1.28 to 2.99), living comfortably (OR=1.78, 95% CI 1.03 to 3.07), and subjective social status (OR=1.11, 95%CI 1.02 to 1.22) all resulted in increased odds of having a FGLD. Social support was not associated with having a FGLD (OR=0.99, 95% CI 0.94 to 1.04).

- *Figures 3a-3d placed here as one –*

Figure 3a-3d. Adjusted predictions for models 1-4, with no interaction for ethnicity included.

Note: the overlap of 95% CIs in Figures a and b does not mean that the difference between any given pair of estimates is not significant at p<.05.

All socioeconomic predictors, except for social support, were significantly associated with the probability of expected development, and the figures visualise the socioeconomic gradients. The adjusted predictions reveal that the socioeconomic gradient is steepest by subjective social status, with the largest difference between the highest and lowest socioeconomic category for this predictor.

Confidential: For Review Only

Objective 3 (variation between socioeconomic position and child development by ethnic group)

Objective 3 was to investigate if the association between individual indicators of socioeconomic position and early child development varies by ethnic group. Results from the likelihood ratio tests indicated that the model with the interaction fitted the data equally well for maternal education (X^2=8.22, p=0.222) and financial security (X^2=4.34, p=0.461), whereas the model with the interaction was superior for subjective social status (X^2=2.90, p<0.001) and social support (X^2=6.26, p<0.001). The adjusted predictions (see below) indicate that subjective social status and support were less strongly related to child development for South Asian groups, in comparison to White British and Other ethnic groups. Full model results are in Supplementary File D, and adjusted predictions from each model are presented below. The sensitivity analysis including subjective social status and support as categorical predictors showed a similar pattern of results.

- *Figures 4a-4d placed here as one -*

Figure 4a-4d. Adjusted predictions for models 1-4, with interaction by ethnicity included.

Confidential: For Review Only

DISCUSSION

Our analysis of longitudinal, routinely collected data from this place-based birth cohort study reveals a concerningly high proportion of children in inner-city Bradford identified as being at risk of poor development by age two (22%). The prevalence of children at risk is considerably higher than a previously estimated average for England (14%) [27], underscoring the significant vulnerability of this specific population. This high rate of developmental risk will likely have long-lasting implications for these children's future educational attainment, health, and overall wellbeing [6].

Whilst maternal education and financial security had previously been established as important predictors of children's outcomes [7,18], our study shows the additional relevance of parent-reported social status. Subjective social status presented the steepest social gradient in children's outcomes in comparison to maternal education (18 percentage point gap), financial security (15 percentage point gap), and social support (2 percentage point gap), with a 26% percentage point gap between the highest and lowest points on the subjective social status scale. Whilst research linking subjective social status to early developmental outcomes is limited, this aligns with broader research indicating that subjective socioeconomic measures may more strongly predict birth outcomes in deprived populations, when compared to parental education [18]. This suggests that a self-reported, subjective assessment of social standing offers a more nuanced understanding of socioeconomic position within disadvantaged populations, potentially revealing how parents in deprived areas might mitigate the challenges of socioeconomic deprivation through their own perceived subjective social status.

Since this measure of subjective social status purportedly captures a person's perception of their social class relative to other people, this may indicate that a parent's perceptions of inequality has an impact on their children's outcomes through a heightened experience of chronic stress [15,44]. Since few epidemiological studies include both subjective and objective measures of socioeconomic position, further research is necessary. We propose that subjective socioeconomic measures may be particularly useful in ethnically diverse populations through potentially obtaining lower non-response rates compared to objective metrics (like exact income or foreign qualifications) that can be challenging to reliably report. Further research is needed regarding the validity and generalisability of socioeconomic measures across different ethnic groups, and their relationships with various outcomes. The lack of a significant association between social support and child outcomes contrasted with previous research that has indicated a positive relationship [24,25], however, further analysis revealed that this may be due to differences by ethnic group.

Whilst the socioeconomic gradients in maternal education and financial management appeared broadly consistent across White British, South Asian, and Other ethnic groups, a more nuanced picture emerged regarding the role of subjective social status and support. The adjusted predictions (Figures 4a-4d) revealed a relatively weaker relationship between subjective social status and support and child outcomes specifically within the South Asian group, (in comparison to the White British and Other ethnic groups). This suggests that the null result in the original social support model was perhaps driven by the weak association for South Asian groups, suggesting social support may be a protective factor for many families. Interestingly, a very strong association was apparent between social support and child development for the 'Other' ethnic group; which includes White Other (including White Irish, Polish, Slovakian, Romanian, Czech, Other White, Gypsy/Roma/Irish Traveller), Black African/Caribbean/Black British, and Mixed ethnic groups. As these groups represent the least

populous ethnic minorities in the BiBBS population, it may be that social support is more important for particular communities. Despite being an ethnic minority in England, the Pakistani population are the most populous ethnic group within the BiBBS area, perhaps meaning that they benefit from the psychosocial resilience offered by same-ethnic density, and that social support at a more individual level is not as crucial for improving child outcomes [45,46]. The lack of association for South Asian groups aligns with existing research that has reported a lack of expected socioeconomic gradients for maternal and child outcomes among South Asian groups [18,28,30], though these previous studies used different socioeconomic indicators. Our study extends this by demonstrating that a subjective measure of social support may have different relationships with children's outcomes depending on ethnic group, and may be particularly important for less populous ethnic minorities.

However, this finding could also reflect unmeasured confounding or inaccurate measurement. For instance, our measure of social support might not capture the *quality* or *type* of support received, which could be more influential than the quantity of social connections. Given that South Asian families in this area are often characterised by close proximity to family members living in the same households [47], the nature and dynamics of this support might differ significantly from other groups.

Altogether, the strong relationship between subjective social status and child outcomes may have implications for early interventions that support families facing socioeconomic disadvantage. This may suggest that early parenting interventions should not only provide educational material and financial aid to families, but also address the psychological impacts associated with low subjective social status [44]. This indicates the importance of the social environment for parents of young children, highlighting the role that group-based parenting programmes could play in building social networks and social cohesion for parents of young children [48]. However, as we found that the importance of subjective social status and support may vary by ethnic group, such interventions may work well for parents of some ethnicities, but not for others.

Ultimately, the relationship between ethnicity and children's outcomes is of course influenced by a multitude of factors beyond socioeconomic position, such as cultural practices, prejudice and racism [31]. Early years practitioners working with families should be mindful of potential cultural nuances in how social factors influence child development within different communities. To gain a more comprehensive understanding of the true impact of socioeconomic position across diverse ethnic groups, future research should continue to employ larger samples of ethnic minorities and apply a wider array of both objective and subjective socioeconomic measures.

Strengths and limitations

Findings from BiBBS may generalise to many similar urban populations, especially those with high ethnic diversity and socioeconomic deprivation. The large number of ethnic minorities recruited (e.g. n=979 Pakistani, 49% of the sample) improves our statistical power to detect differences and builds on previous research, which may have been limited by smaller numbers of ethnic minorities. However, the disadvantaged population under study may be relatively homogeneous in terms of socioeconomic characteristics, reducing the potential for observing significant social gradients. It was not possible to include income as a predictor in this study, as this was not included in the baseline questionnaire due to low acceptability in this population. Some families were missing a baseline questionnaire (around 25%) due to changes to recruitment procedures during COVID, and some children were missing the ASQ outcome (28%, see Figure 1). Hence our complete case analysis is only valid under the assumption having complete data does not depend on a child's development, after taking into account

Confidential: For Review Only

the included predictor and control variables. Whilst we tentatively suggest that the associations observed here might be causal and the longitudinal design of the study facilitates this, the study is observational and hence we cannot rule out the presence of unmeasured confounders which could be driving the associations that we present.

Conclusion and implications

A high proportion of children do not meet expected levels of development in this ethnically diverse, socioeconomically disadvantaged population. Higher socioeconomic position may protect against poor early development in such communities, and the mechanism of this may differ by ethnic group. The high prevalence of children at risk can be partly explained by socioeconomic position, and this suggests a continued need for broader public health initiatives that meet the needs of families living in disadvantaged settings, such as proportionate universal approaches that encompass both universal prevention and early targeted interventions for those who need additional support [6]. This necessitates investment in early years practitioners, such as health visitors, who can work directly in these communities to identify children at risk, and connect them with relevant support services.

Confidential: For Review Only

REQUIRED STATEMENTS

Acknowledgements

The integration of research and practice in Bradford has only been possible because of the enthusiasm and commitment of staff and volunteers across children's services in Bradford. We are grateful to all Born in Bradford staff, the Better Start Bradford partnership and staff, all Better Start Bradford project teams, health professionals, local authority and voluntary and community sector organisations who have supported the integration of research into practice. We are grateful to all the families taking part in BiBBS and all members of the Community Research Advisory Group. A poster regarding this study was presented at the International Network for Research Inequalities in Child Health (INRICH) workshop in 2025.

Funding statement

This study has received funding from the National Lottery Community Fund (previously the Big Lottery Fund) as part of the A Better Start programme (Ref 10094849). The funder was not involved in the design of the study and collection, analysis, and interpretation of data nor in writing the manuscript.

SLB, JD, and KP are also supported by the NIHR Yorkshire and Humber Applied Research Collaboration (ARC-YH; Ref: NIHR200166, see https://www.arc-yh.nihr.ac.uk). The views in this publication are those of the authors and not necessarily those of the NIHR or the Department of Health and Social Care.

Competing interests

The authors have no competing interests to declare.

Authors contributions

KEM is responsible for the overall content as the guarantor. KEM conducted the analysis and drafted the manuscript. MW cleaned and linked the BiBBS baseline with the health visiting datasets. KEP, SLB, JL and JD all reviewed the manuscript and provided substantial contributions.

Ethical approval

The protocol for BiBBS recruitment and collection of routine outcome data was approved by Bradford Leeds NHS Research Ethics Committee (15/YH/0455). Research governance approval was gained from Bradford Teaching Hospitals NHS Foundation Trust. The existing ethics includes approval for both evaluation of Better Start Bradford interventions, and observational research. Hence, no further consent was sought for this study.

Data availability

Data cannot be shared publicly as they are available through a system of managed open access. Researchers interested in accessing the data can find details and procedures on the Born in Bradford website (https://borninbradford.nhs.uk/research/how-to-access-data/). Data access is subject to review by the Born in Bradford Executive, who review proposals on a monthly basis. Requests can be submitted to borninbradford@bthft.nhs.uk. Data sharing agreements are established between the researcher and the Bradford Institute for Health Research. Full analysis code and results are available

Confidential: For Review Only

at https://osf.io/c9rup/overview?view_only=0bc318a438d54db98b01787ba13462c4 (link to be replaced with public link upon acceptance of paper).

Confidential: For Review Only

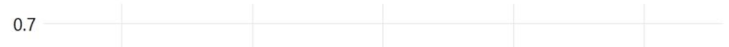

Confidential: For Review Only

REFERENCES

1 Tierney AL, Nelson CA. Brain Development and the Role of Experience in the Early Years. *Zero Three*. 2009;30:9.

2 Newman L, Judd F, Olsson CA, *et al.* Early origins of mental disorder - risk factors in the perinatal and infant period. *BMC Psychiatry*. 2016;16:1–8. doi: 10.1186/s12888-016-0982-7

3 Murrah WM, III. Comparing Self-Regulatory and Early Academic Skills as Predictors of Later Math, Reading, and Science Elementary School Achievement. *ProQuest LLC*. 2010;102.

4 Amin V, Behrman JR, Spector TD. Does more schooling improve health outcomes and health related behaviors? Evidence from U.K. twins. *Econ Educ Rev*. 2013;35:134–48. doi: 10.1016/j.econedurev.2013.04.004

5 Black M, Akanni L, Adjei NK, *et al.* Impact of child socioemotional and cognitive development on exam results in adolescence: findings from the UK Millennium Cohort Study. *Arch Dis Child*. 2025;0:1–6. doi: 10.1136/ARCHDISCHILD-2024-327963

6 Marmot M, Allen J, Goldblatt P, *et al.* The Marmot review: Fair society, healthy lives. *The Strategic Review of Health Inequalities in England Post-2010*. 2010.

7 Pillas D, Marmot M, Naicker K, *et al.* Social inequalities in early childhood health and development: a European-wide systematic review. *Pediatric Research 2014 76:5*. 2014;76:418–24. doi: 10.1038/pr.2014.122

8 Becker B. Social disparities in children's vocabulary in early childhood. Does pre-school education help to close the gap?1. *Br J Sociol*. 2011;62:69–88. doi: 10.1111/J.1468-4446.2010.01345.X

9 Flouri E, Tzavidis N, Kallis C. Area and family effects on the psychopathology of the Millennium Cohort Study children and their older siblings. *Journal of Child Psychology and Psychiatry*. 2010;51:152–61. doi: 10.1111/J.1469-7610.2009.02156.X

10 Kelly Y, Sacker A, Del Bono E, *et al.* What role for the home learning environment and parenting in reducing the socioeconomic gradient in child development? Findings from the Millennium Cohort Study. *Arch Dis Child*. 2011;96:832–7. doi: 10.1136/adc.2010.195917

11 Lai ETC, Wickham S, Law C, *et al.* Poverty dynamics and health in late childhood in the UK: evidence from the Millennium Cohort Study. *Arch Dis Child*. 2019;archdischild-2018-316702. doi: 10.1136/archdischild-2018-316702

12 Cattan S, Fitzsimons E, Goodman A, *et al.* Early childhood inequalities. *Oxford Open Economics*. 2024;3:i711–40. doi: 10.1093/OOEC/ODAD072

13 Duncan GJ, Magnuson K. Socioeconomic status and cognitive functioning: moving from correlation to causation. *Wiley Interdiscip Rev Cogn Sci*. 2012;3:377–86. doi: 10.1002/wcs.1176

14 Carneiro P, Cattan S, Neves H. Theoretical and empirical perspectives on the link between poverty, parenting and children's outcomes. *Fisc Stud*. 2025;46:9–35. doi: 10.1111/1475-5890.12404

15 Moss RH, Kelly B, Bird PK, *et al.* Turning their backs on the 'ladder of success'? Unexpected responses to the MacArthur Scale of Subjective Social Status. *Wellcome Open Res*. 2023;8:11. doi: 10.12688/WELLCOMEOPENRES.18655.2

16 Galobardes B, Shaw M, Lawlor DA, *et al.* Indicators of socioeconomic position (part 1). *J Epidemiol Community Health (1978)*. 2006;60:7–12. doi: 10.1136/jech.2004.023531

17 Ursache A, Noble KG. Neurocognitive development in socioeconomic context: Multiple mechanisms and implications for measuring socioeconomic status. *Psychophysiology*. 2016;53:71–82. doi: 10.1111/psyp.12547

18 Uphoff EP, Pickett KE, Wright J. Social gradients in health for Pakistani and White British women and infants in two UK birth cohorts. *Ethn Health*. 2016;21:452–67. doi: 10.1080/13557858.2015.1091442

19 Präg P. Subjective socio-economic status predicts self-rated health irrespective of objective family socio-economic background. *Scand J Public Health*. 2020;48:707–14. doi: 10.1177/1403494820926053/SUPPL_FILE/SJP926053_SUPPLEMENTAL_MATERIAL.PDF

20 Heitz HK, Mitchell AM. Subjective Social Status and Mental Health Outcomes: The Role of Mindfulness. *J Prev Health Promot*. 2022;3:445–67. doi: 10.1177/26320770221087248

21 Wickramaratne PJ, Yangchen T, Lepow L, *et al.* Social connectedness as a determinant of mental health: A scoping review. *PLoS One*. 2022;17:e0275004. doi: 10.1371/JOURNAL.PONE.0275004

22 Villalonga-Olives E, Kawachi I. The dark side of social capital: A systematic review of the negative health effects of social capital. *Soc Sci Med*. 2017;194:105–27. doi: 10.1016/J.SOCSCIMED.2017.10.020

23 Saegert S, Carpiano RM. Social support and social capital: A theoretical synthesis using community psychology and community sociology approaches. *APA handbook of community psychology: Theoretical foundations, core concepts, and emerging challenges*. 2016;295–314. doi: 10.1037/14953-014

24 Shin EK, Lewinn K, Bush N, *et al.* Association of Maternal Social Relationships With Cognitive Development in Early Childhood. *JAMA Netw Open*. 2019;2:e186963–e186963. doi: 10.1001/JAMANETWORKOPEN.2018.6963

25 Imanishi Y, Ikehara S, Aochi Y, *et al.* The association between maternal social support levels during pregnancy and child development at three years of age: the Japan Environment and Children's Study. *Environ Health Prev Med*. 2024;29:18–18. doi: 10.1265/EHPM.23-00211

26 Chattoo S, Atkin K. *Understanding 'race' and ethnicity 2e: Theory, history, policy, practice.* 2nd ed. Policy Press 2019.

Confidential: For Review Only

27 Jung J, Cattan S, Powell C, *et al.* Early child development in England: cross-sectional analysis of ASQ®-3 records from the 2-2½-year universal health visiting review using national administrative data (Community Service Dataset, CSDS). *Int J Popul Data Sci*. 2025;9:2459. doi: 10.23889/IJPDS.V9I2.2459

28 Mallicoat B, P Uphoff E, E Pickett K. Estimating Social Gradients in Health for UK Mothers and Infants of Pakistani Origin: Do Latent Class Measures of Socioeconomic Position Help? *J Immigr Minor Health*. Published Online First: 2020. doi: 10.1007/s10903-020-00977-9

29 Kuo YL, Casillas A, Walton KE, *et al.* The intersectionality of race/ethnicity and socioeconomic status on social and emotional skills. *J Res Pers*. 2020;84:103905. doi: 10.1016/j.jrp.2019.103905

30 Mooney KE, Pickett KE, Shire K, *et al.* Socioeconomic disadvantage and ethnicity are associated with large differences in children's working memory ability: analysis of a prospective birth cohort study following 13,500 children. *BMC Psychol*. 2022;10:67. doi: 10.1186/s40359-022-00773-0

31 Marks AK, Garcia Coll C. Education and developmental competencies of ethnic minority children: Recent theoretical and methodological advances. *Developmental Review*. 2018;50:90–8. doi: 10.1016/j.dr.2018.05.004

32 Dickerson J, Bridges S, Willan K, *et al.* Born in Bradford's Better Start (BiBBS) interventional birth cohort study: Interim cohort profile. *Wellcome Open Research 2022 7:244*. 2022;7:244. doi: 10.12688/wellcomeopenres.18394.1

33 Ghaferi AA, Schwartz TA, Pawlik TM. STROBE Reporting Guidelines for Observational Studies. *JAMA Surg*. 2021;156:577–8. doi: 10.1001/JAMASURG.2021.0528

34 Bradford Council. Poverty in Bradford District | Understanding Bradford District. 2019. https://ubd.bradford.gov.uk/about-us/poverty-in-bradford-district/ (accessed 1 February 2021)

35 Dickerson J, Bird PK, McEachan RRC, *et al.* Born in Bradford's Better Start: An experimental birth cohort study to evaluate the impact of early life interventions. *BMC Public Health*. 2016;16:711. doi: 10.1186/s12889-016-3318-0

36 Velikonja T, Edbrooke-Childs J, Calderon A, *et al.* The psychometric properties of the Ages & Stages Questionnaires for ages 2-2.5: a systematic review. *Child Care Health Dev*. 2017;43:1–17. doi: 10.1111/CCH.12397

37 Squires J, Bricker D. Ages & Stages Questionnaires [R], (ASQ-3 [TM]): A Parent-Completed Child-Monitoring System. *Brookes Publishing Company*. 2009.

38 Kendall S, Nash A, Braun A, *et al.* Evaluating the use of a population measure of child development in the Healthy Child Programme Two Year Review. Policy Research Unit in the Health of Children, Young People and Families* Centre for Research in Primary and Community Care**. 2014.

Confidential: For Review Only

39 Kendall S, Nash A, Braun A, *et al.* Acceptability and understanding of the Ages & Stages Questionnaires®, Third Edition, as part of the Healthy Child Programme 2-year health and development review in England: Parent and professional perspectives. *Child Care Health Dev*. 2019;45:251–6. doi: 10.1111/CCH.12639

40 Shrier I, Platt RW. Reducing bias through directed acyclic graphs. BMC Med Res Methodol. 2008;8:1–15.

41 Glover S, Dixon P. Likelihood ratios: A simple and flexible statistic for empirical psychologists. *Psychon Bull Rev*. 2004;11:791–806. doi: 10.3758/BF03196706/METRICS

42 Hughes RA, Heron J, Sterne JAC, *et al.* Accounting for missing data in statistical analyses: multiple imputation is not always the answer. *Int J Epidemiol*. 2019;48:1294–304. doi: 10.1093/IJE/DYZ032

43 Henderson HC, Mooney KE, Morton K, *et al.* Use of the Ages and stages questionnaire in Bradford as a measure of Child Development (ABCD): a mixed methods study protocol. *medRxiv*. 2025;2025.08.14.25333745. doi: 10.1101/2025.08.14.25333745

44 Singh-Manoux A, Adler NE, Marmot MG. Subjective social status: its determinants and its association with measures of ill-health in the Whitehall II study. *Soc Sci Med*. 2003;56:1321–33. doi: 10.1016/S0277-9536(02)00131-4

45 Pickett KE, Wilkinson RG. People like us: Ethnic group density effects on health. Ethn Health. 2008;13:321–34.

46 Pickett KE, Shaw RJ, Atkin K, *et al.* Ethnic density effects on maternal and infant health in the Millennium Cohort Study. *Soc Sci Med*. 2009;69:1476–83. doi: 10.1016/j.socscimed.2009.08.031

47 Thapar-Bjorkert S, Sanghera G. Social capital, educational aspirations and young Pakistani Muslim men and women in Bradford, West Yorkshire. 2010.

48 Barlow J, Stewart-Brown S. Understanding parenting programmes: parents' views. *Prim Health Care Res Dev*. 2001;2:117–30. doi: 10.1191/146342301678787067

49 Mooney KE, Bywater T, Hinde S, *et al.* A quasi-experimental effectiveness evaluation of the 'Incredible Years Toddler' parenting programme on children's development aged 5: A study protocol. *PLoS One*. 2023;18:e0291557. doi: 10.1371/JOURNAL.PONE.0291557

Confidential: For Review Only

FIGURE LEGENDS

Figure 1. Directed Acyclic Graph with estimates of interest labelled 1-4.

Figure 2. Eligible participants and numbers included in models for each objective

Figure 3a-3d. Adjusted predictions for models 1-4, with no interaction for ethnicity included.

0.7

Figure 4a-4d. Adjusted predictions for models 1-4, with interaction by ethnicity included.

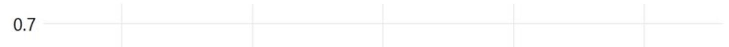

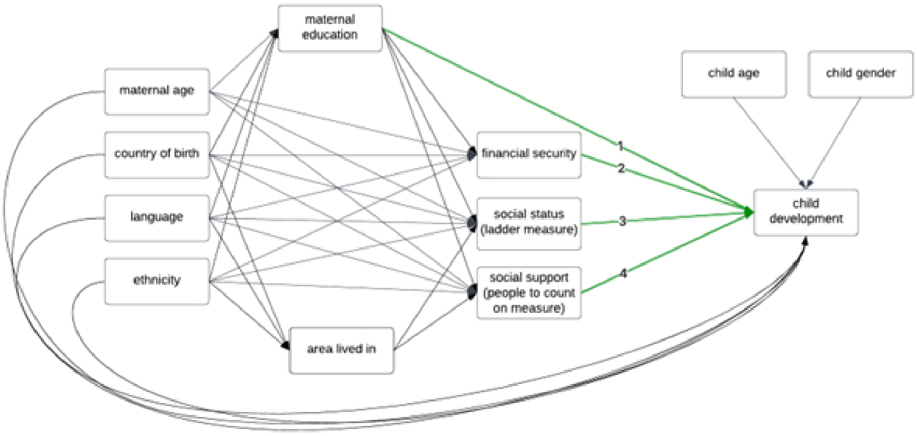

Figure 1. Directed Acyclic Graph with estimates of interest labelled 1-4.

429x195mm (38 x 38 DPI)

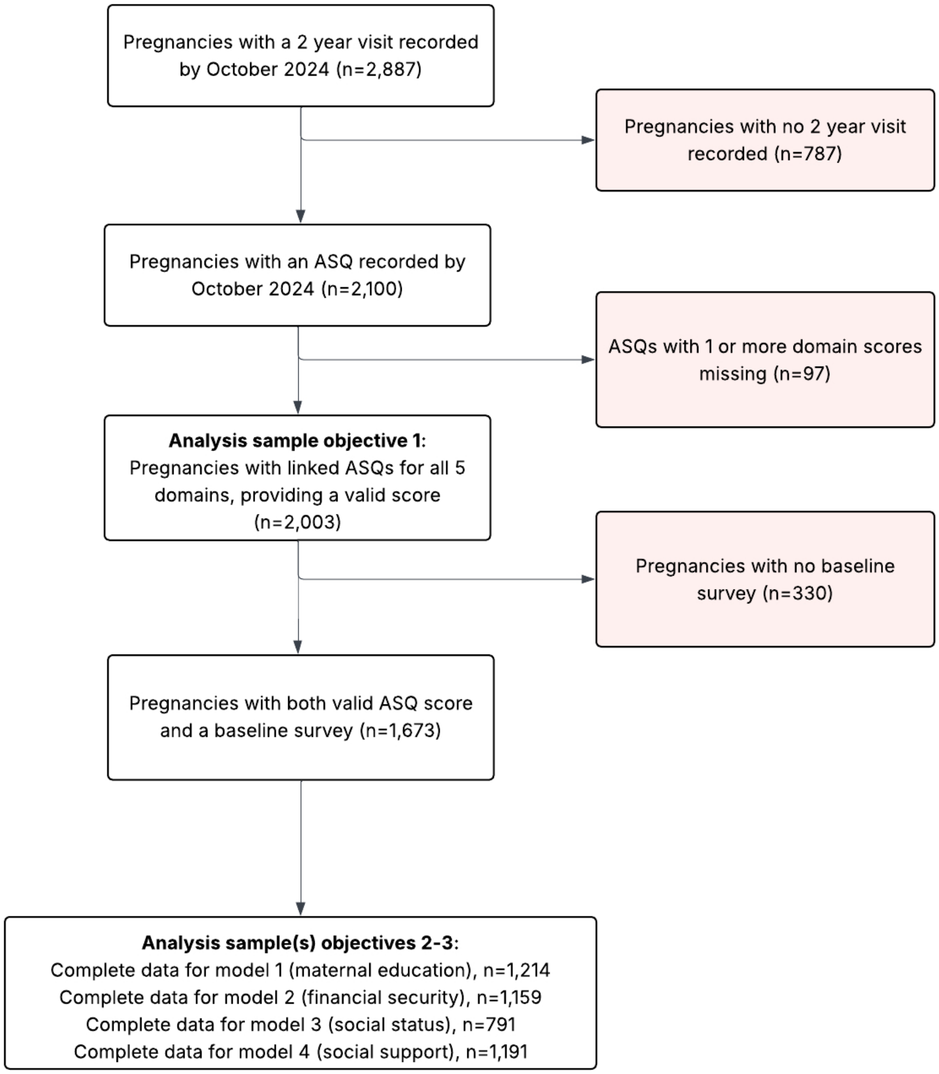

Figure 2. Eligible participants and numbers included in models for each objective

477x545mm (63 x 63 DPI)

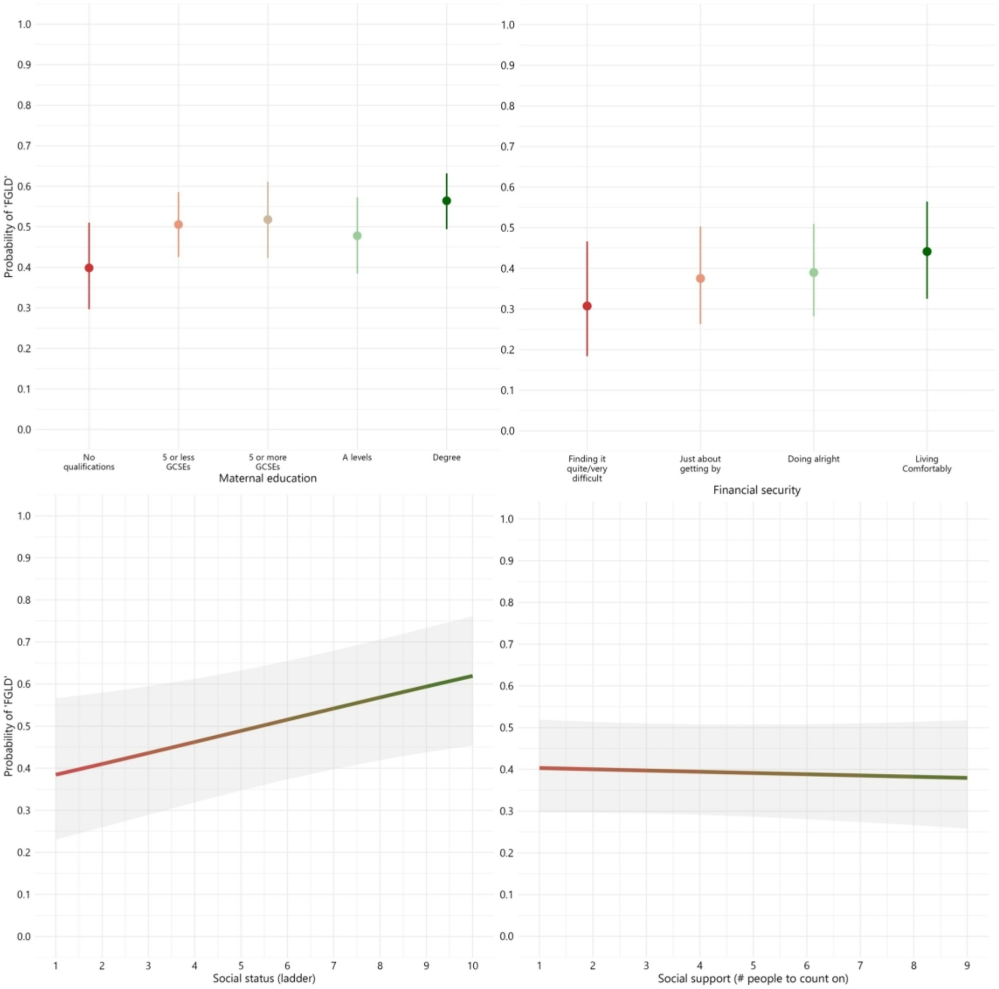

Figure 3a-3d. Adjusted predictions for models 1-4, with no interaction for ethnicity included.

178x179mm (300 x 300 DPI)

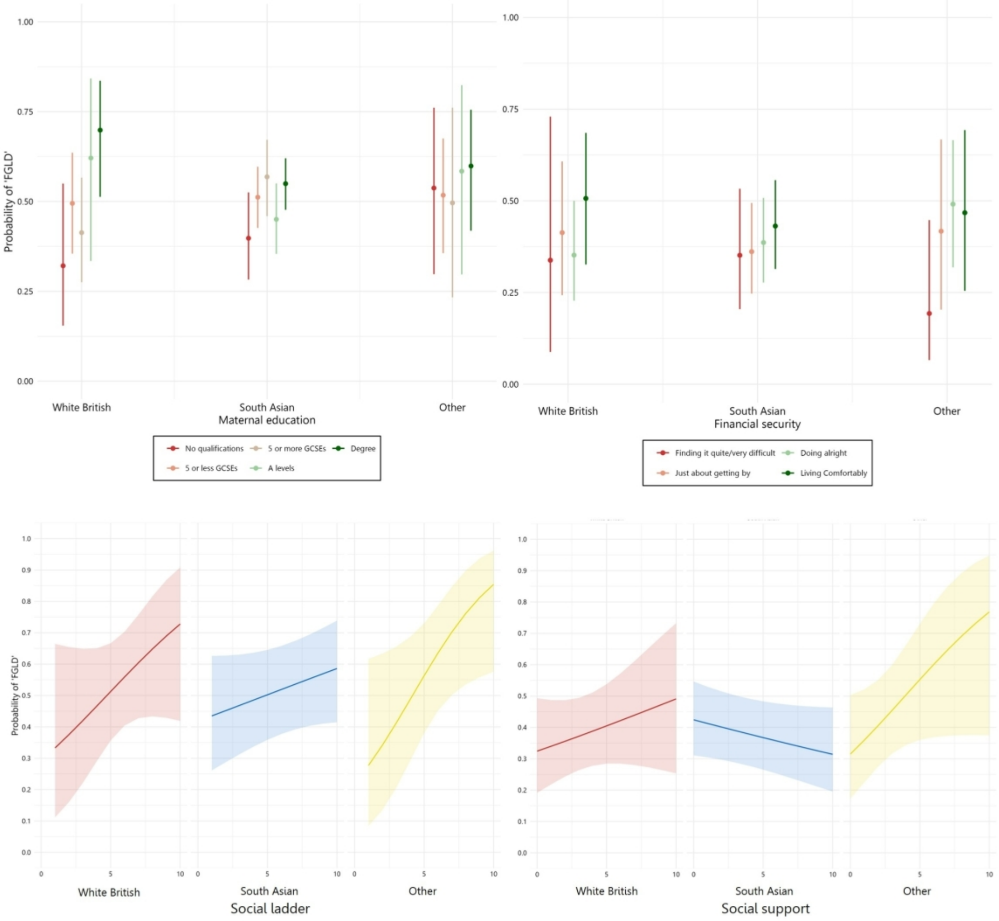

Figure 4a-4d. Adjusted predictions for models 1-4, with interaction by ethnicity included.

176x166mm (300 x 300 DPI)

STROBE Statement—Checklist of items that should be included in reports of ***cohort studies***

	**Item No**	**Recommendation**	**Page No**
**Title and abstract**	1	(*a*) Indicate the study's design with a commonly used term in the title or the abstract	1
		(*b*) Provide in the abstract an informative and balanced summary of what was done and what was found	1
**Introduction**			
Background/rationale	2	Explain the scientific background and rationale for the investigation being reported	4-6
Objectives	3	State specific objectives, including any prespecified hypotheses	6
**Methods**			
Study design	4	Present key elements of study design early in the paper	7
Setting	5	Describe the setting, locations, and relevant dates, including periods of recruitment, exposure, follow-up, and data collection	7-8
Participants	6	(*a*) Give the eligibility criteria, and the sources and methods of selection of participants. Describe methods of follow-up	7-8
		(*b*) For matched studies, give matching criteria and number of exposed and unexposed	NA
Variables	7	Clearly define all outcomes, exposures, predictors, potential confounders, and effect modifiers. Give diagnostic criteria, if applicable	7-8
Data sources/ measurement	8*	For each variable of interest, give sources of data and details of methods of assessment (measurement). Describe comparability of assessment methods if there is more than one group	8
Bias	9	Describe any efforts to address potential sources of bias	8-9
Study size	10	Explain how the study size was arrived at	11
Quantitative variables	11	Explain how quantitative variables were handled in the analyses. If applicable, describe which groupings were chosen and why	9
Statistical methods	12	(*a*) Describe all statistical methods, including those used to control for confounding	8-9
		(*b*) Describe any methods used to examine subgroups and interactions	8-9
		(*c*) Explain how missing data were addressed	10
		(*d*) If applicable, explain how loss to follow-up was addressed	NA
		(*e*) Describe any sensitivity analyses	NA
**Results**			
Participants	13*	(a) Report numbers of individuals at each stage of study—eg numbers potentially eligible, examined for eligibility, confirmed eligible, included in the study, completing follow-up, and analysed	11
		(b) Give reasons for non-participation at each stage	11
		(c) Consider use of a flow diagram	11
Descriptive data	14*	(a) Give characteristics of study participants (eg demographic, clinical, social) and information on exposures and potential confounders	12
		(b) Indicate number of participants with missing data for each variable of interest	12
		(c) Summarise follow-up time (eg, average and total amount)	12

Confidential: For Review Only

Outcome data	15*	Report numbers of outcome events or summary measures over time	11
Main results	16	(a) Give unadjusted estimates and, if applicable, confounder-adjusted estimates and their precision (eg, 95% confidence interval). Make clear which confounders were adjusted for and why they were included	No unadjusted estimates, 13
		(b) Report category boundaries when continuous variables were categorized	7
		(c) If relevant, consider translating estimates of relative risk into absolute risk for a meaningful time period	NA
Other analyses	17	Report other analyses done—eg analyses of subgroups and interactions, and sensitivity analyses	NA
**Discussion**			
Key results	18	Summarise key results with reference to study objectives	13
Limitations	19	Discuss limitations of the study, taking into account sources of potential bias or imprecision. Discuss both direction and magnitude of any potential bias	14
Interpretation	20	Give a cautious overall interpretation of results considering objectives, limitations, multiplicity of analyses, results from similar studies, and other relevant evidence	13-14
Generalisability	21	Discuss the generalisability (external validity) of the study results	14
**Other information**			
Funding	22	Give the source of funding and the role of the funders for the present study and, if applicable, for the original study on which the present article is based	16

*Give information separately for exposed and unexposed groups.

Note: An Explanation and Elaboration article discusses each checklist item and gives methodological background and published examples of transparent reporting. The STROBE checklist is best used in conjunction with this article (freely available on the Web sites of PLoS Medicine at http://www.plosmedicine.org/, Annals of Internal Medicine at http://www.annals.org/, and Epidemiology at http://www.epidem.com/). Information on the STROBE Initiative is available at http://www.strobe-statement.org.

Confidential: For Review Only

Variable	Not at risk (n=1,558, 56%)	At risk (FGLD) (n=884, 44%)	Total sample (n=2,003, 100%)
**Categorical variables N (%)**			
**Maternal education**			
No qualifications	70 (6)	65 (7)	135 (7)
5 or less GCSEs	234 (21)	154 (17)	388 (19)
5 or more GCSEs	94 (8)	68 (8)	162 (8)
A levels	87 (8)	61 (7)	148 (7)
Degree	273 (24)	141 (16)	414 (21)
Missing	*361 (32)*	*395 (45)*	*756 (38)*
**Financial security**			
Finding it quite/very difficult	46 (4)	48 (5)	94 (4)
Just about getting by	132 (12)	115 (5)	247 (12)
Doing alright	368 (33)	290 (33)	658 (33)
Living comfortably	324 (29)	189 (21)	513 (26)
Missing	*249 (22)*	*242 (27)*	*491 (25)*
**Maternal ethnicity**			
White British	95 (8)	76 (9)	171 (9)
Pakistani	581 (52)	398 (45)	979 (49)
South Asian	79 (7)	64 (7)	143 (7)
White Other	42 (4)	23 (3)	65 (3)
Other	91 (8)	60 (7)	151 (8)
Missing	*231 (21)*	*263 (30)*	*494 (25)*
**Maternal county of birth**			
UK	415 (37)	322 (36)	737 (37)
Not UK	476 (43)	304 (34)	780 (39)
Missing	*228 (20)*	*258 (19)*	*486 (24)*
**Maternal language spoken**			
English	514 (46)	354 (40)	868 (43)
Not English	354 (32)	254 (29)	608 (30)
Missing	*251 (22)*	*276 (31)*	*527 (26)*
**Child sex**			
Male	621 (55)	375 (42)	996 (50)

Confidential: For Review Only

Female	498 (45)	509 (58)	1007 (50)
Missing	*0 (0)*	*0 (0)*	*0 (0)*
**Continuous variables M (SD)**			
**Child age (n=2003)**	28 (3.10)	29 (3.78)	28.62 (3.45)
**Social ladder (n=700)**	6.27 (1.79)	6.03 (1.72)	6.17 (1.76)
**People to count on (n=1070)**	3.29 (2.49)	3.27 (2.44)	3.28 (2.47)

Supplementary table. Descriptives for all participants by whether they were categorised as at risk or not, using the FGLD method.

Confidential: For Review Only

Predictor (n in model, R^2)	Odds ratio (95% CI)	*p*
**Maternal education (n=1,214, R^2=0.046)**		
No qualifications (ref)	.	.
5 or less GCSEs	1.54 (1.02 to 2.34)	0.040*
5 or more GCSEs	1.62 (0.98 to 2.68)	0.059
A levels	1.38 (0.82 to 2.33)	0.224
Degree	1.95 (1.28 to 2.99)	0.002*
**Financial security (n=1,159, R^2=0.049)**		
Finding it quite/very difficult (ref)		
Just about getting by	1.35 (0.76 to 2.42)	0.305
Doing alright	1.44 (0.84 to 2.46)	0.181
Living comfortably	1.78 (1.03 to 3.07)	0.037*
**Social status (ladder) (n=791, R^2=0.056)**	1.11 (1.02 to 1.22)	0.019*
**Social support (people to count on) (n=1,191, R^2=0.045)**	0.99 (0.94 to 1.04)	0.655

Supplementary Table. Odds ratios for adjusted logistic regression models, with no interaction for ethnicity included.

Note: *each model contained the relevant adjustment sets as presented in Table 1, though these coefficients are not presented as these coefficients can be misinterpreted (Table 2 Fallacy, see [39]).*

Adjustment sets for each model were as follows: (1) maternal education exposure included Migrant to UK, English is first language, Ethnic group, (2) financial security exposure included Maternal education, Migrant to UK, English is first language, Ethnic group, (3) social status exposure included Maternal education, Migrant to UK, English is first language, Ethnic group, (3) social support exposure included Maternal education, Migrant to UK, English is first language, Ethnic group. All models adjusted for child age and child sex.

Confidential: For Review Only

**Predictor**	**Odds ratio (95% CI)**	**p**
**Maternal education (n=1214, R^2=0.052)**		
No qualifications (ref)	.	.
5 or less GCSEs	2.07 (0.70 to 6.48)	0.193
5 or more GCSEs	1.49 (0.49to 4.74)	0.485
A levels	3.47 (0.81 to 17.17)	0.105
Degree	4.90 (1.48 – 17.57)	0.011*
**Financial security (n=1159, R^2=0.052)**		
Finding it quite/very difficult (ref)		
Just about getting by	1.38 (0.22 to 8.54)	0.720
Doing alright	1.06 (0.18 to 6.14)	0.943
Living comfortably	2.01 (0.33 to 12.33)	0.432
**Social status (ladder) (n=791, R^2=0.058)**	1.21 (0.93 to 1.58)	0.168
**Social support (people to count on) (n=1191, R^2=0.050)**	1.07 (0.93 to 1.24)	0.336

Supplementary table. Model results when interaction by ethnicity is included.

Note: Adjustment sets for each model were as follows: (1) maternal education exposure included Migrant to UK, English is first language, Ethnic group, (2) financial security exposure included Maternal education, Migrant to UK, English is first language, Ethnic group, (3) social status exposure included Maternal education, Migrant to UK, English is first language, Ethnic group, (3) social support exposure included Maternal education, Migrant to UK, English is first language, Ethnic group. All models adjusted for child age and child sex.

Confidential: For Review Only

BMJ Paediatrics Open

Do socioeconomic inequalities contribute to the high prevalence of child developmental risk in an ethnically diverse, socioeconomically disadvantaged population? A Born in Bradford's Better Start (BiBBS) study

Journal:	*BMJ Paediatrics Open*
Manuscript ID	bmjpo-2025-003770.R2
Article Type:	Original research
Date Submitted by the Author:	25-Nov-2025

Complete List of Authors:	Mooney, Kate; University of York, Health Sciences; Bradford Institute for Health Research,<br>Dickerson, Josie; Bradford Institute for Health Research<br>Blower, Sarah Louise; University of York, Health Sciences<br>Walker, Matthew; Bradford Institute for Health Research<br>Lister, Jennie; University of York, Health Sciences<br>Pickett, Kate; University of York, Health Sciences

Keywords:	Child Health, Epidemiology

SCHOLARONE™
Manuscripts

I, the Submitting Author has the right to grant and does grant on behalf of all authors of the Work (as defined in the below author licence), an exclusive licence and/or a non-exclusive licence for contributions from authors who are: i) UK Crown employees; ii) where BMJ has agreed a CC-BY licence shall apply, and/or iii) in accordance with the terms applicable for US Federal Government officers or employees acting as part of their official duties; on a worldwide, perpetual, irrevocable, royalty-free basis to BMJ Publishing Group Ltd ("BMJ") its licensees and where the relevant Journal is co-owned by BMJ to the co-owners of the Journal, to publish the Work in this journal and any other BMJ products and to exploit all rights, as set out in our licence.

The Submitting Author accepts and understands that any supply made under these terms is made by BMJ to the Submitting Author unless you are acting as an employee on behalf of your employer or a postgraduate student of an affiliated institution which is paying any applicable article publishing charge ("APC") for Open Access articles. Where the Submitting Author wishes to make the Work available on an Open Access basis (and intends to pay the relevant APC), the terms of reuse of such Open Access shall be governed by a Creative Commons licence – details of these licences and which Creative Commons licence will apply to this Work are set out in our licence referred to above.

Other than as permitted in any relevant BMJ Author's Self Archiving Policies, I confirm this Work has not been accepted for publication elsewhere, is not being considered for publication elsewhere and does not duplicate material already published. I confirm all authors consent to publication of this Work and authorise the granting of this licence.

Confidential: For Review Only

TITLE PAGE

Do socioeconomic inequalities contribute to the high prevalence of child developmental risk in an ethnically diverse, socioeconomically disadvantaged population? A Born in Bradford's Better Start (BiBBS) study

Confidential: For Review Only

Kate E. Mooney (1,2)*, Josie Dickerson (2), Sarah L. Blower (1), Matthew Walker (2), Jennie Lister (1), and Kate E. Pickett (1).

1. Department of Health Sciences, University of York
2. Bradford Institute for Health Research, Bradford Teaching Hospital Foundation Trust

**=corresponding author*

ABSTRACT

Background

Socioeconomic inequalities in child development are pervasive, however, less is known regarding the impacts of socioeconomic factors within and across ethnically diverse, socioeconomically disadvantaged populations. This study (1) describes the prevalence of children at risk of poor overall early child development; (2) investigates the relationship between individual indicators of socioeconomic position and early child development; and (3) investigates if the relationship between indicators of socioeconomic position and early child development varies by ethnic group.

Methods

This study uses data from a prospective birth cohort study, Born in Bradford's Better Start (BiBBS). Child development was measured with the Ages and Stages Questionnaire (ASQ) during routine health visiting at age 2-years-old. Binary logistic regression investigated child development by key maternal socioeconomic indicators; maternal education, financial security, social status (measured via the MacArthur Scale of Subjective Social Status), and social support (measured via number of people to count on).

Results

22% of the 2,003 children with a valid developmental assessment were at risk of poor child development. Mothers who had a degree (OR=2.09, 95% CI 1.33 to 3.30), reported 'living comfortably' in financial security (OR=1.90, 95% CI 1.08 to 3.36), and had higher social status (OR=1.13, 1.03 to 1.24) all had higher odds of their child having a good development. Though socioeconomic gradients in maternal education and financial security were consistent across White British, South Asian, and Other ethnic groups, both social support and social status had weaker relationships with child development for South Asian parents.

Conclusion

A high proportion of children are at risk of poor development in this diverse, socioeconomically disadvantaged population. Higher socioeconomic position may protect against poor early development, and the mechanisms underlying this may differ by ethnicity. The findings underline the need for proportionate universal strategies to improve child development in such communities.

Confidential: For Review Only

What is already known on this topic

- Socioeconomic inequalities in early child development are pervasive
- Whilst objective socioeconomic indicators such as higher parental education are established predictors of child development, less is known regarding subjective indicators such as social support and subjective social status, particularly within ethnically diverse populations.

What this study adds

- A high proportion (22%) of children in the socioeconomically disadvantaged and ethnically diverse Born in Bradford's Better Start (BiBBS) study population are at risk of poor child development.
- Higher maternal education, financial security, and subjective social status are associated with better child development, with the steepest socioeconomic gradient by subjective social status
- The relationships between social support and subjective social status with child development varied by ethnic group, as these did not relate to child development for South Asian parents.

How this study might affect research, practice or policy

- The BiBBS cohort can be used to further understand patterns of childhood inequalities in socioeconomically deprived, ethnically diverse populations
- Future research should focus on how and why various socioeconomic factors impact child development within socioeconomically deprived, ethnically diverse populations
- The psychological impacts associated with low subjective social status could be addressed through participation in group-based parenting programmes
- A proportionate universal approach, with both universal support available to all parents and targeted intervention programs for those in need of additional support, is recommended to improve children's outcomes.

Confidential: For Review Only

INTRODUCTION

The first three years of life are a critical period for childhood development, which encompasses the development of physical, mental, social, and emotional capabilities [1,2]. Early childhood development promotes future educational achievement and health [3,4], and persistent difficulties with early cognitive and socioemotional development can lead to worse physical and mental health in late adolescence [5]. Prevention and early intervention to promote strong early development for all children may reduce socioeconomic disparities across the lifespan [6].

Socioeconomic position and child development

Health inequalities are unfair and avoidable differences related to personal, demographic and socioeconomic characteristics. The majority of the relevant literature demonstrating pervasive socioeconomic inequalities in early child development in the UK is based on cohort studies, across various socioeconomic exposures and multiple domains of child development [7]. The Millennium Cohort Study (MCS) (N=19,517), is a nationally representative sample of children born 2000–2002. In MCS, outcomes examined at age 3 include socioemotional difficulties measured via the Strengths and Difficulties Questionnaire (SDQ) and cognitive development measured via the Bracken Basic Concept Scale. At age 5, socioemotional difficulties were measured again via the SDQ, and verbal ability via the British Ability Scales. In MCS, higher parental education predicted higher verbal ability at age 5 [8], and higher family socioeconomic position predicted lower socioemotional difficulties at ages 3 and 5 [9]. Families with the lowest incomes had children with a higher prevalence of both socioemotional and cognitive difficulties at both 3-years-old and 5-years-old [10]. A study using the Avon Longitudinal Study of Parents and Children (ALSPAC, N=13,855) similarly reported that lower parental occupation, education, and financial security were associated with lower expressive and receptive language at 2 years old measured as a modification of the MacArthur Communicative Development Inventory (CDI) [11].

Socioeconomic position may impact children's development through multiple mechanisms, for instance, socioeconomic advantage may afford parents better resources and education for enhancing the home environment of children, including via educational inputs such as language, play and stimulation, and emotional inputs such as parent-child relationships and interaction [12]. On the other hand, socioeconomic disadvantage may have a negative impact upon children's development through poor parental mental health and stress impacting upon a parent's engagement and relationship with their child [12–14]. Subjective social status captures a person's perception of their social class relative to other people, and may impact on children's outcomes through a heightened experience of chronic stress responses [15]. It is therefore of interest to compare the magnitude of associations for multiple measures, including not only objective measures such as income and education, but also subjective aspects of societal position [16]. This can give insights into the mechanisms by which family socioeconomic position influences children's development [17]. Despite this, much of the previously summarised literature applies objective measures of socioeconomic position such as education, occupation, or income [8–11].

Subjective measures

For socioeconomically deprived families, subjective measures may be more sensitive predictors of children's outcomes than measures of income, as they can more accurately assess whether lower-income families feel they can provide for basic needs [17]. For example, in the Born in Bradford cohort, parents' self-reported financial security more strongly predicted birth outcomes, in comparison to parental education, receipt of means-tested benefits, and paternal occupation [18]. Further, subjective social status indicators such as the Macarthur Scale of Subjective Social Status may encourage consideration of perceptions of relative inequality, and this has been associated with cognitive function and self-rated health in adulthood [19]. Within

Confidential: For Review Only

the sample used in this study, most pregnant women rate themselves as having higher subjective social status than others in their neighbourhood, despite living in socioeconomically deprived areas [15]. Whilst self-reported social status appears to be related to adult wellbeing [20], it is yet to be investigated with regards to children's outcomes.

Parental social support refers to the existence of social networks [21]. Although social support is not directly a measure of a family's socioeconomic position, it can be considered a specific manifestation of social capital, which refers to the resources that are obtained via a person's social networks [22,23]. Parental social support could alleviate challenges during the transition to parenthood, hence supporting childhood outcomes [21], and could potentially mitigate against the negative effects of socioeconomic disadvantage for socioeconomically disadvantaged families. Emerging evidence suggests that higher levels of social support during pregnancy are associated with improved cognitive development at age 2 [24], and lower prevalence of developmental delay at age 3 [25].

Ethnicity and child development

As ethnic minority groups tend to experience higher levels of socioeconomic disadvantage in England [26], a consideration of differences in child development by ethnicity might reveal differential impacts of socioeconomic position. Indeed, an MCS study revealed that most ethnic minority groups have lower scores in cognitive and socioemotional outcomes compared to White British children [12]. A study of routinely collected health visiting data between 2018-2021, with a sample of 226,505 children at age 2-2 ½ years, found that 86% of children met expected levels of development overall, but that children from the most deprived neighbourhoods (82.6%), and children recorded as Black ethnicity (78.9%) were less likely to meet expected level of development [27].

Socioeconomic gradients within ethnic groups

Previous research has demonstrated that socioeconomic differences appear to be less pronounced in ethnic minority groups than ethnic majority groups for maternal and child health outcomes when comparing Pakistani born to UK born mothers [18,28], child socioemotional difficulties when comparing White to ethnic minority groups [29] and working memory when comparing Pakistani to White British children [30]. This lack of socioeconomic gradient may be due to difficulties with measurement, for instance inaccurate measurements of socioeconomic position in ethnic minority groups (e.g., educational attainment if received in a different country), or smaller samples in minority groups being underpowered to detect associations. Or, these findings may be due to a true lack of relationship between socioeconomic position and children's outcomes for ethnic minority groups. Subjective measures of socioeconomic position may aid our understanding of relationships between socioeconomic position and child outcomes for ethnic minority groups, as they may be more relevant than other measures (e.g. education), and may also provide an insight into the mechanisms by which minority families mitigate against the negative effects of disadvantage. Though, the relationship between ethnicity and children's outcomes is likely to be influenced by a variety of other factors beyond socioeconomic position, such as differences in culture, and experiences of prejudice, discrimination and racism [31]. Relevant to the sample included in this study, South Asian families parenting styles may differ to other ethnic groups, and South Asian families are more likely to involve intergenerational or extended support in parenting itself [32–34]. The relationship between socioeconomic experiences and children's outcomes may therefore differ between ethnic groups, and this relationship may be mitigated or exacerbated by different cultural practices, such as parenting. Hence, larger samples of ethnic minorities, and a variety of socioeconomic measures, are needed to investigate the true impacts of socioeconomic position across diverse ethnic groups.

Confidential: For Review Only

Present study and rationale

The present study uses data from the Born in Bradford's Better Start (BiBBS) interventional birth cohort, which has recruited a representative sample of 5,758 pregnant mothers and their children living in three inner-city areas of Bradford. BiBBS recruited families throughout the time period that 'Better Start' interventions were being delivered (1st January 2016 to 31st July 2024). Whilst BiBBS was specifically designed to undertake efficient and pragmatic evaluations of these multiple early life interventions, the cohort data can also be used for understanding how inequalities develop in disadvantaged populations. The mothers in the BiBBS cohort are predominantly from ethnic minority backgrounds (88%), and live in the most deprived decile of the Index of Multiple Deprivation (84%) [35].

This under-researched population provides the opportunity to understand potential differences in children's development by both subjective and objective socioeconomic measures, enabling an investigation of whether socioeconomic differences exist within disadvantaged contexts, and among different ethnic groups. This may also provide insights into which factors may protect against the development of poor childhood development in a predominantly socioeconomically disadvantaged population, and which groups of children may need more support. Building on previous research which has tended to focus on one specific aspect of child development at a time, we use the Ages and Stages Questionnaire, which is a broad measure of child development that encompasses communication, fine and gross motor skills, problem solving, and personal-social development. In this study we:

1. Describe the prevalence of children at risk of poor child development overall
2. Investigate which individual indicators of socioeconomic position predict early child development
3. Investigate if the association between individual indicators of socioeconomic position and early child development varies by ethnic group

Confidential: For Review Only

METHODS

Study design

This is a secondary analysis of observational longitudinal cohort study data. We have used the Strengthening the Reporting of Observational Studies in Epidemiology (STROBE) (2021) guidelines for reporting methods (see Supplementary File A) [36].

Study setting

This study is set in Bradford, a city in Northern England with high levels of socioeconomic deprivation and a large ethnic minority population [37]. Part of the Born in Bradford (BiB) research programme, Born in Bradford's Better Start (BiBBS) captures in-depth baseline data during pregnancy, and participants provide written consent for routine linkage to their own and their children's health, education, and intervention participation records. Pregnant women were invited to join BiBBS by trained researchers in person. Recruitment took place predominantly at Bradford Royal Infirmary's Glucose Tolerance Test (GTT) clinic at 24-28 weeks gestation which is offered to all women booked for delivery at BRI, and secondary recruitment sources in the community [35,38]. An interim BiBBS cohort profile for mothers recruited between 2016 and 2020 was shown to be broadly representative of the pregnant population in terms of ethnicity, parent age, parity, language ability, and area deprivation [35].

Data

All predictive measures were measured during the BiBBS baseline questionnaire, administered when mothers were approximately 26 weeks pregnant with their child. We linked Health Visiting records to provide the Ages and Stages Questionnaire (ASQ) outcome for this study which was collected approximately 2-2.5-years post birth. Inclusion for this study was all recruited BiBBS pregnancies between January 2016 and December 2022, who had an ASQ recorded for that pregnancy at the 2-2 ½ year visit by October 2024 (the time of the data drop from the data source).

Inclusion of covariates was decided using a Directed Acyclic Graph (DAG) (see below), and the analytical form of variables is described below.

Measures

We used four indicators of socioeconomic position. First, maternal education was categorised into 'No qualifications', '5 or less GCSEs', '5 or more GCSEs', 'A levels', and 'Degree'. Second, financial security was ascertained by mother's response to "how well are you managing financially?" Responses were coded as: 'Just about getting by', 'Finding it quite/very difficult', 'Doing alright', 'Living comfortably'. Third, subjective social status was measured via the MacArthur Scale of Subjective Social Status, an image of a ladder with 10 rungs, numbered from 1 at the bottom to 10 at the top. We used mother's responses to "Where would you place yourself on the ladder in relation to other people in your neighbourhood?", with answers analysed as continuous, ranging between 0 and 10. Fourth, social support was measured via responses to "how many people in your life can you count on in times of need?", again analysed as a continuous predictor, and answers ranged from 0 to 10. To test the sensitivity of the results to the linearity assumption, we estimated additional models with subjective social status and social support estimated as categorical variables, categorised into low (0-2 people, 1-2 ladder), medium (3-5 people, 3-8 ladder) and high (6 to 10 people, 9-10 ladder). Any 'do not wish to answer' and 'don't know' responses were recoded as missing. The lowest socioeconomic category was used as the reference category.

https://mc.manuscriptcentral.com/bmjpo

For objective 2, maternal ethnicity was grouped into the largest ethnic groups within the cohort: White British, Pakistani heritage, White Other (containing White Irish, Polish, Slovakian, Romanian, Czech, Other White, Gypsy/Roma/Irish Traveller), Other South Asian (Indian & Bangladeshi), and Other (Chinese, African, Caribbean, Mixed, or Other). For objective 3, ethnic categories were further collapsed. Hence the groups were White British, South Asian (Pakistani, Indian & Bangladeshi) and Other. South Asian ethnicities were grouped as one due to potential cultural similarities in parenting style amongst these groups [32–34], and similarities in historic migration patterns [39]. We grouped the 'Other' ethnicities and retain them as (1) the sample size of the ethnic minority groups were too small to estimate separately, and (2) such groups have historically been excluded from research and analysis [40]. Hence, although the 'Other' group contain multiple heterogenous ethnic groups with a variety of experiences, including their data may allow for an insight into patterns in their outcomes, and generate further hypothesis testing with larger samples. White British were the reference group.

There were three binary covariates: (1) Maternal country of birth was analysed as Not born in the UK/Born in the UK, (2) Maternal first spoken language was analysed as Not English/English, and (3) Child sex was analysed as Female/Male. There was one continuous covariate, (4) child age in months at the date of the most recent ASQ.

Outcome measure (Child development via Ages and Stages Questionnaire (ASQ))

All parents living in England receive a visit from their health visitor when their child turns 2-years-old, during which children's development is assessed using the 24, 27, or 30 month Ages and Stages Questionnaire (ASQ). ASQ-3 was developed to screen for developmental delay and comprises 21 age specific questionnaires for children aged between 1 month and 66 months [41]. ASQ-3 covers five key domains of developmental status of a child's following areas [42]: Communication - babbling, vocalizing, listening, and understanding; Gross Motor: arm, body, and leg movements; Fine Motor: hand and finger movements; Problem Solving: learning and playing with toys; and Personal-Social: solitary social play and play with toys and other children. Several studies have shown the ASQ-3 to be a valid screening tool to identify developmental concerns for individual children, though there is a lack of its validation in England specifically, and for ethnic minority groups [43,44].

There are three response options to each question (yes/sometimes/not yet, with scores being yes=10, sometimes=5 and not yet=0). The score of each domain ranges between 0 and 60, however each age version and domain of the ASQ has different cut-offs to categorise children into; (1) 'below cut-off', where a child requires further assessment and intervention with a professional, (2) 'monitor', where a child's development should be monitored, and (3) 'above cut off', where a child's development is on schedule. For objective 2, in line with the Public Health England method for calculating whether children have a Good Level of Development (GLD), an overall ASQ score was created by categorising as 'Not at risk'=above cut off and/or monitor on all 5 domains, and 'At risk'='below cut off' on 1 or more domains (see https://fingertips.phe.org.uk/static-reports/health-trends-in-england/England/best_start_in_life.html; [27]).

For objective 3, to create an outcome that was more sensitive to identifying children *potentially* at risk, an additional ASQ 'Full' GLD (FGLD) score was created by categorising as 'Not at risk'='above cut off' on all 5 domains and 'At risk'='below cut off' AND/OR 'monitor' on 1 or more domains. Scores were only calculated if all 5 domains were available. The FGLD method provides more variation in the outcome than the GLD method, which means it is more sensitive to identifying any existing socioeconomic gradients in outcomes.

Confidential: For Review Only

Analysis

For objective 1 we described the number of children at risk of poor development overall using both the GLD and FGLD method. For reference we compare proportions of children at risk using the GLD method to Jung et al. (2025), who described child developmental outcomes in the ASQ-3 across England.

For objectives 2 and 3, a Directed Acyclic Graph (DAG) ascertained the adjustment set for the effect of each socioeconomic indicator (maternal education, financial management, social ladder, number of people to count on). Socioeconomic variables could be either mediators or confounders of specific associations depending on the relationship of interest, hence each relationship of interest required a separate adjustment set and analysis [45]. Justification for inclusion of each confounder/covariate was based on prior literature [10,12]. We did not include area deprivation and/or area as a covariate, since the areas under study are relatively homogeneous in terms of deprivation [35].

- *Figure 1 placed here*

Figure 1. Directed Acyclic Graph with estimates of interest labelled 1-4.

Model (#) and key predictor	Confounders included in model	Covariates included in model
1. Maternal education	Migrant to UK, English is first language, Ethnic group	Child age, child sex
2. Financial management	Maternal education, Migrant to UK, English is first language, Ethnic group	Child age, child sex
3. Subjective social status (social ladder)	Maternal education, Migrant to UK, English is first language, Ethnic group	Child age, child sex
4. Social support (people to count on)	Maternal education, Migrant to UK, English is first language, Ethnic group	Child age, child sex

Table 1. Model number with key predictor, and covariates included in each model

For objective 1, the prevalence of children at risk of poor development was described using the GLD method. For objectives 2-3, binary logistic regression models were conducted to produce odds ratios and 95% confidence intervals using the FGLD method, and model-based estimates (ie. adjusted predictions) for the outcomes were presented using plots.

For objective 3, to investigate if socioeconomic differences in child development varied by ethnic group, we estimated four additional binary logistic models with ethnicity (categorised as White British, South Asian, and Other) and each socioeconomic predictor (maternal education, financial management, social ladder, and people to count on), with an interaction fitted between these variables. We applied the specified adjustment sets for each predictor using Table 1. A likelihood ratio test was performed to compare the fit of the models with and without the interaction by ethnic group, where the fit of the two competing models was compared and the statistic represents a direct comparison of the relative likelihood of the data, given the best fit of the two models [46].

Results were considered to be statistically significant if $p<.05$. R was used for all analysis, key packages included *tidyverse, ggplot2, ggdist*, and *ggeffects*. Full analysis code and results are available at

Confidential: For Review Only

https://osf.io/c9rup/overview?view_only=0bc318a438d54db98b01787ba13462c4 (link to be replaced with public link upon acceptance of paper).

Missing data

We applied complete case analysis, as this gives unbiased results when the chance of being a complete case does not depend on the outcome after taking the covariates into consideration [47]. The analysis therefore assumes that having complete data does not depend on a child's development, after taking into account the included predictor and control variables.

Patient and public involvement

Members of the community are involved in the design and conduct of BiBBS cohort research, including recruitment, questionnaires, measures and interpretation and dissemination of findings, through regular Community Research Advisory Group meetings, composed of Better Start Bradford residents.

Confidential: For Review Only

RESULTS

Full analysis code and results are available at https://osf.io/c9rup/overview?view_only=0bc318a438d54db98b01787ba13462c4 (link to be replaced with public link upon acceptance of paper).

Sample

- *Figure placed here*

Figure 2. Eligible participants and numbers included in models for each objective

Figure 2 shows the number of pregnancies with ASQ records, and the sample sizes for each objective and model. The sample contained 2887 pregnancies with a 2-year visit recorded, and 2100 (72%) of them has an ASQ recorded. It is beyond the scope of this current study to explore reasons behind non completion of the ASQ, however, a mixed methods study by our team will explore this further [48]. The analysis sample for objective 1 contains all pregnancies with valid ASQs (n=2003), and the analysis sample for objectives 2-3 depends on the availability of the data within the sample (social status n=791, financial security n=1159, social support n=1191, and maternal education n=1214).

Objective 1 (prevalence of children at risk)

Objective 1 was to estimate the number of children at risk of poor child development overall, and the number of children at risk of poor development in each domain. Within the 2,003 individual children with linked ASQs, 445 (22%) of all children were at risk of overall poor development using the GLD method, and 884 (44%) were at risk using the FGLD method.

Objective 2 (which socioeconomic indicators predict child development)

Objective 2 was to investigate the association between individual indicators of socioeconomic position and early child development. The FGLD method was used as the outcome, which is more sensitive to identifying children potentially at risk of poor development (44%). Descriptives for all participants are presented in Supplementary Material B, and logistic regression models are presented in Supplementary Material C.

Odds ratios from adjusted logistic regressions between the predictor variables and achieving the expected level of development show that having a degree (OR=1.95, 95% CI 1.28 to 2.99), living comfortably (OR=1.78, 95% CI 1.03 to 3.07), and subjective social status (OR=1.11, 95%CI 1.02 to 1.22) all resulted in increased odds of having a FGLD. Social support was not associated with having a FGLD (OR=0.99, 95% CI 0.94 to 1.04).

- *Figures 3a-3d placed here as one –*

Figure 3a-3d. Adjusted predictions for models 1-4, with no interaction for ethnicity included.

Note: the overlap of 95% CIs in Figures a and b does not mean that the difference between any given pair of estimates is not significant at p<.05.

All socioeconomic predictors, except for social support, were significantly associated with the probability of expected development, and the figures visualise the socioeconomic gradients. Figures 3a-3d present the adjusted predictions, showing that the socioeconomic gradient is steepest by subjective social status, with the largest difference between the highest and lowest socioeconomic category for this predictor.

Confidential: For Review Only

Objective 3 (variation between socioeconomic position and child development by ethnic group)

Objective 3 was to investigate if the association between individual indicators of socioeconomic position and early child development varies by ethnic group. Results from the likelihood ratio tests indicated that the model with the interaction fitted the data equally well for maternal education (X^2=8.22, p=0.222) and financial security (X^2=4.34, p=0.461), whereas the model with the interaction was superior for subjective social status (X^2=2.90, p<0.001) and social support (X^2=6.26, p<0.001). Figures 4a-4d present the adjusted predictions, indicating that subjective social status and support were less strongly related to child development for South Asian groups, in comparison to White British and Other ethnic groups. Full model results are in Supplementary File D, and adjusted predictions from each model are presented below. The sensitivity analysis including subjective social status and support as categorical predictors showed a similar pattern of results.

- *Figures 4a-4d placed here as one -*

Figure 4a-4d. Adjusted predictions for models 1-4, with interaction by ethnicity included.

Confidential: For Review Only

DISCUSSION

Our analysis of longitudinal, routinely collected data from this place-based birth cohort study reveals a concerningly high proportion of children in inner-city Bradford identified as being at risk of poor development by age two (22%). The prevalence of children at risk is considerably higher than a previously estimated average for England (14%) [27], underscoring the significant vulnerability of this specific population. This high rate of developmental risk will likely have long-lasting implications for these children's future educational attainment, health, and overall wellbeing [6].

Whilst maternal education and financial security had previously been established as important predictors of children's outcomes [7,18], our study shows the additional relevance of parent-reported social status. Subjective social status presented the steepest social gradient in children's outcomes in comparison to maternal education (18 percentage point gap), financial security (15 percentage point gap), and social support (2 percentage point gap), with a 26% percentage point gap between the highest and lowest points on the subjective social status scale. Whilst research linking subjective social status to early developmental outcomes is limited, this aligns with broader research indicating that subjective socioeconomic measures may more strongly predict birth outcomes in deprived populations, when compared to parental education [18]. This suggests that a self-reported, subjective assessment of social standing offers a more nuanced understanding of socioeconomic position within disadvantaged populations, potentially revealing how parents in deprived areas might mitigate the challenges of socioeconomic deprivation through their own perceived subjective social status.

Since this measure of subjective social status purportedly captures a person's perception of their social class relative to other people, this may indicate that a parent's perceptions of inequality has an impact on their children's outcomes through a heightened experience of chronic stress [15,49]. Since few epidemiological studies include both subjective and objective measures of socioeconomic position, further research is necessary. We propose that subjective socioeconomic measures may be particularly useful in ethnically diverse populations through potentially obtaining lower non-response rates compared to objective metrics (like exact income or foreign qualifications) that can be challenging to reliably report. Further research is needed regarding the validity and generalisability of socioeconomic measures across different ethnic groups, and their relationships with various outcomes. The lack of a significant association between social support and child outcomes contrasted with previous research that has indicated a positive relationship [24,25], however, further analysis revealed that this may be due to differences by ethnic group.

Whilst the socioeconomic gradients in maternal education and financial management appeared broadly consistent across White British, South Asian, and Other ethnic groups, a more nuanced picture emerged regarding the role of subjective social status and support. The adjusted predictions (Figures 4a-4d) revealed a relatively weaker relationship between subjective social status and support and child outcomes specifically within the South Asian group, (in comparison to the White British and Other ethnic groups). This suggests that the null result in the original social support model was perhaps driven by the weak association for South Asian groups, suggesting social support may be a protective factor for many families. Interestingly, a very strong association was apparent between social support and child development for the 'Other' ethnic group; which includes White Other (including White Irish, Polish, Slovakian, Romanian, Czech, Other White, Gypsy/Roma/Irish Traveller), Black African/Caribbean/Black British, and Mixed ethnic groups. As these groups represent the least

Confidential: For Review Only

populous ethnic minorities in the BiBBS population, it may be that social support is more important for these communities. However, it is crucial to acknowledge that the 'Other' ethnic group in this study are heterogenous and hence there may be hidden variation across ethnicities in the association between social support and child outcomes. Hence further research is needed with these smaller ethnic minorities to explore these associations in depth. The South Asian population are the most populous ethnic group within the BiBBS area, perhaps meaning that they benefit from the psychosocial resilience offered by same-ethnic density through shared culture, networks, and social capital. This may mean that the volume of social support at a more individual level is not as crucial for improving child outcomes [50,51]. The lack of association for South Asian groups aligns with existing research that has reported a lack of expected socioeconomic gradients for maternal and child outcomes among South Asian groups [18,28,30], though these previous studies used different socioeconomic indicators. Our study extends this by demonstrating that a subjective measure of social support may have different relationships with children's outcomes depending on ethnic group, and may be particularly important for less populous ethnic minorities.

However, this finding could also reflect unmeasured confounding or inaccurate measurement. For instance, our measure of social support might not capture the *quality* or *type* of support received, which could be more influential than the quantity of social connections. Given that South Asian families in this area are often characterised by close proximity to family members living in the same households [52], the nature and dynamics of this support might differ significantly from other groups. These strong familial networks could potentially buffer against the negative effects of socioeconomic disadvantage, though research in this area is lacking.

Altogether, the strong relationship between subjective social status and child outcomes may have implications for early interventions that support families facing socioeconomic disadvantage. This may suggest that early parenting interventions should not only provide educational material and financial aid to families, but also address the psychological impacts associated with low subjective social status [49]. This indicates the importance of the social environment for parents of young children, highlighting the role that group-based parenting programmes could play in building social networks and social cohesion for parents of young children [53]. However, as we found that the importance of subjective social status and support may vary by ethnic group, further research on this topic is needed with diverse ethnic groups.

Ultimately, the relationship between ethnicity and children's outcomes is of course influenced by a multitude of factors beyond socioeconomic position, such as cultural practices, prejudice and racism [31]. Early years practitioners working with families should be mindful of potential cultural nuances in how social factors influence child development within different communities. To gain a more comprehensive understanding of the true impact of socioeconomic position across diverse ethnic groups, future research should continue to employ larger samples of ethnic minorities and apply a wider array of both objective and subjective socioeconomic measures.

Strengths and limitations

Findings from BiBBS may generalise to many similar urban populations, especially those with high ethnic diversity and socioeconomic deprivation. The large number of Pakistani families recruited (e.g. n=979, 49% of the sample) improves our statistical power to detect differences and builds on previous research describing outcomes for this ethnic minority group, which may have previously been limited by smaller numbers of ethnic minorities. Whilst it is a strength that our sample on the whole is ethnically

Confidential: For Review Only

diverse, we were limited in our ability to describe associations between socioeconomic position and child outcomes within each of these groups due to small group sizes. Further, the disadvantaged population under study may be relatively homogeneous in terms of socioeconomic characteristics, reducing the potential for observing significant social gradients. It was not possible to include income as a predictor in this study, as this was not included in the baseline questionnaire due to low acceptability in this population. Some families were missing a baseline questionnaire (around 25%) due to changes to recruitment procedures during COVID, and some children were missing the ASQ outcome (28%, see Figure 1). Hence our complete case analysis is only valid under the assumption having complete data does not depend on a child's development, after taking into account the included predictor and control variables. Whilst we tentatively suggest that the associations observed here might be causal and the longitudinal design of the study facilitates this, the study is observational and hence we cannot rule out the presence of unmeasured confounders which could be driving the associations that we present.

Conclusion and implications

A high proportion of children do not meet expected levels of development in this ethnically diverse, socioeconomically disadvantaged population. Higher socioeconomic position may protect against poor early development in such communities, and the mechanism of this may differ by ethnic group. The high prevalence of children at risk can be partly explained by socioeconomic position, and this suggests a continued need for broader public health initiatives that meet the needs of families living in disadvantaged settings, such as proportionate universal approaches that encompass both universal prevention and early targeted interventions for those who need additional support [6]. This necessitates investment in early years practitioners, such as health visitors, who can work directly in these communities to identify children at risk, and connect them with relevant support services.

Confidential: For Review Only

0.7

REQUIRED STATEMENTS

Acknowledgements

The integration of research and practice in Bradford has only been possible because of the enthusiasm and commitment of staff and volunteers across children's services in Bradford. We are grateful to all Born in Bradford staff, the Better Start Bradford partnership and staff, all Better Start Bradford project teams, health professionals, local authority and voluntary and community sector organisations who have supported the integration of research into practice. We are grateful to all the families taking part in BiBBS and all members of the Community Research Advisory Group. A poster regarding this study was presented at the International Network for Research Inequalities in Child Health (INRICH) workshop in 2025.

Funding statement

This study has received funding from the National Lottery Community Fund (previously the Big Lottery Fund) as part of the A Better Start programme (Ref 10094849). The funder was not involved in the design of the study and collection, analysis, and interpretation of data nor in writing the manuscript.

SLB, JD, and KP are also supported by the NIHR Yorkshire and Humber Applied Research Collaboration (ARC-YH; Ref: NIHR200166, see https://www.arc-yh.nihr.ac.uk). The views in this publication are those of the authors and not necessarily those of the NIHR or the Department of Health and Social Care.

Competing interests

The authors have no competing interests to declare.

Authors contributions

KEM is responsible for the overall content as the guarantor. KEM conducted the analysis and drafted the manuscript. MW cleaned and linked the BiBBS baseline with the health visiting datasets. KEP, SLB, JL and JD all reviewed the manuscript and provided substantial contributions.

Ethical approval

The protocol for BiBBS recruitment and collection of routine outcome data was approved by Bradford Leeds NHS Research Ethics Committee (15/YH/0455). Research governance approval was gained from Bradford Teaching Hospitals NHS Foundation Trust. The existing ethics includes approval for both evaluation of Better Start Bradford interventions, and observational research. Hence, no further consent was sought for this study.

Data availability

Data cannot be shared publicly as they are available through a system of managed open access. Researchers interested in accessing the data can find details and procedures on the Born in Bradford website (https://borninbradford.nhs.uk/research/how-to-access-data/). Data access is subject to review by the Born in Bradford Executive, who review proposals on a monthly basis. Requests can be submitted to borninbradford@bthft.nhs.uk. Data sharing agreements are established between the researcher and the Bradford Institute for Health Research. Full analysis code and results are available

Confidential: For Review Only

at https://osf.io/c9rup/overview?view_only=0bc318a438d54db98b01787ba13462c4 (link to be replaced with public link upon acceptance of paper).

Confidential: For Review Only

REFERENCES

1 Tierney AL, Nelson CA. Brain Development and the Role of Experience in the Early Years. *Zero Three*. 2009;30:9.

2 Newman L, Judd F, Olsson CA, *et al.* Early origins of mental disorder - risk factors in the perinatal and infant period. *BMC Psychiatry*. 2016;16:1–8. doi: 10.1186/s12888-016-0982-7

3 Murrah WM, III. Comparing Self-Regulatory and Early Academic Skills as Predictors of Later Math, Reading, and Science Elementary School Achievement. *ProQuest LLC*. 2010;102.

4 Amin V, Behrman JR, Spector TD. Does more schooling improve health outcomes and health related behaviors? Evidence from U.K. twins. *Econ Educ Rev*. 2013;35:134–48. doi: 10.1016/j.econedurev.2013.04.004

5 Black M, Akanni L, Adjei NK, *et al.* Impact of child socioemotional and cognitive development on exam results in adolescence: findings from the UK Millennium Cohort Study. *Arch Dis Child*. 2025;0:1–6. doi: 10.1136/ARCHDISCHILD-2024-327963

6 Marmot M, Allen J, Goldblatt P, *et al.* The Marmot review: Fair society, healthy lives. *The Strategic Review of Health Inequalities in England Post-2010*. 2010.

7 Pillas D, Marmot M, Naicker K, *et al.* Social inequalities in early childhood health and development: a European-wide systematic review. *Pediatric Research 2014 76:5*. 2014;76:418–24. doi: 10.1038/pr.2014.122

8 Becker B. Social disparities in children's vocabulary in early childhood. Does pre-school education help to close the gap?1. *Br J Sociol*. 2011;62:69–88. doi: 10.1111/J.1468-4446.2010.01345.X

9 Flouri E, Tzavidis N, Kallis C. Area and family effects on the psychopathology of the Millennium Cohort Study children and their older siblings. *Journal of Child Psychology and Psychiatry*. 2010;51:152–61. doi: 10.1111/J.1469-7610.2009.02156.X

10 Kelly Y, Sacker A, Del Bono E, *et al.* What role for the home learning environment and parenting in reducing the socioeconomic gradient in child development? Findings from the Millennium Cohort Study. *Arch Dis Child*. 2011;96:832–7. doi: 10.1136/adc.2010.195917

11 Lai ETC, Wickham S, Law C, *et al.* Poverty dynamics and health in late childhood in the UK: evidence from the Millennium Cohort Study. *Arch Dis Child*. 2019;archdischild-2018-316702. doi: 10.1136/archdischild-2018-316702

12 Cattan S, Fitzsimons E, Goodman A, *et al.* Early childhood inequalities. *Oxford Open Economics*. 2024;3:i711–40. doi: 10.1093/OOEC/ODAD072

13 Duncan GJ, Magnuson K. Socioeconomic status and cognitive functioning: moving from correlation to causation. *Wiley Interdiscip Rev Cogn Sci*. 2012;3:377–86. doi: 10.1002/wcs.1176

Confidential: For Review Only

14 Carneiro P, Cattan S, Neves H. Theoretical and empirical perspectives on the link between poverty, parenting and children's outcomes. *Fisc Stud*. 2025;46:9–35. doi: 10.1111/1475-5890.12404

15 Moss RH, Kelly B, Bird PK, *et al.* Turning their backs on the 'ladder of success'? Unexpected responses to the MacArthur Scale of Subjective Social Status. *Wellcome Open Res*. 2023;8:11. doi: 10.12688/WELLCOMEOPENRES.18655.2

16 Galobardes B, Shaw M, Lawlor DA, *et al.* Indicators of socioeconomic position (part 1). *J Epidemiol Community Health (1978)*. 2006;60:7–12. doi: 10.1136/jech.2004.023531

17 Ursache A, Noble KG. Neurocognitive development in socioeconomic context: Multiple mechanisms and implications for measuring socioeconomic status. *Psychophysiology*. 2016;53:71–82. doi: 10.1111/psyp.12547

18 Uphoff EP, Pickett KE, Wright J. Social gradients in health for Pakistani and White British women and infants in two UK birth cohorts. *Ethn Health*. 2016;21:452–67. doi: 10.1080/13557858.2015.1091442

19 Präg P. Subjective socio-economic status predicts self-rated health irrespective of objective family socio-economic background. *Scand J Public Health*. 2020;48:707–14. doi: 10.1177/1403494820926053/SUPPL_FILE/SJP926053_SUPPLEMENTAL_MATERIAL.PDF

20 Heitz HK, Mitchell AM. Subjective Social Status and Mental Health Outcomes: The Role of Mindfulness. *J Prev Health Promot*. 2022;3:445–67. doi: 10.1177/26320770221087248

21 Wickramaratne PJ, Yangchen T, Lepow L, *et al.* Social connectedness as a determinant of mental health: A scoping review. *PLoS One*. 2022;17:e0275004. doi: 10.1371/JOURNAL.PONE.0275004

22 Villalonga-Olives E, Kawachi I. The dark side of social capital: A systematic review of the negative health effects of social capital. *Soc Sci Med*. 2017;194:105–27. doi: 10.1016/J.SOCSCIMED.2017.10.020

23 Saegert S, Carpiano RM. Social support and social capital: A theoretical synthesis using community psychology and community sociology approaches. *APA handbook of community psychology: Theoretical foundations, core concepts, and emerging challenges*. 2016;295–314. doi: 10.1037/14953-014

24 Shin EK, Lewinn K, Bush N, *et al.* Association of Maternal Social Relationships With Cognitive Development in Early Childhood. *JAMA Netw Open*. 2019;2:e186963–e186963. doi: 10.1001/JAMANETWORKOPEN.2018.6963

25 Imanishi Y, Ikehara S, Aochi Y, *et al.* The association between maternal social support levels during pregnancy and child development at three years of age: the Japan Environment and Children's Study. *Environ Health Prev Med*. 2024;29:18–18. doi: 10.1265/EHPM.23-00211

26 Chattoo S, Atkin K. *Understanding 'race' and ethnicity 2e: Theory, history, policy, practice.* 2nd ed. Policy Press 2019.

Confidential: For Review Only

27 Jung J, Cattan S, Powell C, *et al.* Early child development in England: cross-sectional analysis of ASQ®-3 records from the 2-2½-year universal health visiting review using national administrative data (Community Service Dataset, CSDS). *Int J Popul Data Sci*. 2025;9:2459. doi: 10.23889/IJPDS.V9I2.2459

28 Mallicoat B, P Uphoff E, E Pickett K. Estimating Social Gradients in Health for UK Mothers and Infants of Pakistani Origin: Do Latent Class Measures of Socioeconomic Position Help? *J Immigr Minor Health*. Published Online First: 2020. doi: 10.1007/s10903-020-00977-9

29 Kuo YL, Casillas A, Walton KE, *et al.* The intersectionality of race/ethnicity and socioeconomic status on social and emotional skills. *J Res Pers*. 2020;84:103905. doi: 10.1016/j.jrp.2019.103905

30 Mooney KE, Pickett KE, Shire K, *et al.* Socioeconomic disadvantage and ethnicity are associated with large differences in children's working memory ability: analysis of a prospective birth cohort study following 13,500 children. *BMC Psychol*. 2022;10:67. doi: 10.1186/s40359-022-00773-0

31 Marks AK, Garcia Coll C. Education and developmental competencies of ethnic minority children: Recent theoretical and methodological advances. *Developmental Review*. 2018;50:90–8. doi: 10.1016/j.dr.2018.05.004

32 Crozier G, Davies J. Family matters: A discussion of the Bangladeshi and Pakistani extended family and community in supporting the children's education. *Sociological Review*. 2006;54:678–95. doi: 10.1111/J.1467-954X.2006.00666.X;WGROUP:STRING:PUBLICATION

33 Iqbal H, Golombok S. The Generation Game: Parenting and Child Outcomes in Second-Generation South Asian Immigrant Families in Britain. *J Cross Cult Psychol*. 2018;49:25–43. doi: 10.1177/0022022117736039;SUBPAGE:STRING:FULL

34 Dwyer C, Modood T, Sanghera G, *et al.* Educational Achievement and Career Aspiration for Young British Pakistanis. *Global Migration, Ethnicity and Britishness*. Palgrave Macmillan UK 2011:177–204.

35 Dickerson J, Bridges S, Willan K, *et al.* Born in Bradford's Better Start (BiBBS) interventional birth cohort study: Interim cohort profile. *Wellcome Open Research 2022 7:244*. 2022;7:244. doi: 10.12688/wellcomeopenres.18394.1

36 Ghaferi AA, Schwartz TA, Pawlik TM. STROBE Reporting Guidelines for Observational Studies. *JAMA Surg*. 2021;156:577–8. doi: 10.1001/JAMASURG.2021.0528

37 Bradford Council. Poverty in Bradford District | Understanding Bradford District. 2019. https://ubd.bradford.gov.uk/about-us/poverty-in-bradford-district/ (accessed 1 February 2021)

38 Dickerson J, Bird PK, McEachan RRC, *et al.* Born in Bradford's Better Start: An experimental birth cohort study to evaluate the impact of early life interventions. *BMC Public Health*. 2016;16:711. doi: 10.1186/s12889-016-3318-0

Confidential: For Review Only

39 Singh R. Destination Bradford : Bradford's South Asian Community. *South Asia Res*. 1987;7:13–24. doi: 10.1177/026272808700700101

40 Gill PS, Redwood S. Under-representation of minority ethnic groups in research — call for action. *The British Journal of General Practice*. 2013;63:342. doi: 10.3399/BJGP13X668456

41 Velikonja T, Edbrooke-Childs J, Calderon A, *et al.* The psychometric properties of the Ages & Stages Questionnaires for ages 2-2.5: a systematic review. *Child Care Health Dev*. 2017;43:1–17. doi: 10.1111/CCH.12397

42 Squires J, Bricker D. Ages & Stages Questionnaires [R], (ASQ-3 [TM]): A Parent-Completed Child-Monitoring System. *Brookes Publishing Company*. 2009.

43 Kendall S, Nash A, Braun A, *et al.* Evaluating the use of a population measure of child development in the Healthy Child Programme Two Year Review. Policy Research Unit in the Health of Children, Young People and Families* Centre for Research in Primary and Community Care**. 2014.

44 Kendall S, Nash A, Braun A, *et al.* Acceptability and understanding of the Ages & Stages Questionnaires®, Third Edition, as part of the Healthy Child Programme 2-year health and development review in England: Parent and professional perspectives. *Child Care Health Dev*. 2019;45:251–6. doi: 10.1111/CCH.12639

45 Shrier I, Platt RW. Reducing bias through directed acyclic graphs. BMC Med Res Methodol. 2008;8:1–15.

46 Glover S, Dixon P. Likelihood ratios: A simple and flexible statistic for empirical psychologists. *Psychon Bull Rev*. 2004;11:791–806. doi: 10.3758/BF03196706/METRICS

47 Hughes RA, Heron J, Sterne JAC, *et al.* Accounting for missing data in statistical analyses: multiple imputation is not always the answer. *Int J Epidemiol*. 2019;48:1294–304. doi: 10.1093/IJE/DYZ032

48 Henderson HC, Mooney KE, Morton K, *et al.* Use of the Ages and stages questionnaire in Bradford as a measure of Child Development (ABCD): a mixed methods study protocol. *medRxiv*. 2025;2025.08.14.25333745. doi: 10.1101/2025.08.14.25333745

49 Singh-Manoux A, Adler NE, Marmot MG. Subjective social status: its determinants and its association with measures of ill-health in the Whitehall II study. *Soc Sci Med*. 2003;56:1321–33. doi: 10.1016/S0277-9536(02)00131-4

50 Pickett KE, Wilkinson RG. People like us: Ethnic group density effects on health. Ethn Health. 2008;13:321–34.

51 Pickett KE, Shaw RJ, Atkin K, *et al.* Ethnic density effects on maternal and infant health in the Millennium Cohort Study. *Soc Sci Med*. 2009;69:1476–83. doi: 10.1016/j.socscimed.2009.08.031

52 Thapar-Bjorkert S, Sanghera G. Social capital, educational aspirations and young Pakistani Muslim men and women in Bradford, West Yorkshire. 2010.

Confidential: For Review Only

53 Barlow J, Stewart-Brown S. Understanding parenting programmes: parents' views. *Prim Health Care Res Dev*. 2001;2:117–30. doi: 10.1191/146342301678787067

FIGURE LEGENDS

Figure 1. Directed Acyclic Graph with estimates of interest labelled 1-4.

Figure 2. Eligible participants and numbers included in models for each objective

Figure 3a-3d. Adjusted predictions for models 1-4, with no interaction for ethnicity included.

Figure 4a-4d. Adjusted predictions for models 1-4, with interaction by ethnicity included.

Confidential: For Review Only

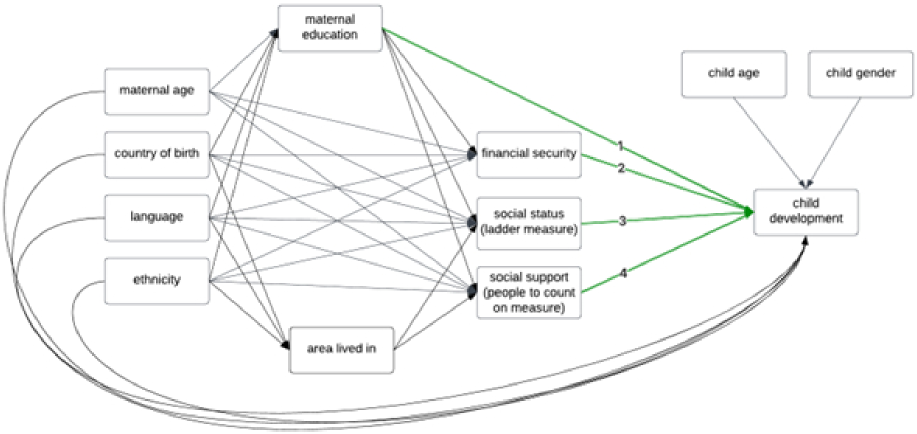

Figure 1. Directed Acyclic Graph with estimates of interest labelled 1-4.

138x63mm (118 x 118 DPI)

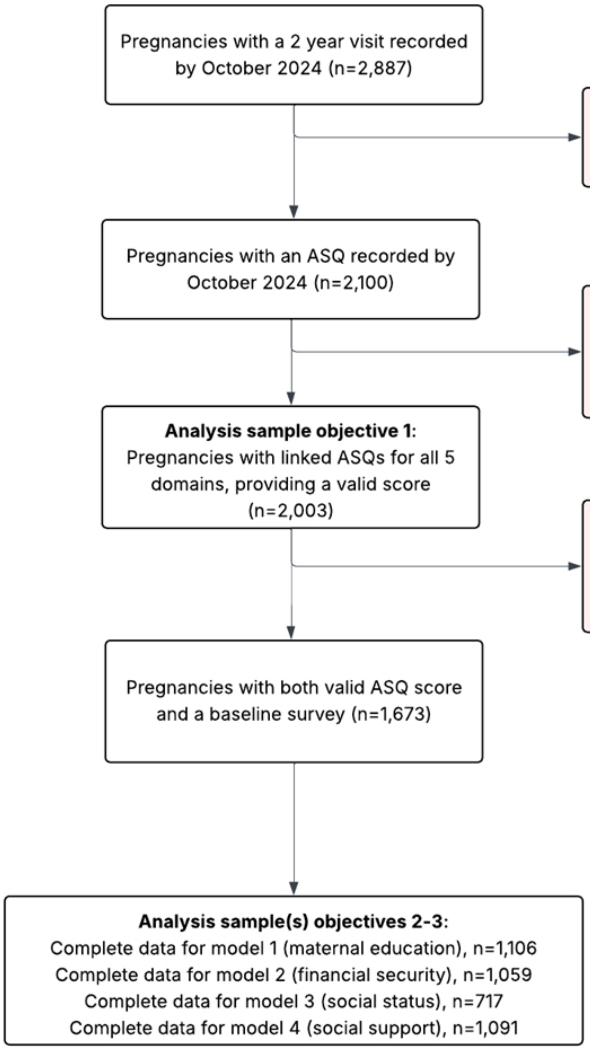

Figure 2. Eligible participants and numbers included in models for each objective

190x218mm (118 x 118 DPI)

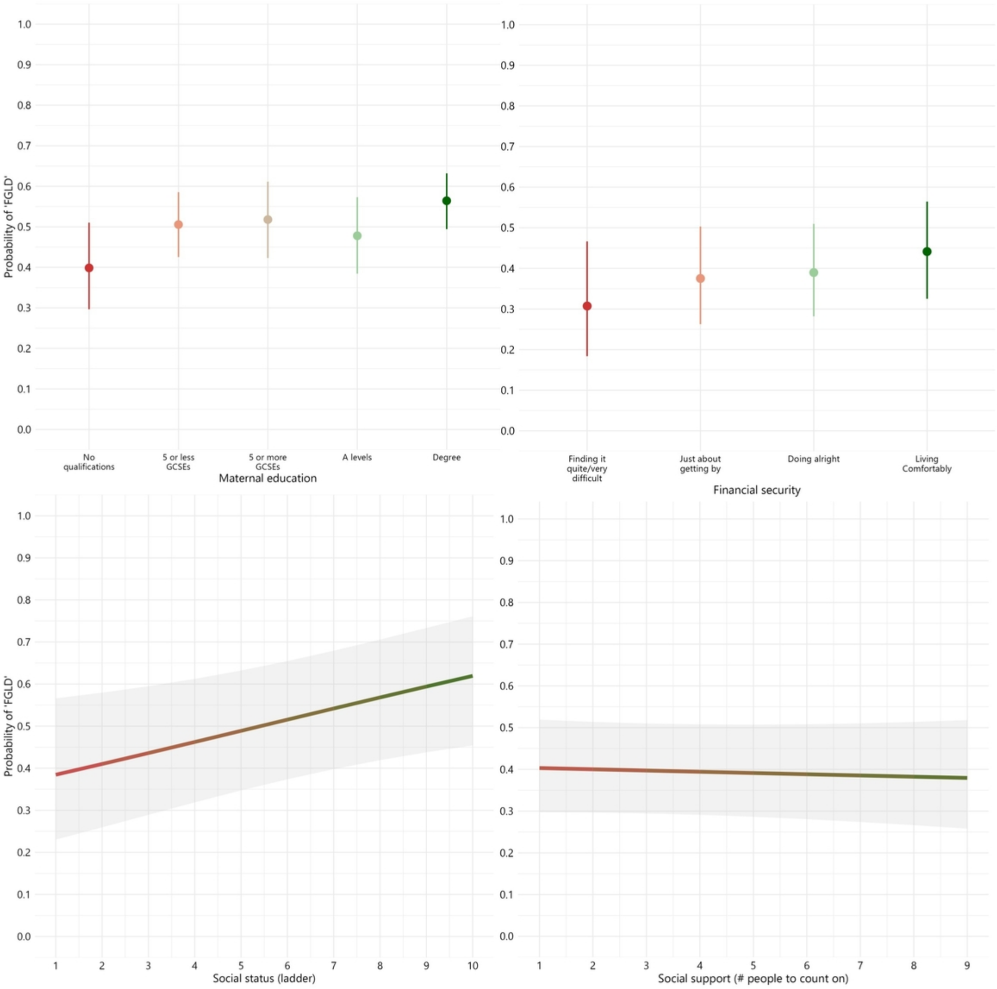

Figure 3a-3d. Adjusted predictions for models 1-4.

98x98mm (300 x 300 DPI)

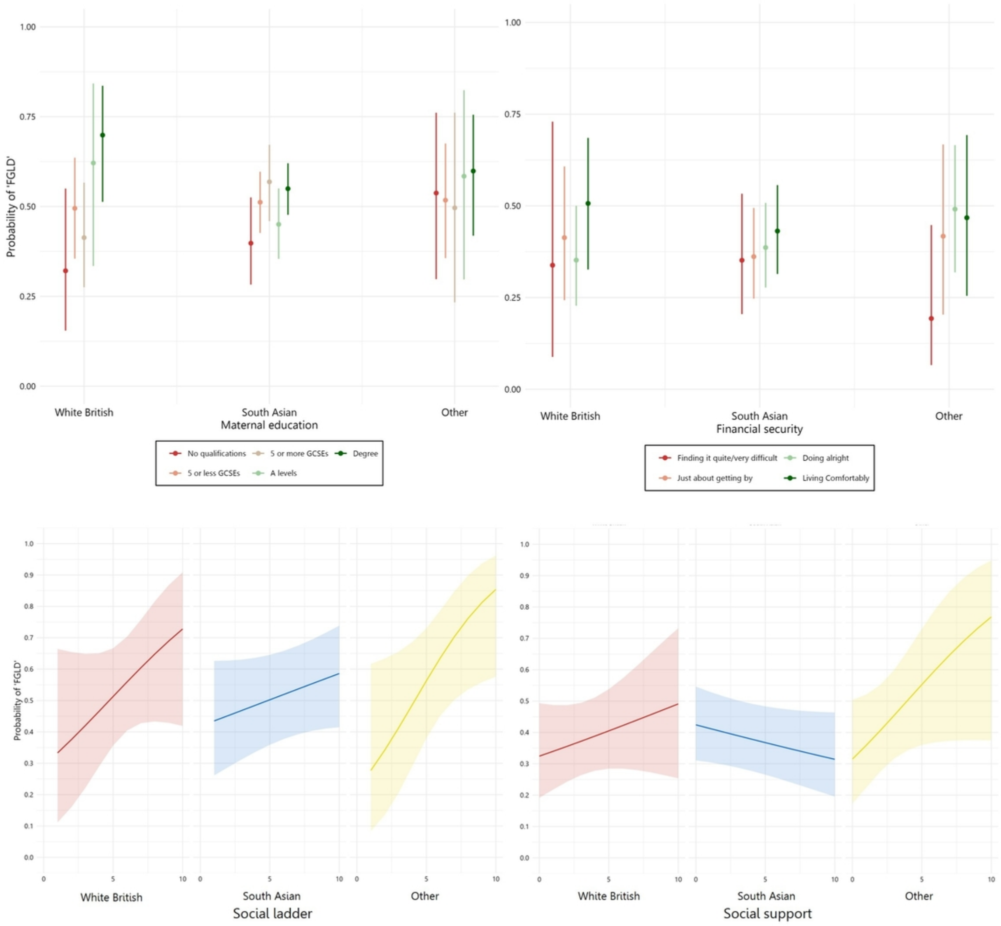

Figure 4a-4d. Adjusted predictions for models 1-4, with interaction by ethnicity included.

96x91mm (300 x 300 DPI)

STROBE Statement—Checklist of items that should be included in reports of ***cohort studies***

	Item No	Recommendation	Page No
**Title and abstract**	1	(*a*) Indicate the study's design with a commonly used term in the title or the abstract	1
		(*b*) Provide in the abstract an informative and balanced summary of what was done and what was found	1
**Introduction**			
Background/rationale	2	Explain the scientific background and rationale for the investigation being reported	4-6
Objectives	3	State specific objectives, including any prespecified hypotheses	6
**Methods**			
Study design	4	Present key elements of study design early in the paper	7
Setting	5	Describe the setting, locations, and relevant dates, including periods of recruitment, exposure, follow-up, and data collection	7-8
Participants	6	(*a*) Give the eligibility criteria, and the sources and methods of selection of participants. Describe methods of follow-up	7-8
		(*b*) For matched studies, give matching criteria and number of exposed and unexposed	NA
Variables	7	Clearly define all outcomes, exposures, predictors, potential confounders, and effect modifiers. Give diagnostic criteria, if applicable	7-8
Data sources/ measurement	8*	For each variable of interest, give sources of data and details of methods of assessment (measurement). Describe comparability of assessment methods if there is more than one group	8
Bias	9	Describe any efforts to address potential sources of bias	8-9
Study size	10	Explain how the study size was arrived at	11
Quantitative variables	11	Explain how quantitative variables were handled in the analyses. If applicable, describe which groupings were chosen and why	9
Statistical methods	12	(*a*) Describe all statistical methods, including those used to control for confounding	8-9
		(*b*) Describe any methods used to examine subgroups and interactions	8-9
		(*c*) Explain how missing data were addressed	10
		(*d*) If applicable, explain how loss to follow-up was addressed	NA
		(*e*) Describe any sensitivity analyses	NA
**Results**			
Participants	13*	(a) Report numbers of individuals at each stage of study—eg numbers potentially eligible, examined for eligibility, confirmed eligible, included in the study, completing follow-up, and analysed	11
		(b) Give reasons for non-participation at each stage	11
		(c) Consider use of a flow diagram	11
Descriptive data	14*	(a) Give characteristics of study participants (eg demographic, clinical, social) and information on exposures and potential confounders	12
		(b) Indicate number of participants with missing data for each variable of interest	12
		(c) Summarise follow-up time (eg, average and total amount)	12

Confidential: For Review Only

Outcome data	15*	Report numbers of outcome events or summary measures over time	11
Main results	16	(a) Give unadjusted estimates and, if applicable, confounder-adjusted estimates and their precision (eg, 95% confidence interval). Make clear which confounders were adjusted for and why they were included	No unadjusted estimates, 13
		(b) Report category boundaries when continuous variables were categorized	7
		(c) If relevant, consider translating estimates of relative risk into absolute risk for a meaningful time period	NA
Other analyses	17	Report other analyses done—eg analyses of subgroups and interactions, and sensitivity analyses	NA
**Discussion**			
Key results	18	Summarise key results with reference to study objectives	13
Limitations	19	Discuss limitations of the study, taking into account sources of potential bias or imprecision. Discuss both direction and magnitude of any potential bias	14
Interpretation	20	Give a cautious overall interpretation of results considering objectives, limitations, multiplicity of analyses, results from similar studies, and other relevant evidence	13-14
Generalisability	21	Discuss the generalisability (external validity) of the study results	14
**Other information**			
Funding	22	Give the source of funding and the role of the funders for the present study and, if applicable, for the original study on which the present article is based	16

*Give information separately for exposed and unexposed groups.

Note: An Explanation and Elaboration article discusses each checklist item and gives methodological background and published examples of transparent reporting. The STROBE checklist is best used in conjunction with this article (freely available on the Web sites of PLoS Medicine at http://www.plosmedicine.org/, Annals of Internal Medicine at http://www.annals.org/, and Epidemiology at http://www.epidem.com/). Information on the STROBE Initiative is available at http://www.strobe-statement.org.

Confidential: For Review Only

Variable	Not at risk (n=1,558, 56%)	At risk (FGLD) (n=884, 44%)	Total sample (n=2,003, 100%)
**Categorical variables N (%)**			
**Maternal education**			
No qualifications	70 (6)	65 (7)	135 (7)
5 or less GCSEs	234 (21)	154 (17)	388 (19)
5 or more GCSEs	94 (8)	68 (8)	162 (8)
A levels	87 (8)	61 (7)	148 (7)
Degree	273 (24)	141 (16)	414 (21)
Missing	*361 (32)*	*395 (45)*	*756 (38)*
**Financial security**			
Finding it quite/very difficult	46 (4)	48 (5)	94 (4)
Just about getting by	132 (12)	115 (5)	247 (12)
Doing alright	368 (33)	290 (33)	658 (33)
Living comfortably	324 (29)	189 (21)	513 (26)
Missing	*249 (22)*	*242 (27)*	*491 (25)*
**Maternal ethnicity**			
White British	95 (8)	76 (9)	171 (9)
Pakistani	581 (52)	398 (45)	979 (49)
South Asian	79 (7)	64 (7)	143 (7)
White Other	42 (4)	23 (3)	65 (3)
Other	91 (8)	60 (7)	151 (8)
Missing	*231 (21)*	*263 (30)*	*494 (25)*
**Maternal county of birth**			
UK	415 (37)	322 (36)	737 (37)
Not UK	476 (43)	304 (34)	780 (39)
Missing	*228 (20)*	*258 (19)*	*486 (24)*
**Maternal language spoken**			
English	514 (46)	354 (40)	868 (43)
Not English	354 (32)	254 (29)	608 (30)
Missing	*251 (22)*	*276 (31)*	*527 (26)*
**Child sex**			
Male	621 (55)	375 (42)	996 (50)

Confidential: For Review Only

Female	498 (45)	509 (58)	1007 (50)
Missing	*0 (0)*	*0 (0)*	*0 (0)*
**Continuous variables M (SD)**			
**Child age in months (n=2003)**	28 (3.10)	29 (3.78)	28.62 (3.45)
**Social ladder (n=837)**	6.27 (1.79)	6.03 (1.72)	6.17 (1.76)
**People to count on (n=1546)**	3.29 (2.49)	3.27 (2.44)	3.28 (2.47)

Supplementary table. Descriptives for all participants by whether they were categorised as at risk or not, using the FGLD method.

Confidential: For Review Only

Variable	Other	South Asian	White British	Total
**Categorical variables N (%)**				
**Maternal education**				
No qualifications	23 (11)	93 (8)	19 (11)	135 (9)
5 or less GCSEs	49 (23)	287 (26)	51 (30)	387 (26)
5 or more GCSEs	16 (7)	102 (9)	44 (26)	162 (11)
A levels	18 (8)	116 (10)	14 (8)	148 (10)
Degree	42 (19)	338 (30)	33 (19)	413 (27)
Missing	68 (31)	186 (17)	10 (6)	264 (17)
**Financial security**				
Finding it quite/very difficult	22 (10)	59 (5)	6 (4)	87 (6)
Just about getting by	36 (17)	159 (14)	37 (22)	232 (15)
Doing alright	98 (45)	447 (40)	77 (45)	622 (41)
Living comfortably	44 (20)	406 (36)	46 (26)	496 (33)
Missing	16 (7)	51 (5)	5 (3)	72 (5)
**Maternal county of birth**				
UK	45 (21)	517 (46)	170 (99)	732 (49)
Not UK	169 (78)	605 (54)	1 (<1)	775 (51)
Missing	2 (<1)	0 (0)	0 (0)	2 (<1)
**Maternal language spoken**				
English	77 (36)	615 (55)	171(100)	863
Not English	111 (51)	493 (44)	0 (0)	604
Missing	28 (13)	14 (1)	0 (0)	42
**Child sex**				
Male	114 (53)	571 (49)	85 (50)	770 (49)
Female	102 (47)	551 (51)	86 (5)	739 (51)
Missing	0 (0)	0 (0)	0 (0)	0 (0)
**Continuous variables M (SD)**				
**Child age in months (n=1508)**	28.49 (3.73)	28.01 (3.21)	28.49 (4.93)	28.13 (3.53)
**Social ladder (n=835)**	5.57 (2.07)	6.36 (1.73)	5.71 (1.44)	6.17 (1.76)
**People to count on (n=1469)**	2.69 (2.09)	3.33 (2.53)	3.70 (2.36)	3.28 (2.47)

Confidential: For Review Only

Supplementary table. Descriptives for all participants by ethnic groups used in Objective C.

Confidential: For Review Only

Predictor (n in model, R^2)	Odds ratio (95% CI)	*p*
**Maternal education (n=1,214, R^2=0.046)**		
No qualifications (ref)	.	.
5 or less GCSEs	1.54 (1.02 to 2.34)	0.040*
5 or more GCSEs	1.62 (0.98 to 2.68)	0.059
A levels	1.38 (0.82 to 2.33)	0.224
Degree	1.95 (1.28 to 2.99)	0.002*
**Financial security (n=1,159, R^2=0.049)**		
Finding it quite/very difficult (ref)		
Just about getting by	1.35 (0.76 to 2.42)	0.305
Doing alright	1.44 (0.84 to 2.46)	0.181
Living comfortably	1.78 (1.03 to 3.07)	0.037*
**Social status (ladder) (n=791, R^2=0.056)**	1.11 (1.02 to 1.22)	0.019*
**Social support (people to count on) (n=1,191, R^2=0.045)**	0.99 (0.94 to 1.04)	0.655

Supplementary Table. Odds ratios for adjusted logistic regression models, with no interaction for ethnicity included.

Note: *each model contained the relevant adjustment sets as presented in Table 1, though these coefficients are not presented as these coefficients can be misinterpreted (Table 2 Fallacy, see [39]).*

Adjustment sets for each model were as follows: (1) maternal education exposure included Migrant to UK, English is first language, Ethnic group, (2) financial security exposure included Maternal education, Migrant to UK, English is first language, Ethnic group, (3) social status exposure included Maternal education, Migrant to UK, English is first language, Ethnic group, (3) social support exposure included Maternal education, Migrant to UK, English is first language, Ethnic group. All models adjusted for child age and child sex.

Confidential: For Review Only

Predictor	Odds ratio (95% CI)	p
**Maternal education (n=1214, R^2=0.052)**		
No qualifications (ref)	.	.
5 or less GCSEs	2.07 (0.70 to 6.48)	0.193
5 or more GCSEs	1.49 (0.49to 4.74)	0.485
A levels	3.47 (0.81 to 17.17)	0.105
Degree	4.90 (1.48 – 17.57)	0.011*
**Financial security (n=1159, R^2=0.052)**		
Finding it quite/very difficult (ref)		
Just about getting by	1.38 (0.22 to 8.54)	0.720
Doing alright	1.06 (0.18 to 6.14)	0.943
Living comfortably	2.01 (0.33 to 12.33)	0.432
**Social status (ladder) (n=791, R^2=0.058)**	1.21 (0.93 to 1.58)	0.168
**Social support (people to count on) (n=1191, R^2=0.050)**	1.07 (0.93 to 1.24)	0.336

Supplementary table. Model results when interaction by ethnicity is included.

Note: Adjustment sets for each model were as follows: (1) maternal education exposure included Migrant to UK, English is first language, Ethnic group, (2) financial security exposure included Maternal education, Migrant to UK, English is first language, Ethnic group, (3) social status exposure included Maternal education, Migrant to UK, English is first language, Ethnic group, (3) social support exposure included Maternal education, Migrant to UK, English is first language, Ethnic group. All models adjusted for child age and child sex.

Confidential: For Review Only
